# Testing Domestication Scenarios of Lima Bean (*Phaseolus lunatus* L.) in Mesoamerica: Insights from Genome-Wide Genetic Markers

**DOI:** 10.3389/fpls.2017.01551

**Published:** 2017-09-12

**Authors:** María I. Chacón-Sánchez, Jaime Martínez-Castillo

**Affiliations:** ^1^Departamento de Agronomía, Facultad de Ciencias Agrarias, Universidad Nacional de Colombia Bogotá, Colombia; ^2^Centro de Investigación Científica de Yucatán Yucatán, Mexico

**Keywords:** approximate bayesian computation, SNPs, genotyping-by-sequencing, linkage disequilibrium, founder effects, domestication bottlenecks

## Abstract

Plant domestication can be seen as a long-term process that involves a complex interplay among demographic processes and evolutionary forces. Previous studies have suggested two domestication scenarios for Lima bean in Mesoamerica: two separate domestication events, one from gene pool MI in central-western Mexico and another one from gene pool MII in the area Guatemala-Costa Rica, or a single domestication from gene pool MI in central-western Mexico followed by post-domestication gene flow with wild populations. In this study we evaluated the genetic structure of the wild gene pool and tested these two competing domestication scenarios of Lima bean in Mesoamerica by applying an ABC approach to a set of genome-wide SNP markers. The results confirm the existence of three gene pools in wild Lima bean, two Mesoamerican gene pools (MI and MII) and the Andean gene pool (AI), and suggest the existence of another gene pool in central Colombia. The results indicate that although both domestication scenarios may be supported by genetic data, higher statistical support was given to the single domestication scenario in central-western Mexico followed by admixture with wild populations. Domestication would have involved strong founder effects reflected in loss of genetic diversity and increased LD levels in landraces. Genomic regions affected by selection were detected and these may harbor candidate genes related to domestication.

## Introduction

Domestication can be seen as a complex interplay among demographic processes and evolutionary forces that increase the adaptation of wild populations to human-driven environments (Purugganan and Fuller, 2009; Larson and Burger, 2013; Meyer and Purugganan, 2013; Wang et al., 2017). Several questions about domestication have been of interest to evolutionary biologists. One of these questions is the number of times a crop species was domesticated. The traditional approach to address this question has been the identification of monophyletic clusters of extant crop representatives as evidence of single domestication. However, this approach may be misleading because the extent of genetic drift and gene flow (for example among independent cultivation sites) may be effective in erasing early genetic signals of multiple domestications (Allaby et al., 2008; Olsen and Gross, 2008). A second question is the extent of the domestication bottleneck (Ladizinsky, 1985), which translates into a loss of crop genetic diversity. A third question is the geographic area where domestication took place. This is basically done by identifying the wild stocks that are most closely related to domestic populations (Salamini et al., 2002), however profuse gene flow among domesticates and wild populations should be taken into account (Cornille et al., 2012). A fourth question is time of domestication, namely when domestication occurred and how long did it take. This question may be answered by examination of archeological remains of wild and domestic forms (Purugganan and Fuller, 2011). A final question that is key for further genetic improvement of domestic forms is how domestication traits arose, namely those traits that differentiate wild from crop populations (the domestication syndrome). To answer this question it is necessary to identify genomic regions that were affected by selection and also the genes that underlie the genetic control of those traits that arose during the process of adaptation to domestication (Doebley et al., 2006).

The questions mentioned above have been traditionally addressed with genetic markers (in the terms of dozens of them) and their analysis by indirect approaches, which mainly involve calculation of genetic distances among wild and domesticated populations and visualization of patterns by means of clustering approaches, assignment tests, etc. From the interpretation of these patterns usually some hypotheses are proposed but in general these hypotheses are not tested (Gerbault et al., 2014). For domestication, as well as for other evolutionary processes, hypothesis testing may not be an easy task because scenarios may be too complex and datasets too large to be analyzed by many of the available methods based on the calculation of the likelihood function (Gerbault et al., 2014). This difficulty stimulated the development of other approximations such as the so-called Approximate Bayesian Computation (ABC) approach (Beaumont et al., 2002). Due to the genetic stochasticity of evolutionary processes and because evolutionary forces may affect different regions of the genome in a different way, sampling a relatively large number of loci distributed along chromosomes is important. Fortunately, the development of new sequencing and genotyping technologies allow the analysis of genome-wide genetic markers for evolutionary studies, even in non-model plant species (Elshire et al., 2011).

Lima bean is the second most important crop of the genus *Phaseolus* (after common bean) cultivated worldwide. The con-specific wild ancestor of Lima bean is widely distributed from Mexico to Argentina according to current germplasm and herbarium records (Debouck, 2008). Wild Lima bean is structured into three gene pools (Serrano-Serrano et al., 2010), the Mesoamerican I gene pool (MI) occurs in central-western Mexico, to the north and west of the Isthmus of Tehuantepec; the Mesoamerican II gene pool (MII) is found in Mexico to the south and east of the Isthmus of Tehuantepec, along the coastal plains of the Gulf of Mexico, in Central America, northern South America, southern Peru, Bolivia, and northern Argentina; the Andean gene pool (AI) is distributed in a narrow geographic range in the Andes of Ecuador and northern Peru.

Lima bean landraces are classified into two major groups, the Mesoamerican and the Andean, according to their geographic origin and seed characteristics. Mesoamerican landraces have small seeds (size ranging from 30 to 78 g/100 seeds, with an average of about 45 g/100 seeds), include the types known as “Sieva” (flat or kidney-shaped small seeds) and “Potato” (globular small seeds), and were domesticated in the Mesoamerica region (Gutiérrez-Salgado et al., 1995; Motta-Aldana et al., 2010). Andean landraces have flat larger seeds known as “Big Lima” (size ranging from 58 to 122 g/100 seeds, with an average of about 87 g/100 seeds) and were domesticated in the Andes of Ecuador and northern Peru (Gutiérrez-Salgado et al., 1995; Motta-Aldana et al., 2010). Previous studies, based on genetic data from few loci, have proposed two competing scenarios for the origin of the Mesoamerican landraces: (1) two separate domestication events, one from gene pool MI in central-western Mexico and another one from gene pool MII in the area Guatemala-Costa Rica, or (2) a single domestication from gene pool MI in central-western Mexico and post-domestication gene flow with wild populations from gene pool MII (Motta-Aldana et al., 2010). These previous studies have also shown that domestication was accompanied by strong founder effects that decreased genetic diversity of landraces in Mesoamerica and the Andes. Founder effects have so far been quantified with a handful of marker loci [the internal transcribed spacer of the ribosomal DNA (ITS), two non-coding regions of the chloroplast DNA (cpDNA) and a handful of nuclear SSR markers], which raises questions about how these estimations represent genome-wide patterns of diversity in Lima bean. A key aspect that has not been explored in previous studies is how domestication has affected genome-wide patterns of linkage disequilibrium in landrace populations, an aspect that undoubtedly will increase our understanding of the evolution of domesticated populations.

In spite that much have been advanced in the understanding of the evolution of this species in the wild and during domestication, previous genetic data have been based on a set of very few marker loci, which have made it impossible to test the two domestication scenarios outlined above, especially because the uniparental inheritance of the cpDNA and the very poor representation of the nuclear genome are not adequate to discern between hypotheses involving gene flow. On the light of the new sequencing technologies and the recent development of methods of analysis, not only these two hypotheses may be tested but also many aspects of the evolution of this crop species may now be addressed from a genome-wide perspective. The main goal of the present study was to test which one of the two domestication hypotheses better fit to data gathered from a set of genome-wide SNP markers genotyped at 270 accessions of wild (110) and domesticated (160) Lima bean, mainly from the Mesoamerican gene pool. Because genome-wide SNP markers provide a more complete picture of the genetic variation of Lima bean, we used these markers to assess from a genomic perspective the genetic structure of Lima bean, the effect of domestication on genome-wide diversity and patterns of linkage disequilibrium in landraces, and to detect genomic regions that may harbor candidate domestication genes.

## Materials and methods

### Plant material

On the basis of previous studies (Motta-Aldana et al., 2010; Serrano-Serrano et al., 2010, 2012; Andueza-Noh et al., 2013, 2015; Martínez-Castillo et al., 2014), 160 wild and 110 domesticated accessions were selected from the germplasm bank of the International Center for Tropical Agriculture—CIAT and the Centro de Investigación Científica de Yucatán, CICY (see Table S1). Accessions were chosen from those analyzed in previous studies and that had a gene pool assigned according to ITS data. These accessions were complemented with other accessions in order to reflect the known range of distribution of Lima bean in the Americas. Most of the selected accessions have geographic coordinate data (256 out of 270 accessions). Because in this study we are testing domestication hypotheses for the Mesoamerican landraces, the accessions analyzed in this study are mainly (not exclusively) distributed within the potential domestication area in Mesoamerica, namely from Mexico to Costa Rica. Therefore, many of the wild accessions come from countries such as Mexico (78 accessions) and Guatemala (29 accessions) and almost half of the landraces come from Mexico (46 accessions). Also some accessions from South America, classified previously within gene pool MII and the Andean gene pool, were selected. The geographic distribution of the accessions is shown in Figure S1.

### Genotyping by sequencing (GBS)

DNA was extracted from young leaflets with the method reported by Vega-Vela and Chacón Sánchez (2011). These DNA samples were analyzed by GBS (Elshire et al., 2011) by the Institute for Genome Diversity of Cornell University, USA. Sequence reads were processed and analyzed with the software NGSEP (Duitama et al., 2014; Perea et al., 2016). Sequence reads were de-multiplexed on the basis of their unique barcodes and aligned to the common bean (*Phaseolus vulgaris* L.) genome used as reference. The reference genome was obtained from Phytozome v. 9.1 (http://www.phytozome.net/) and indexed with the program Bowtie 2 (Langmead and Salzberg, 2012). After variant detection, all samples were genotyped for every variable position. The SNPs that were polymorphic, contained less than 10% missing data, with quality values higher than 40, supported by a minimum read depth of 10 and with a minor allele frequency (MAF) higher than 5% were retained for further analysis. Also, only samples that had less than 10% missing data were retained. One filtered VCF file was built for the wild accessions (134 wild accessions and 4,593 SNPs) and a second VCF file for wild and domesticated accessions (270 accessions and 4,779 SNPs). These VCF files were annotated using the GFF of common bean and converted to other formats, as needed, by using the tools implemented in the programs NGSEP (Duitama et al., 2014), Tassel v. 5.0 (Bradbury et al., 2007), and PGDSpider v. 2.0.8.2 (Lischer and Excoffier, 2012). All.*bam* files generated in this study for the 270 accessions were deposited in the SRA database of GenBank under accession number SRP115055.

### Genetic structure of wild lima bean

Several tools were applied to evaluate the genetic structure of wild Lima bean. First, a Nei's standard genetic distance matrix was built among individuals with the software GenAlex v. 6.5 (Peakall and Smouse, 2012). This genetic distance matrix was then used to build an unrooted neighbor-joining (NJ) tree with the software Phylip (Felsenstein, 1993) and to carry out a principal coordinate analysis (PCoA) in GenAlex v. 6.5 in order to explore the major grouping patterns in the dataset. Second, the Bayesian clustering approach implemented in the software Structure v. 2.3.4. (Pritchard et al., 2000) was applied. The model used was admixture with correlated allele frequencies. We evaluated values of K from 2 to 6 and run 10 independent simulations for each value of K. Each simulation consisted of a burnin period of 100,000 and 100,000 MCMC (Markov Chain Monte Carlo) steps after burnin. The software CLUMPP (Jakobsson and Rosenberg, 2007) was used to obtain a single Q matrix for each K and the software Structure Harvester (Earl and vonHoldt, 2012) was used to obtain the optimal K according to Evanno et al. (2005). For the optimal K, each individual was assigned to the K population from which it derived more than 70% of its ancestry; otherwise, the individuals were classified as admixed. For each wild cluster observed, several measures of genetic variability were calculated with the software GenAlex, namely, average number of alleles per locus (*N*_A_), average effective number of alleles per locus (*N*_E_), Shannon's diversity index (*I*), observed heterozygosity (*H*_O_), expected heterozygosity (*H*_E_), and fixation index (*F*).

### Geographic areas and number of domestication events in lima bean

**Figure 1:**
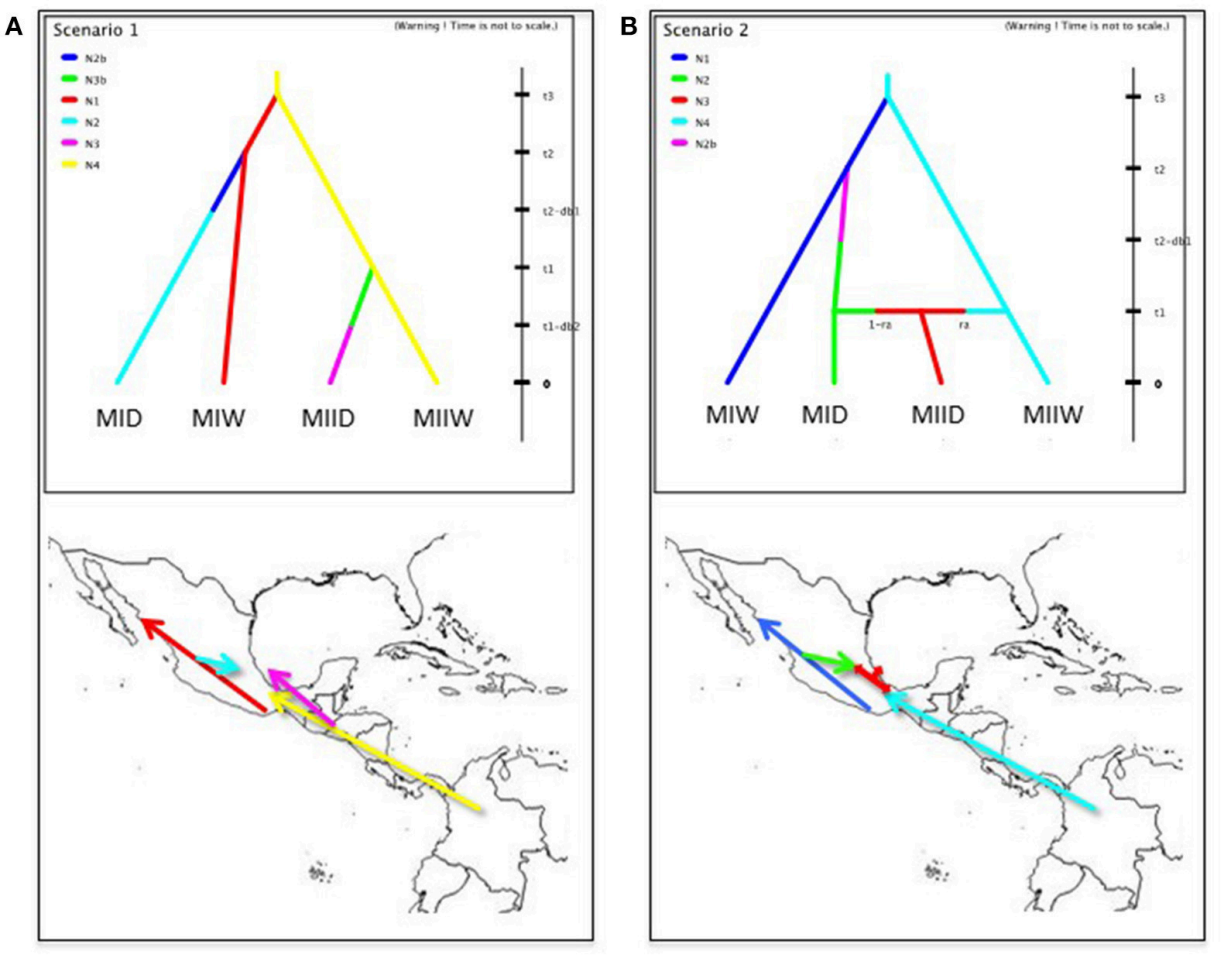
Domestication scenarios of Lima bean evaluated by an approximate Bayesian computation approach. In both scenarios, the two Mesoamerican wild populations (MIW and MIIW) diverged at time *t3*. The time of divergence of wild populations is much older than the divergence of domesticated populations (MID and MIID) from their wild sources (*t1* and *t2*). The origin of each domesticated population from its wild source population involved a domestication bottleneck (*db*) for a number of *t* generations and an effective size *Ni* smaller than the effective size of the source population. After the domestication bottleneck, the domesticated populations reached a larger and stable population size. All wild source populations are stable in size. **(A)** Scenario 1: Mesoamerican landraces (MID and MIID) come from two independent domestication events from MIW and MIIW, respectively. **(B)** Scenario 2: Mesoamerican landraces (MID) come from one domestication event from MIW. MIID landraces are the product of admixture between MID and MIIW, at an admixture rate of *ra*. Full explanation of parameters is found in Table 1.

To recover genetic clusters of wild and domesticated accessions, the same methods outlined above for wild Lima beans were used. The clustering patterns among wild and domesticated accessions may indicate how many times and where Lima beans were domesticated. Two competing domestication scenarios (scenarios 1 and 2) (see Figure 1) were evaluated by an approximate Bayesian computation (ABC) approach. In both scenarios, the two Mesoamerican wild populations (MI and MII) diverged at time *t3*. The time of divergence of wild populations is much older than the divergence of domesticated populations from their wild sources (*t1* and *t2*). The origin of each domesticated population from its wild source population involved a domestication bottleneck (*db*) for a number of *t* generations and an effective size *Ni* smaller than the effective size of the source population. After the domestication bottleneck, the domesticated populations reached a larger and stable population size. All wild source populations are stable in size.

For the ABC estimation of the posterior probabilities of the two domestication scenarios, the software DIYABC 2.1.0 was used (Cornuet et al., 2014). For this approach, a new SNP matrix (2,527 SNPs) was built to contain 30 accessions from wild gene pool MI, 30 accessions from wild gene pool MII, 30 accessions from domesticated gene pool MI and 17 accessions (the only accessions available) from domesticated gene pool MII, for a total of 107 accessions. These accessions are marked with an asterisk in their ID in Table S1. In selecting these accessions, care was taken to not include accessions classified as admixed in the Structure analysis because this analysis assesses the recent contribution of the different populations to the accessions (Cornille et al., 2012), and for the ABC analysis we want to assess historical contributions, not recent ones. In this ABC approach, a total of 200.000 genetic datasets were simulated under the coalescent model and each scenario was considered to be equally probable. In these simulations, the values of the parameters, which can be seen in Table 1, were drawn from their prior distribution and were kept as generic as possible. For Lima bean, one generation corresponds to 1 year. Conditions were set as *t3* > *t2* and *t3* > *t1*, according to previous studies (Serrano-Serrano et al., 2010). The duration of bottleneck (*db1* and *db2*) was set between one and three thousand generations because previous studies suggest that domestication may involve slow rates of evolution (Purugganan and Fuller, 2011). Time of domestication (*t1* and *t2*) was set between 2,000 and 10,000 generations (or years) according to archeological data for Lima bean (Kaplan and Lynch, 1999).

**Table 1:** Parameters used for ABC inferences and their prior distributions.

**Parameters**	**Description**	**Prior**
*N1*	Population size of wild gene pool MI	Uniform (10–2.000.000)
*N2*	Population size of domesticated gene pool MI	Uniform (10–2.000.000)
*N3*	Population size of domesticated gene pool MII	Uniform (10–2.000.000)
*N4*	Population size of wild gene pool MII	Uniform (10–2.000.000)
*N2b*	Population size of domesticated gene pool MI during domestication bottleneck	Uniform (10; 5.000)
*N3b*	Population size of domesticated gene pool MII during domestication bottleneck	Uniform (10; 5.000)
*t1*	Time of domestication within gene pool MII in generations	Uniform (2.000; 10.000)
*t2*	Time of domestication within gene pool MI in generations	Uniform (2.000; 10.000)
*t3*	Divergence time between wild gene pools MI and MII in generations	Uniform (300.000; 1.000.000)
*t1-db2*	Duration of domestication bottleneck in MII in generations	Uniform (1.000; 3.000)
*t2-db1*	Duration of domestication bottleneck in MI in generations	Uniform (1.000; 3.000)
*r_*a*_*	Admixture rate among domesticated MI and wild MII to give rise to domesticated MII	Uniform (0.001–0.999)

For each scenario, a pre-evaluation of model-prior combination was done in two ways. First, a principal component analysis was done at 10,000 simulated datasets to see how the observed data are located in relation to the space of simulated data. Second, simulated summary statistics are evaluated to count how often they are over-estimated or sub-estimated in relation to the observed data.

For each simulation, the summary statistics calculated were mean genetic diversity and mean genetic divergence measured by F_ST_ (and for scenario 2 an additional summary statistics was admixture rate, *r*_*a*_). The calculation of the posterior probability of each scenario is based on the distance or difference that exists between the summary statistics calculated for each simulated dataset and the observed dataset. The estimation of the posterior probability of scenarios was done by applying a direct approach and a weighted polychotomous logistic regression on 500 and 10,000 simulated datasets, respectively. In this regression the predictor variables were the differences between observed and simulated summary statistics.

The best domestication scenario was selected as the one with the highest posterior probability. We evaluated the confidence in choosing the best scenario by simulating 1,000 pseudo-observed data sets (pods). Then, posterior probabilities of the scenarios were calculated and finally type I and type II errors were estimated. To assess the fit of the best scenario in relation to the observed dataset, the model checking option implemented in DIYABC was used. This option evaluates the goodness of fit of model-posterior combinations. Finally, the posterior distributions of parameters under the best scenario were calculated by using the 1% simulated datasets that are closest to the observed dataset. The bias and precision in parameter estimation was calculated with 1,000 pods using the several dispersion measures implemented in DIYABC.

### Domestication founder effects

To investigate founder effects due to domestication on the Lima bean genome two analyses were conducted in a sample of 160 wild and 110 domesticated accessions. First, the percent reduction (*%r*) in expected heterozygosity among wild (*H*_*EW*_) and domesticated accessions (*H*_*ED*_) was calculated as follows: *%r* = (*H*_*EW*_-*H*_*ED*_)/(*H*_*EW*_). This reduction was measured in the whole sample and within the Mesoamerican and Andean gene pools. This reduction was also calculated locus by locus for the whole sample of accessions. Second, to compare the level of linkage disequilibrium (LD) by chromosome among wild and domesticated accessions, the full matrix option of the program Tassel was used. The method to detect regions with significant differences in LD among wild and domesticated accessions is described below.

### Outlier loci and genomic regions in high LD related to domestication

In order to identify outlier loci related to domestication, two analyses were carried out. First, loci that show significant divergence among wild and domesticated beans were detected by means of their F_ST_ values using the program Bayescan (Foll, 2012). In order to increase the number of SNP markers analyzed by Bayescan, a new SNP matrix was created with 95 wild and domesticated accessions with the highest coverage from the sequencing process. In this new matrix, with less missing data, we could retain 7,759 high quality SNPs. We used Bayescan to make two comparisons: (1) among all wild and all domesticated accessions and (2) among wild and domesticated accessions within the Mesoamerican and within the Andean gene pool. For Bayescan we used a burnin period of 50,000 followed by 100,000 iterations. To declare outlier loci we used a false-discovery rate of 0.05. Second, the island model proposed by Excoffier et al. (2009) and implemented in the software Arlequin (Excoffier and Lischer, 2010) was applied to identify outlier loci based on F_ST_ statistics in a set of 100 wild and domesticated accessions from gene pool MI (2,827 SNPs). For this, we simulated 50,000 datasets with two main groups (wild and domesticated), 100 demes within each group, and a maximum expected heterozygosity of 0.5. To declare outlier loci we took into account those loci that fell outside 99% confidence intervals of the null distribution.

Genomic regions with significant differences in LD between wild and domesticated beans were detected by applying the methods implemented in the software varLD (Ong and Teo, 2010). varLD aims to identify genomic regions with significant differences in LD patterns among two populations relative to the LD differences found in the rest of the genome (Teo et al., 2009). The significance of the varLD scores was obtained by resampling methods.

After identifying the outlier SNP loci based on F_ST_, these SNPs were structurally and functionally annotated by comparing the physical position of each SNP with the GFF annotation of the *P. vulgaris* genome reported by Schmutz et al. (2014). This annotation allowed to see whether the SNPs had non-synonymous effects on coding sequences. Because the genomic regions with different LD patterns among wild and domesticated accessions may contain genes related to domestication, we counted within the LD regions the number of genes that displayed domestication signatures in *P. vulgaris*, according to the results reported by Schmutz et al. (2014), and then we evaluated whether the function of these genes was or not related to domestication.

## Results

### SNP detection by GBS

The GBS technique produced a total of 565,386,739 sequence reads for the 270 accessions analyzed. Of these, 242,489,038 reads were aligned at unique positions in the reference genome, 112,212,070 reads were aligned at multiple positions and 210,685,631 reads were not aligned. The percentage of reads aligned was 63%. In average, every sample produced 2,094,025 reads of which 898,108 aligned to unique positions. The reads aligned at unique positions were further used for variant detection among the samples analyzed. This process produced an unfiltered VCF file containing 895,110 biallelic SNPs and 11,265 biallelic indels. A total of 322,052 biallelic SNPs were located in coding regions, 162,892 of them were synonymous substitutions, 157,279 were missense substitutions, and 1,881 were non-sense substitutions. After filtering, a total of 4,779 biallelic SNPs were retained, 3,439 of them located in coding regions, 2,245 were synonymous substitutions, 1,192 missense substitutions, and two non-sense substitutions. Therefore, synonymous substitutions were twice more frequent than non-synonymous substitutions in the filtered dataset. Transitions were also more frequent than transversions (transition/transversion ratio = 1.32). Raw read number, mapped reads, and number of SNPs genotyped per accession can be seen in Table S2. The distribution of the SNPs by chromosome can be seen in Figure S2. Given a genome size of common bean of about 587 MB (Schmutz et al., 2014), the map density would be of one SNP every 123 Kb.

### Genetic structure of wild lima bean

**Figure 2:**
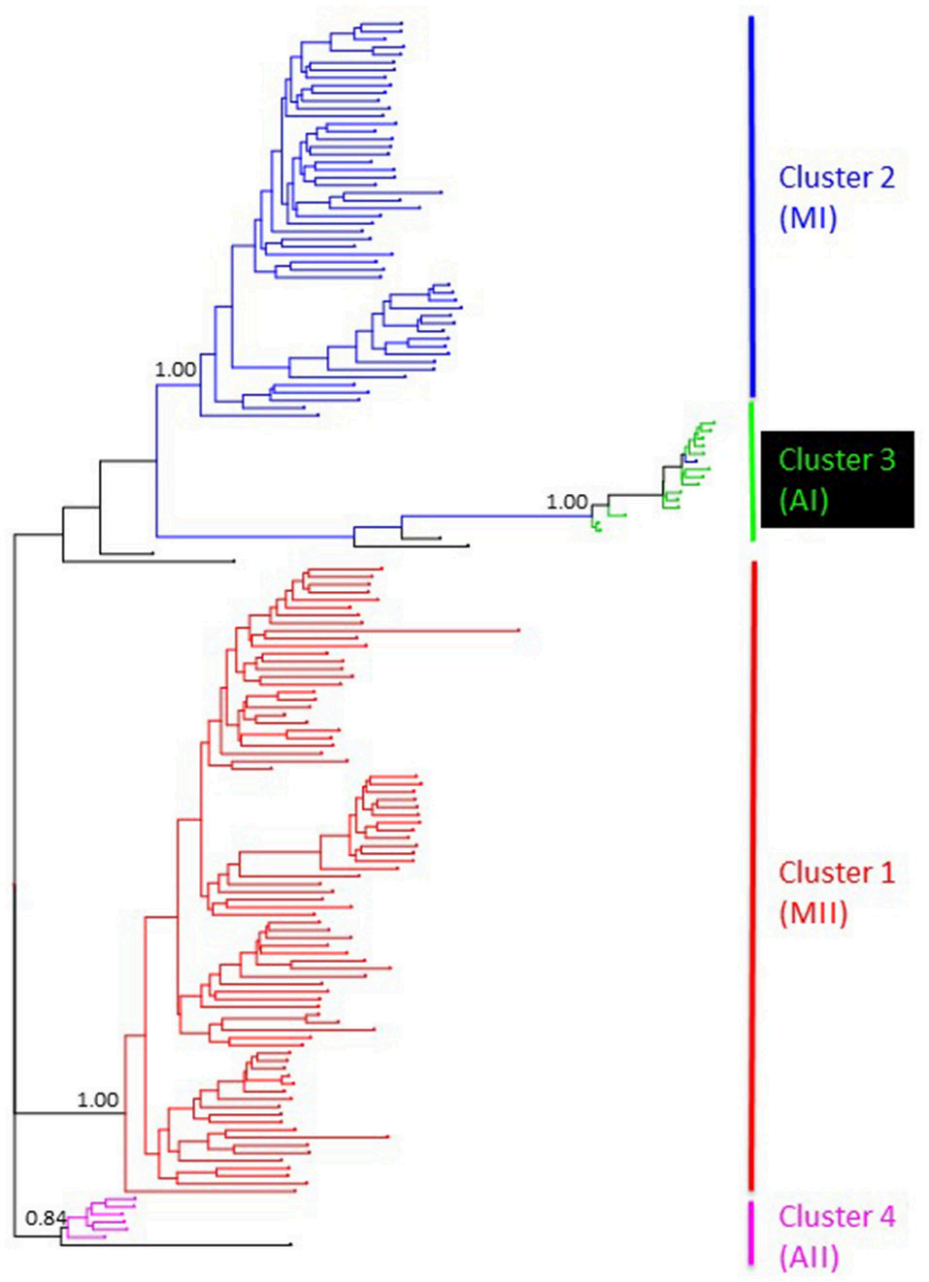
NJ topology showing the genetic relationships among the 134 wild Lima bean accessions included in this study on the basis of 4,593 SNPs detected by GBS. Names on the right side indicate the clusters detected. Numbers on nodes indicate bootstrap support.

**Figure 3:**
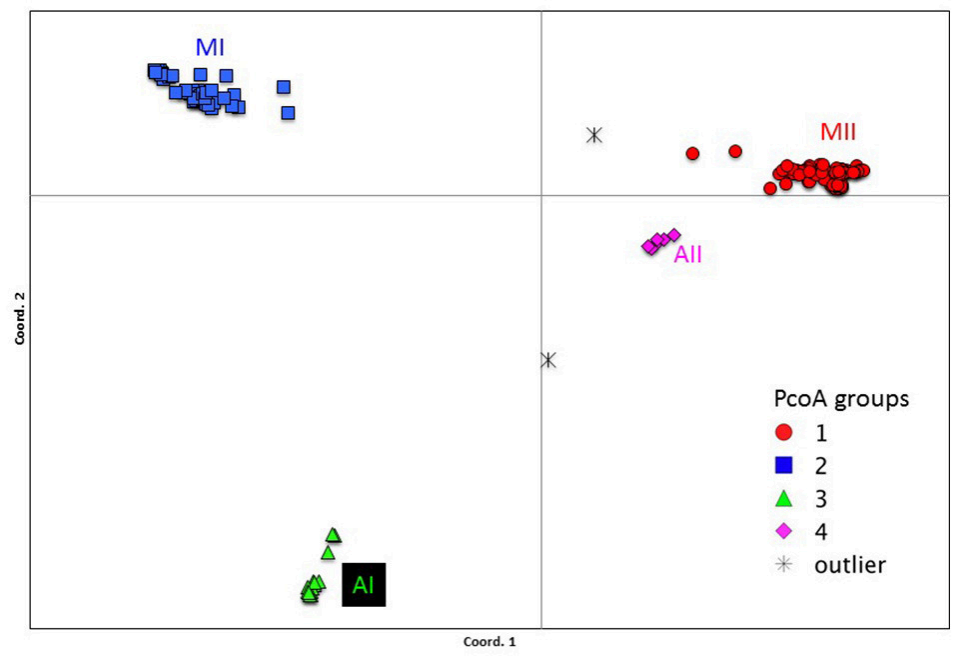
Plot showing the first two coordinates of a principal coordinate analysis carried out on 134 wild Lima bean accessions on the basis of 4,593 SNPs detected by GBS. The color coding shows the grouping of accessions according to the NJ analysis (Figure 2).

**Figure 4:**
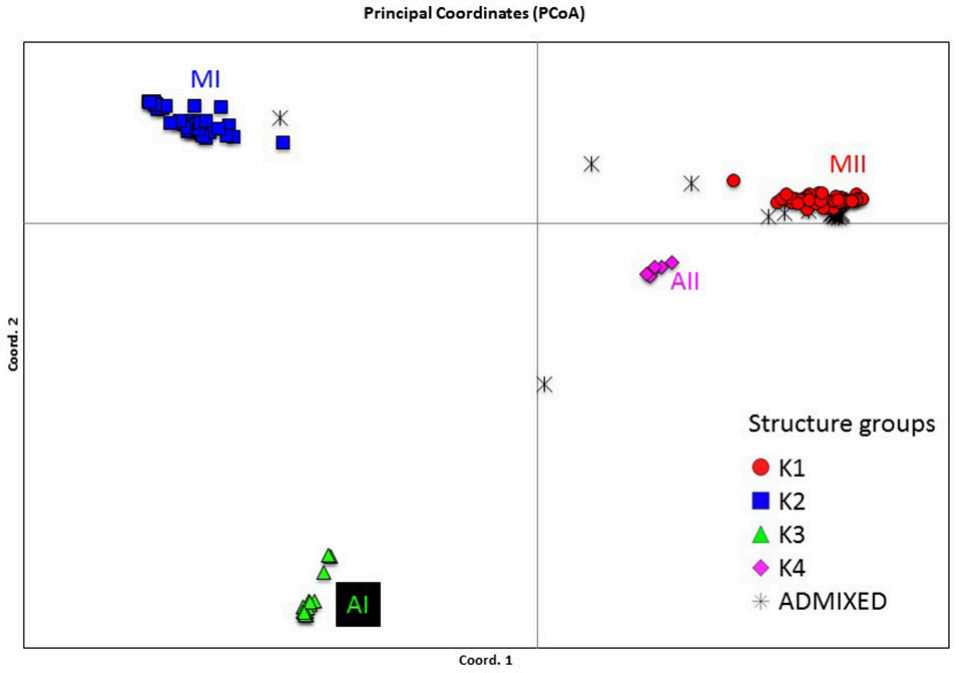
Plot showing the first two coordinates of a principal coordinate analysis carried out on 134 wild Lima bean accessions on the basis of 4,593 SNPs detected by GBS. The assignment of accessions was according to the results of the Structure analysis.

Genetic structure of the wild Lima bean was explored in a set of 134 wild accessions and a total of 4,593 SNPs by means of a NJ analysis, a PCoA and assignment tests implemented in the software Structure. The results of these analyses can be seen in Figures 2, 3, 4. The results of Structure indicated that the optimum K was 5. Accessions could be assigned to four of these populations (K1 to K4) and the rest of accessions (20 in total, 15%) were classified as admixed. In general, there was a high concordance in the clustering patterns obtained with the three kinds of analyses (except for the set of 20 admixed accessions), therefore 114 wild accessions could be assigned to four different clusters or gene pools (MI, MII, AI, and AII).

In Table S1, the PcoA and Structure clusters assigned to each accession can be seen. Below there is a short description for each one of the clusters. Discrepancies between our current classification of accessions in gene pools based on GBS data and previous studies based on ITS data can be seen in Table S3.

#### Cluster 1

**Figure 5:**
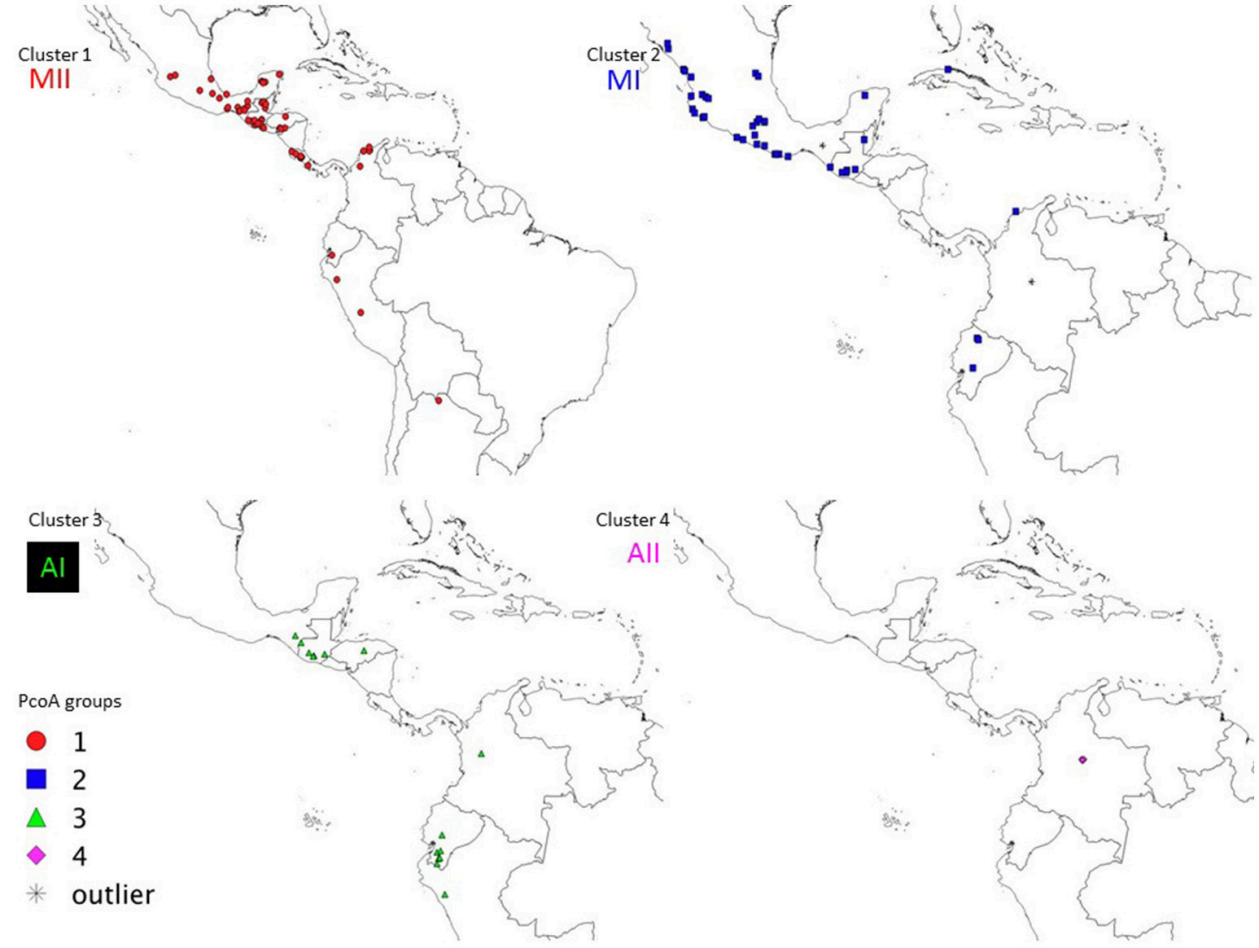
Geographic distribution of the four wild clusters (MI, MII, AI, and AII) observed in the PcoA for 134 wild accessions of Lima bean detected on the basis of 4,593 SNPs.

This cluster contains 48 accessions mainly distributed in Mexico (humid coastal plains of the Gulf of Mexico in the states of Veracruz and Tamaulipas, Chiapas, Oaxaca and the Peninsula of Yucatán), in Central America (Guatemala, Honduras and Costa Rica), northern Colombia, Ecuador (Azuay), Peru (Cajamarca and Junín), and Argentina (Salta) (Figure 5). This is the most widely distributed cluster and corresponds to the Mesoamerican gene pool MII of previous studies (Serrano-Serrano et al., 2010, 2012), therefore we will call this cluster MII hereafter. A total of 29 accessions in this cluster have been evaluated for seed size at CIAT. Seed size varies from 4 to 15.3 g/100 seeds, with average of 8.8 g/100 seeds, a seed size that is within the range of Mesoamerican wild Lima beans.

#### Cluster 2

This cluster contains 45 accessions mainly distributed in Mexico (36 accessions) along the western and southern Pacific coastal plains and hills in the states of Sinaloa, Nayarit, Jalisco, Colima, Michoacán, Guerrero, and Oaxaca, the Gulf of Mexico dry coastal plains in the state of Tamaulipas, one accession in Yucatán, two accessions in Puebla and two in Morelos, some few accessions in Central America (Guatemala and Belize), one accession in Cuba, one in northern Colombia (Atlántico department) and three in Ecuador (Chimborazo, Imbabura and Pichincha) (Figure 5). This is the second most widely distributed gene pool and its geographic distribution is within the range of the Mesoamerican gene pool MI reported in previous studies, except for the three accessions from Ecuador (G26469, G26606, G26751A). We will call this cluster MI hereafter. A total of 21 accessions have seed size data reported by CIAT. Seed size ranges from 5 to 25.4 g/100 seeds, with an average of 10.9 g/100 seeds, a size that is within the range of Mesoamerican wild Lima beans.

#### Cluster 3

This cluster contains 15 accessions from South America (six accessions from Ecuador, one from Colombia, and one from Peru), Guatemala (five accessions: G25844, G26653, G26655, G26684, G26732), one accession from Honduras (G26630), and one from Mexico (Chiapas, G26753) (Figure 5). All the accessions have seed size data taken by CIAT and show an average of 13.6 g/100 seeds. The geographic distribution of these accessions in Ecuador and Peru corresponds to the range of the Andean gene pool observed in previous studies, and the close genetic relationship of these Andean accessions with accessions from Central America has not been reported before. We will call this cluster Andean I (AI) hereafter.

#### Cluster 4

Only six accessions, all of them from the central departments of Boyacá and Cundinamarca in Colombia, belong to this cluster. Seed size in this cluster ranges from 12.2 to 17.4 g/100 seeds, with an average of 15 g/100 seeds, a range characteristic of Andean wild Lima beans. We will call this cluster AII hereafter.

Table 2 summarizes genetic diversity values for the four wild gene pools or clusters. As can be seen, there was practically no observed heterozygote genotypes in the SNPs analyzed, or very few, as expected for an autogamous species. Among the four wild gene pools, the MII gene pool (H_E_ = 0.138) was slightly more diverse than the MI gene pool (H_E_ = 0.115), and gene pools AI and AII were four or five times less diverse, although this may be due to the fact that the sampling scheme in this study was more focused toward the Mesoamerican wild gene pool.

**Table 2:** Diversity indexes for wild and domesticated accessions of Lima bean and for the four gene pools observed in this study, calculated on the basis of 4,779 SNP markers.

**Population**		**N**	***P***	***N*_A_**	***N*_E_**	***I***	***H*_O_**	***H*_E_**	***F***	**Global reduction in H_E_ in landraces *%r***	**Locus-by—locus reduction in H_E_ in landraces *%r***	**Locus-by—locus increase in H_E_ in landraces**
All Wild	Mean	153.2	99.96	2.000	1.438	0.438	0.000	0.278	0.998	18.34%	44.0% (66% of loci)	31% (33% of loci)
	SE	0.051		0.000	0.004	0.002	0.000	0.002	0.000			
All Dom.	Mean	106.1	99.23	1.992	1.343	0.370	0.000	0.227	0.999			
	SE	0.034		0.001	0.004	0.003	0.000	0.002	0.000			
Wild MI	Mean	43.9	71.42	1.419	1.191	0.178	0.000	0.115	1.000	31.30%	78.0% (84.6% of loci)	34% (15% of loci)
	SE	0.018		0.007	0.005	0.004	0.000	0.003	0.000			
Dom. MI	Mean	72.2	45.80	1.458	1.125	0.131	0.000	0.079	0.997			
	SE	0.024		0.007	0.004	0.003	0.000	0.002	0.001			
Wild MII	Mean	46.8	64.53	1.540	1.225	0.217	0.000	0.138	1.000	0	53.0% (42.3% of loci)	33% (54.5% of loci)
	SE	0.018		0.007	0.005	0.004	0.000	0.003	0.000			
Dom. MII	Mean	16.4	54.24	1.542	1.267	0.254	0.000	0.164	0.998			
	SE	0.012		0.007	0.005	0.004	0.000	0.003	0.000			
Wild AI	Mean	14.7	9.23	1.086	1.048	0.044	0.000	0.029	1.000	0	47.0% (79% f loci)	16% (21% of loci)
	SE	0.010		0.004	0.003	0.002	0.000	0.001	0.000			
Dom. AI	Mean	10.5	10.0	1.100	1.047	0.046	0.000	0.030	0.977			
	SE	0.012		0.004	0.002	0.002	0.000	0.001	0.002			
Wild AII	Mean	5.9	9.71	1.096	1.059	0.053	0.000	0.036	1.000	–	–	–
	SE	0.006		0.004	0.003	0.002	0.000	0.002	0.000			
Total	Mean	129.6		1.996	1.391	0.404	0.000	0.252	0.999	–	–	–
	SE	0.243		0.001	0.003	0.002	0.000	0.001	0.000	–	–	–

*N, Average sample size; P, Percent of polymorphic loci; N_A_, Average number of alleles per locus; N_E_, Average effective number of alleles per locus; I, Shannon's diversity index; H_O_, Observed heterozygosity; H_E_, Expected heterozygosity; F, Fixation index; %r, Reduction in genetic diversity due to domestication (or founder effect) calculated as (H_EW_-H_ED_)/(H_EW_) where H_EW_ is genetic diversity in wild accessions and H_ED_ is genetic diversity in domesticated accessions*.

### Domestication patterns in lima bean

#### Indirect methods

**Figure 6:**
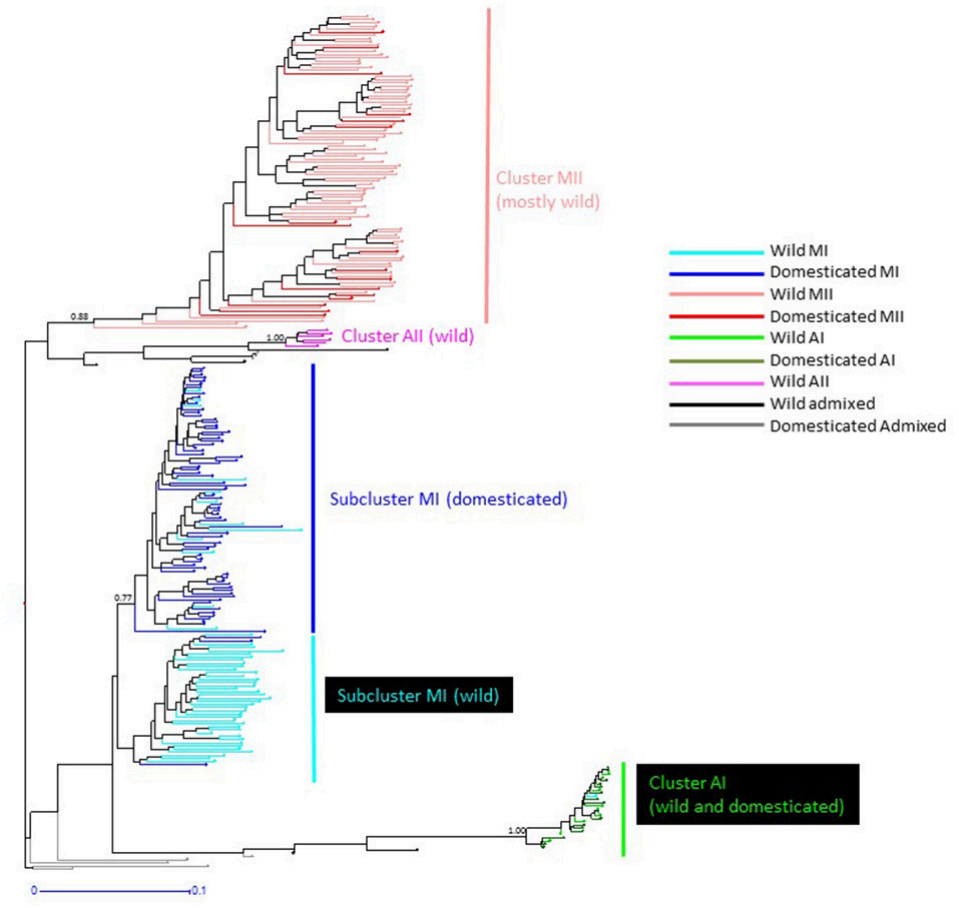
NJ topology showing the genetic relationships among the 270 wild and domesticated Lima bean accessions included in this study on the basis of 4,779 SNPs detected by GBS. Names on the right side indicate the clusters detected. Names on nodes indicate bootstrap support. Within cluster MI, wild accessions are shown as light blue lines and domesticated accessions as dark blue lines. Within cluster MII, wild accessions are shown as light red lines and domesticated accessions as dark red lines. Within cluster AI, wild accessions are shown as bright green lines and domesticated accessions as dark green lines. Wild admixed accessions are shown as black lines and domesticated admixed accessions as gray lines.

**Figure 7:**
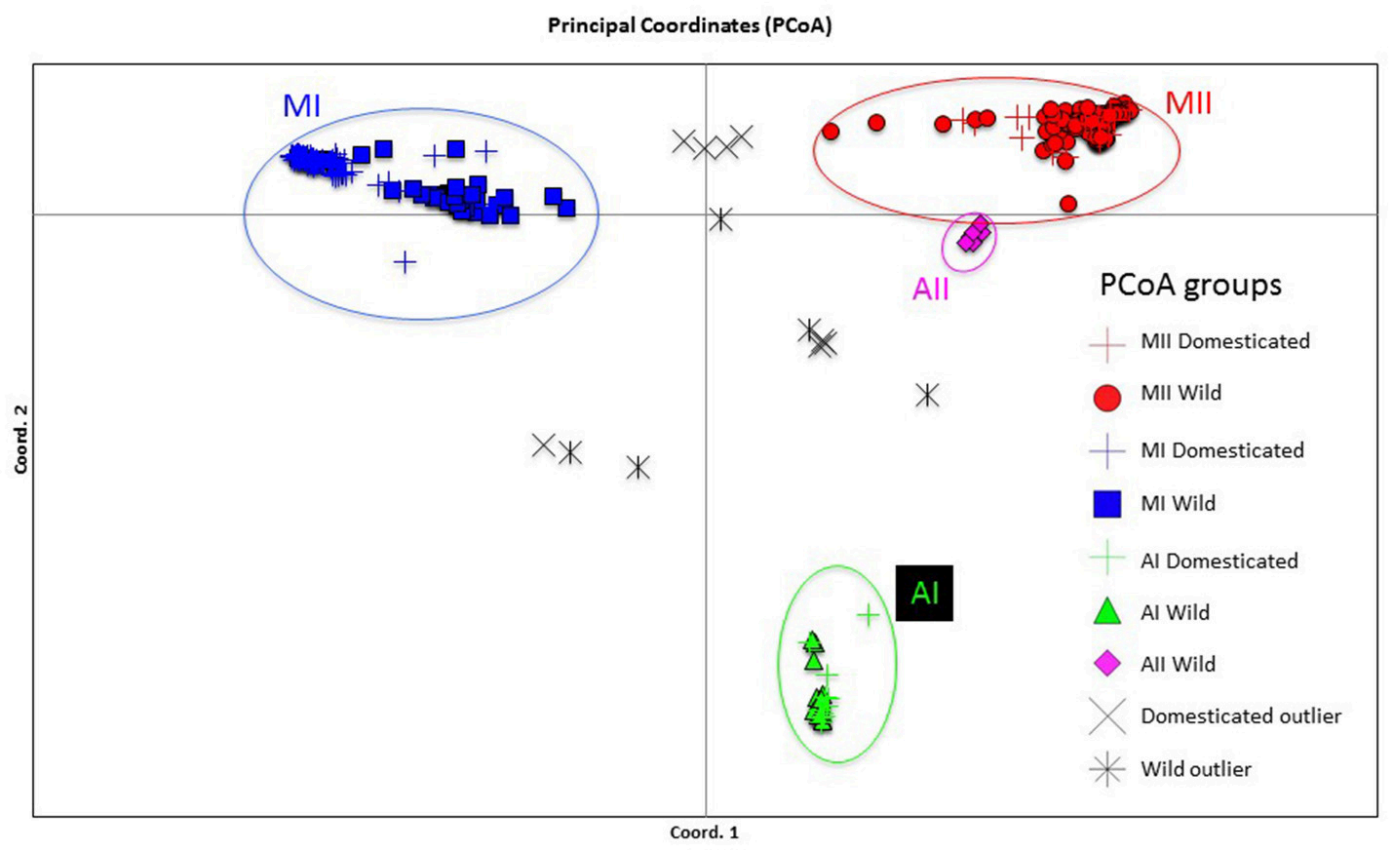
Plot showing the first two coordinates of a principal coordinate analysis carried out on 270 wild and domesticated Lima bean accessions on the basis of 4,779 SNPs detected by GBS. The color coding shows the grouping of accessions according to the NJ analysis (Figure 6).

**Figure 8:**
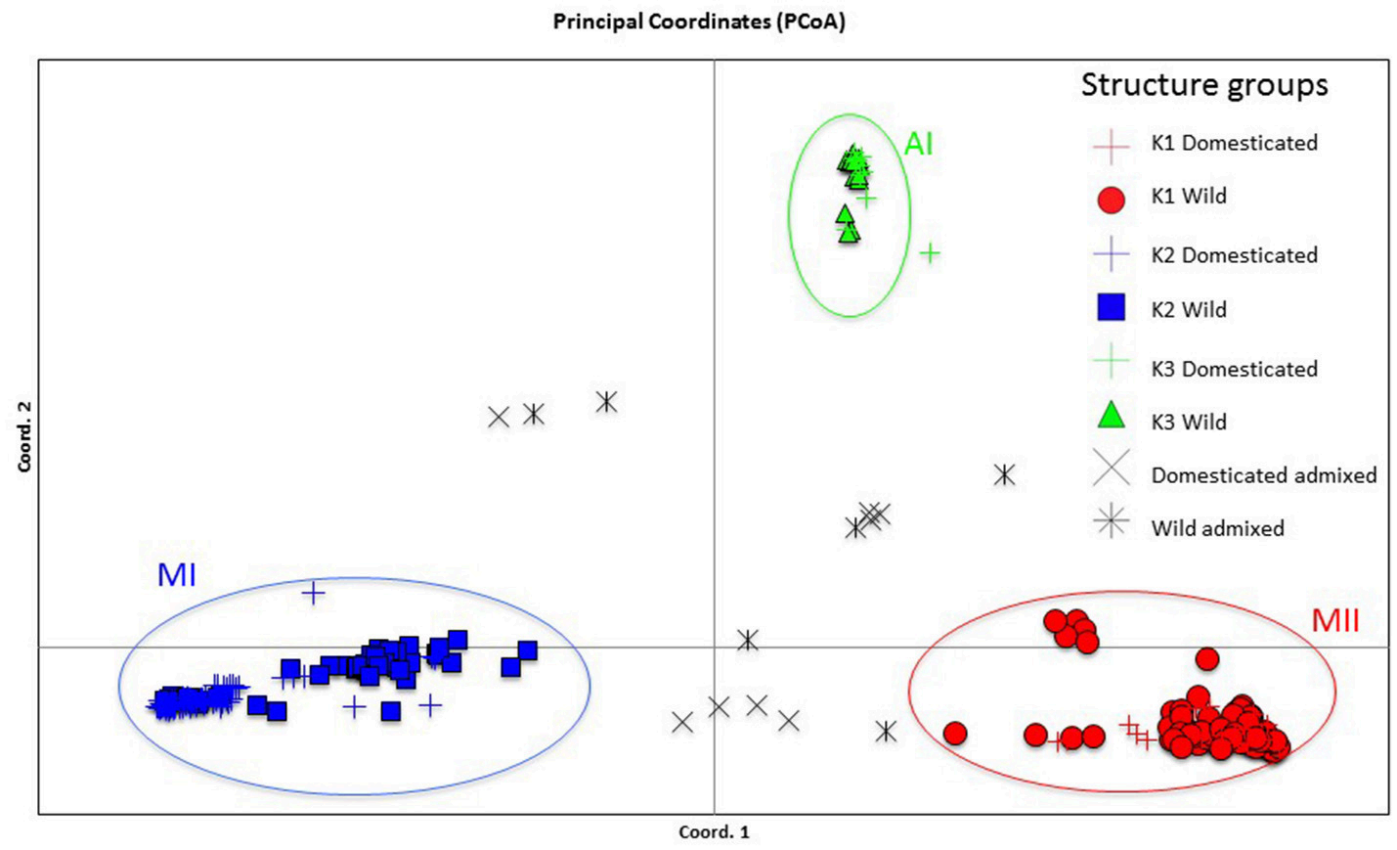
Plot showing the first two coordinates of a principal coordinate analysis carried out on 270 wild and domesticated Lima bean accessions on the basis of 4,779 SNPs detected by GBS and the assignment of accessions according to the results of the Structure analysis.

**Figure 9:**
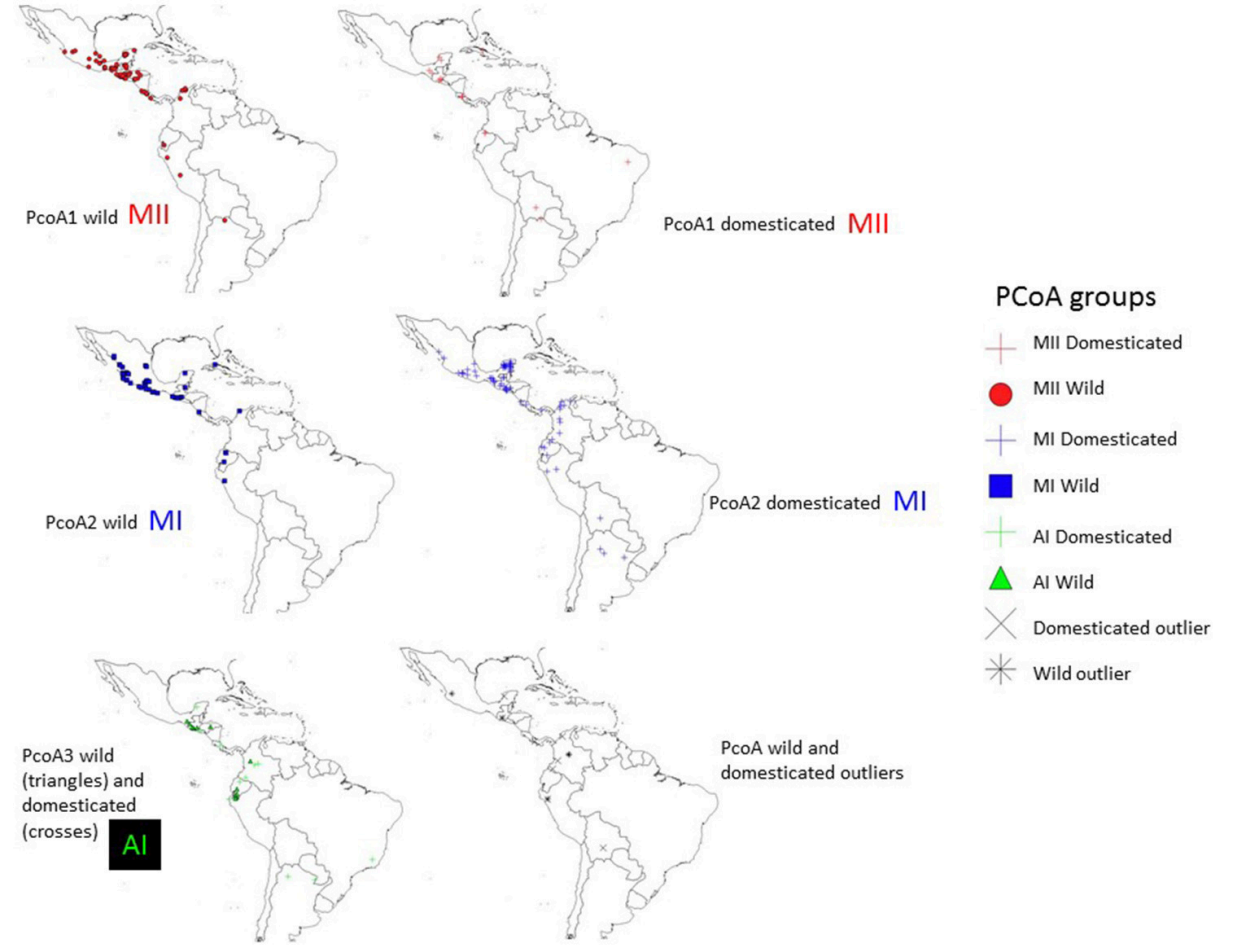
Geographic distribution of clusters MI, MII and AI and outliers observed in the PcoA for 270 wild and domesticated accessions of Lima bean detected on the basis of 4,479 SNPs.

The genetic relationships among 160 wild and 110 domesticated accessions were studied by means of genetic distances, unrooted NJ topologies (Figure 6), PCoA (Figure 7), and Bayesian approaches with the software Structure (Figure 8). These analyses may tell us how many times and where Lima beans were domesticated. The first axis of the PcoA explained 28.40% of variation, the second one explained 15.54%, and the third one explained 3.15%, for a cumulative of 47.09%. It can be seen in Figures 6–8 that domesticated accessions grouped along with wild accessions within clusters MI, MII, and AI. The Structure analysis indicated that the optimum K was three: one for wild and domesticated accessions within gene pool MI (population K2), another one for wild and domesticated accessions within gene pool MII (population K1) and a third one for wild and domesticated accessions within gene pool AI (population K3). The Structure analysis reported a total of 14 admixed accessions: 8 domesticated and 6 wild. Figure S3 shows the global ancestry derived from gene pools AI, MI, and MII for these 14 admixed accessions. The geographic distribution of wild and domesticated accessions within MI, MII and AI clusters and the admixed or outlier accessions can be seen in Figure 9.

In the cluster MI, most of the domesticated accessions (74 accessions, 67%), with an average seed size of 48 g/100 seeds, grouped together with 53 wild accessions. In this cluster we found three domesticated accessions from Bolivia, Ecuador, and Peru (G25981, G26480, G25909, respectively) that show larger seed sizes (from 64 to 121 g/100 seeds) typical of Andean beans.

In the cluster MII, only 15% of the domesticated accessions (17 accessions), with an average seed size of 53 g/100 seeds, grouped together with 82 wild accessions. In this cluster we found three accessions, one from Bolivia, and two from Ecuador (G27337, G26659, G26672, respectively) that show larger seed sizes (from 75 to 115 g/100 seeds) typical of Andean beans.

In the cluster AI, 10% of the domesticated accessions (11 accessions) with an average seed size of 66 g/100 seeds grouped together with 14 wild accessions. In this cluster we found four accessions (G26290, G26438, G25277, G25771) from Argentina, Costa Rica, El Salvador, and Mexico, respectively, that show smaller seed sizes (from 40 to 53 g/100 seeds), typical of Mesoamerican landraces. These accessions were classified as Mesoamerican in a previous study based on ITS polymorphisms (Serrano-Serrano et al., 2012). The sampling in this study was more focused on wild and domesticated accessions from Mesoamerica, for this reason we will describe below in more detail the clustering pattern of MI and MII.

It can be seen in Figure 6 that within MI cluster, domesticated accessions and wild accessions are grouped within separate subclusters. Within the domesticated subcluster however, 13 wild accessions were found. These wild accessions come from Mexico (four accessions in Morelos, Oaxaca and Yucatán), Guatemala (four accessions), Cuba (one weedy accession), Colombia (one accession), and Ecuador (three accessions). The inverse is also true, within the wild subcluster two domesticated accessions were found, one from Panama and another one from Peru. The significance of this is not clear but may indicate cases of introgression, at least for the wild sample in Yucatán where cases of introgression among wild populations and landraces have been documented (Martínez-Castillo et al., 2007; Dzul-Tejero et al., 2014). Also, in the PCoA plot of axis 1 vs. 3, we can see that most of the MI domesticated accessions tend to cluster together and apart from the MI wild accessions on axis 3 (see Figure S4).

#### ABC approach

We compared two domestication scenarios with an ABC approach (Figure 1) to evaluate the hypotheses of single or multiple domestications of landraces in Mesoamerica. Before estimating posterior probabilities of both scenarios, we evaluated model-prior combinations (see Table S4 and Figure S5). The PCA plots show how observed data are within the cloud of simulated data for both scenarios, indicating a good model-prior combination. Table S4 shows that for most of the summary statistics analyzed, simulated data are not significantly different from observed data.

The best-supported domestication scenario with the direct approach was scenario 1 (although scenario 2 also got some support) and with the logistic approach was scenario 2 (and here scenario 1 did not get any support) (see Table 3 and Table S5), indicating than both scenarios may be supported by the genetic data.

**Table 3:** Statistics used to choose among the two competing domestication scenarios and obtained on the basis of an ABC approach.

**Scenario**	**Direct approach**	**Type I error^b^**	**Type II error^c^**	**Logistic approach**	**Type I error^e^**	**Type II error^f^**
	**Posterior probability^a^**			**Posterior probability^d^**		
1. *Independent domestications within MI and MII in Mesoamerica*	0.7200 [0.3264,1.0000]	0.000	0.0065	0.0000 [0.0000,0.0001]	0.001	0.000
2. *A single domestication in Mesoamerica with admixture*	0.2800 [0.0000,0.6736]	0.057	0.000	1.0000 [0.9999,1.0000]	0.001	0.000

a *The estimation of the posterior probability of scenarios was done by applying a direct approach on 500 simulated datasets. The posterior probability is shown along with 95% confidence intervals*.

b *Type I error was estimated from datasets simulated under the true scenario as the proportion of datasets where the true scenario did not show the highest probability among the competing scenarios, probabilities estimated with direct approach*.

c *Type II errors were estimated from datasets simulated under the non-true scenario as the proportion of datasets where the true scenario showed the highest probability among the competing scenarios, probabilities estimated with direct approach*.

d *The estimation of the posterior probability of scenarios was done by applying a weighted polychotomous logistic regression on 10,000 simulated datasets. The posterior probability is shown along with 95% confidence intervals*.

e *Type I error was estimated from datasets simulated under the true scenario as the proportion of datasets where the true scenario did not show the highest probability among the competing scenarios, probabilities estimated with logistic regression*.

f *Type II errors were estimated from datasets simulated under the non-true scenario as the proportion of datasets where the true scenario showed the highest probability among the competing scenarios, probabilities estimated with logistic regression*.

The goodness of fit of model-posterior combination was done by means of a PCA using the model checking option of DIYABC. Figure S6 shows that observed data is within the cloud of simulated data based on posterior distributions, indicating a good fit of model-posterior combination for scenarios 1 and 2.

Parameter estimation was done on the basis of scenarios 1 and 2 separately and not by averaging because parameter values show large differences between the two scenarios (see Table 4 and Figures S7, S8). The performance of the method for parameter estimation was assessed by means of several bias and error measures (see Table S6).

**Table 4:** Parameter estimation of the ABC approach based on the posterior distribution of scenarios 1 and 2.

**Parameter^a^**	**Mean**	**Median**	**Mode**	**q025**	**q050**	**q250**	**q750**	**q950**	**q975**	**95% interval**
**SCENARIO 1**										
N1 (wild MI)	1.23E+05	1.26E+05	1.28E+05	6.10E+04	6.71E+04	1.03E+05	1.45E+05	1.68E+05	1.75E+05	1.14E+05
N2 (Dom MI)	6.78E+05	5.49E+05	7.40E+04	1.86E+04	3.91E+04	2.24E+05	1.04E+06	1.75E+06	1.86E+06	1.84E+06
N3 (Dom MII)	1.36E+06	1.49E+06	1.86E+06	2.00E+05	3.03E+05	1.01E+06	1.79E+06	1.97E+06	1.98E+06	1.78E+06
N4 (wild MII)	2.01E+05	2.03E+05	2.12E+05	9.28E+04	1.04E+05	1.64E+05	2.38E+05	2.92E+05	3.12E+05	2.19E+05
t1 (Dom MII)	8.30E+03	8.92E+03	9.53E+03	3.09E+03	3.84E+03	7.66E+03	9.55E+03	9.84E+03	9.89E+03	6.80E+03
db2 (Dom MII)	1.13E+03	1.07E+03	1.01E+03	1.00E+03	1.00E+03	1.03E+03	1.15E+03	1.38E+03	1.60E+03	6.00E+02
N3b (Dom MII)	4.61E+03	4.68E+03	4.79E+03	3.92E+03	4.02E+03	4.40E+03	4.85E+03	4.97E+03	4.98E+03	1.06E+03
t2 (Dom MI)	9.70E+03	9.86E+03	9.99E+03	8.29E+03	8.92E+03	9.67E+03	9.95E+03	9.99E+03	1.00E+04	1.71E+03
db1 (Dom MI)	2.73E+03	2.87E+03	2.98E+03	1.59E+03	1.97E+03	2.65E+03	2.95E+03	2.99E+03	3.00E+03	1.41E+03
N2b (Dom MI)	3.09E+02	2.21E+02	1.58E+02	6.18E+01	7.21E+01	1.43E+02	3.25E+02	7.69E+02	1.38E+03	1.32E+03
t3 (wild MI-MII)	3.67E+05	3.29E+05	3.06E+05	3.02E+05	3.03E+05	3.13E+05	3.77E+05	5.64E+05	6.76E+05	3.74E+05
**SCENARIO 2**										
N1 (wild MI)	2.04E+05	2.08E+05	2.29E+05	9.71E+04	1.08E+05	1.69E+05	2.42E+05	2.80E+05	3.00E+05	2.03E+05
N2 (Dom MI)	1.37E+06	1.51E+06	1.96E+06	1.50E+05	2.94E+05	9.87E+05	1.82E+06	1.96E+06	1.98E+06	1.83E+06
N3 (Dom MII)	5.92E+05	4.08E+05	7.79E+04	4.92E+04	6.46E+04	1.87E+05	8.95E+05	1.69E+06	1.82E+06	1.77E+06
N4 (wild MII)	3.46E+05	3.44E+05	3.64E+05	1.53E+05	1.76E+05	2.79E+05	4.04E+05	5.12E+05	5.72E+05	4.19E+05
t1 (Dom MII)	6.54E+03	6.95E+03	8.35E+03	2.36E+03	2.61E+03	4.90E+03	8.33E+03	9.27E+03	9.46E+03	7.10E+03
ra (MI Dom-MII W)	9.53E-01	9.53E-01	9.53E-01	9.25E-01	9.30E-01	9.44E-01	9.64E-01	9.80E-01	9.87E-01	6.20E-02
t2 (Dom MI)	7.67E+03	8.02E+03	9.20E+03	3.75E+03	4.44E+03	6.50E+03	9.15E+03	9.81E+03	9.90E+03	6.15E+03
db1 (Dom MI)	2.87E+03	2.95E+03	2.99E+03	2.10E+03	2.41E+03	2.87E+03	2.98E+03	3.00E+03	3.00E+03	9.00E+02
N2b (Dom MI)	4.98E+02	3.51E+02	2.28E+02	8.98E+01	1.15E+02	2.28E+02	5.49E+02	1.45E+03	1.98E+03	1.89E+03
t3 (wild MI-MII)	5.81E+05	5.51E+05	4.41E+05	3.15E+05	3.28E+05	4.33E+05	7.03E+05	9.24E+05	9.72E+05	6.57E+05

a *See Table 1 for an explanation of parameters*.

### Founder events

Table 2 shows the global percent reduction (*%r*) in genetic diversity in domesticated accessions compared to their wild ancestors (founder effect). Domesticated accessions compared to wild accessions (for the whole sample and within gene pool MI) are less diverse in terms of *P, N*_E_, *I* and *H*_E_. *P* was 25% higher in MI wild gene pool compared to MI domesticated gene pool, and 10% higher in MII wild gene pool compared to MII domesticated gene pool. *P* among wild and domesticated accessions within gene pool AI was very similar.

The founder effect when all domesticated accessions were compared to all wild accessions was around 18%. When this effect was measured within gene pools, contrasting values were obtained. While the founder effect within the MI gene pool was around 31%, there was no evidence of founder effect within gene pools MII and AI.

*%r* was also calculated locus-by-locus in the whole sample. Two-third parts of the loci, namely 3,167 loci, reported positive values for *%r* ranging from 1% to 100% and mean value of 44%. Positive values of *%r* indicate a founder effect, namely domesticated accessions contain less diversity than wild accessions. Also, about one-third of the loci, namely 1,562 loci, reported negative values for *%r* indicating that domesticated accessions in these loci contain more genetic diversity than wild accessions (in average 31% more diverse). Lastly, 48 loci reported values of cero, with no evidence of reduction in genetic diversity.

The fact that some loci are genetically more diverse in domesticated populations than in wild populations when the whole sample is analyzed may be a result of population structure within the domesticated gene pool. For this reason, *%r* was measured locus-by-locus within each gene pool (MI, MII, and AI) (see Table 2). Within gene pool MI, most loci (around 84.6%) showed positive values (in average a reduction of 78% in genetic diversity in these loci in landraces), 15% of loci showed negative values (in average 34% more diversity in these loci in landraces), and only 0.4% of loci showed no reduction in genetic diversity. Within gene pool MII, the results were quite different because most loci (around 54.5%) showed negative values (in average 33% more diversity in landraces in these loci), 42.3% of loci showed positive values (in average a reduction of 53% in genetic diversity in landraces) and 3.2% of loci showed no reduction in genetic diversity. In the Andean gene pool (AI), 79% of loci showed reduction in genetic diversity in the landraces (an average reduction of 47%), and 21% of loci showed increase in genetic diversity in the landraces (in average these loci contain 16% more genetic diversity).

To compare the LD among wild and domesticated accessions, chromosome-wise LD values were calculated as the correlation coefficient *r*^2^. It can be seen in Table 5 that in all the 11 chromosomes, average values of *r*^2^ are larger in domesticated accessions than in wild accessions (an increase of about 20–30%), possibly as a consequence of the reduction in genetic diversity during the domestication process and selection processes. In the section below, we identified particular chromosome regions with large differences in LD among wild and domesticated accessions that may indicate regions under selection.

**Table 5:** Average values of linkage disequilibrium measured as the correlation coefficient *r*^2^ by chromosome in the sample of wild and domesticated accessions.

**Chromosome**	**Wild accessions**	**Domesticated accessions**
1	0.13	0.19
2	0.13	0.18
3	0.12	0.17
4	0.12	0.16
5	0.12	0.17
6	0.12	0.15
7	0.14	0.18
8	0.12	0.15
9	0.12	0.17
10	0.12	0.15
11	0.13	0.16

### Outlier loci and genomic regions related to domestication

F_ST_ outlier loci related to domestication were detected by means of a Bayesian approach implemented in Bayescan and by the hierarchical method of Excoffier et al. (2009) implemented in Arlequin (Excoffier and Lischer, 2010). Figure S9 shows the five outlier loci detected by Bayescan in chromosomes one, three, six and nine. Table S7 shows the location and annotation of these five outlier loci. Of these five SNPs, the ones in chromosomes three and six lie within genes that according to their GO ontology are related to functions relevant for the domestication process (photoperiodism and seed germination, respectively). The SNP in chromosome 3 is interesting because it involves a missense change and the two SNPs in chromosome 9 fall within genes that were reported as Mesoamerican domestication genes in common bean by Schmutz et al. (2014). A total of 21 outlier loci were detected with the hierarchical method implemented in Arlequin (Excoffier and Lischer, 2010). Table S8 shows the location and annotation of these 21 loci; three of these SNPs involve missense changes and four of them have been reported as domestication genes in common bean (Phvul.001G024800, Phvul.002G262700, Phvul.003G111900, and Phvul.006G102400) by Schmutz et al. (2014).

**Figure 10:**
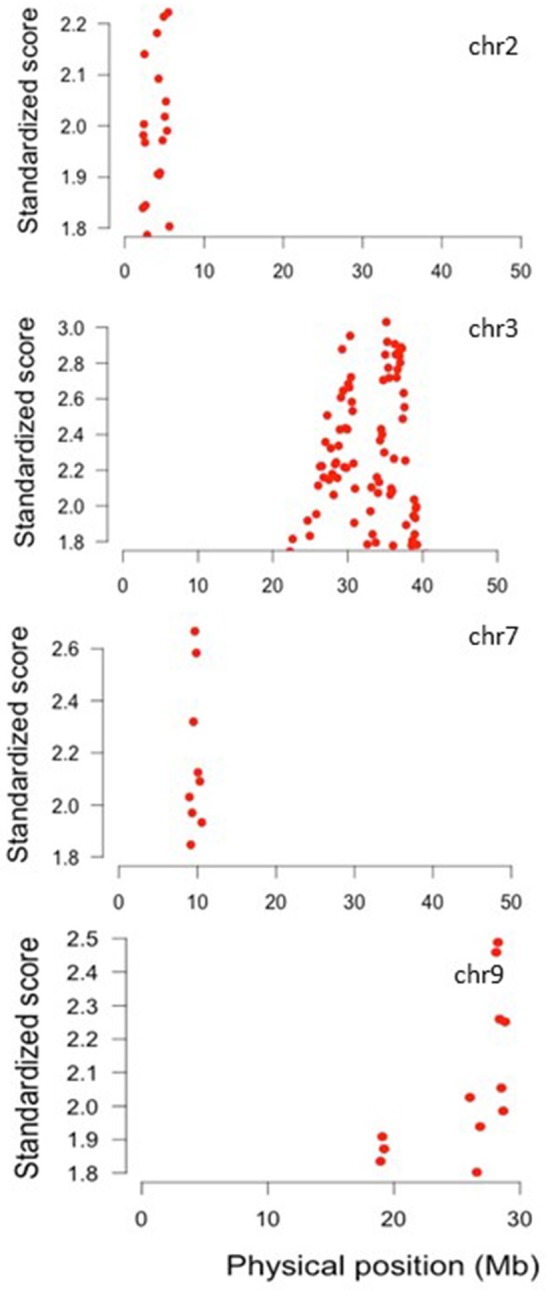
Standardized varLD scores in the top 5% of the genome-wide comparisons among wild and domesticated Lima beans on the basis of 4,779 SNPs detected by GBS. Plots show the chromosomes where regions with significant differences in LD between wild and domesticated beans were detected.

The results obtained with the varLD method show that chromosome regions with significant differences in LD between wild and domesticated beans are located in only four chromosomes as shown in Figure 10. It can be noted that the SNP in chromosome three detected as outlier by Bayescan (Table S7) and most of outlier SNPs in chromosome three detected by Arlequin (Table S8) lie within the region of high LD detected by varLD in this chromosome.

As stated in materials and methods, in order to establish the presence of domestication candidate genes within the regions detected by varLD, we counted the number of genes that displayed domestication signatures in *P. vulgaris*, according to the results reported by Schmutz et al. (2014), and then we evaluated whether the function of these genes was or not related to domestication on the basis of the function reported for *Arabidopsis thaliana*. Schmutz et al. (2014) identified candidate domestication SNPs as those SNPs that showed significant reduction in genetic diversity in the landraces and those showing significantly higher F_ST_ values among wild and domesticated pooled samples.

With this approach we found 150 genes in total and five of them seemed to be involved in functions related to several traits of the domestication syndrome (seed germination, growth promotion, flowering regulation, pod shattering, and photoperiod sensitivity). The results are summarized in Table S9.

## Discussion

### Gene pools and genetic diversity in wild lima bean

The new sequencing technologies allowed us evaluate the genetic structure of wild Lima bean on the basis of thousands of genome-wide SNP markers and confirm, on a more solid basis, the existence of three gene pools: the Mesoamerican I, the Mesoamerican II, and the Andean (AI), with mostly non-overlapping geographic ranges. The results also suggest the possible existence of another gene pool in central Colombia (AII) (although more samples need to be analyzed).

The different analysis tools used, namely, NJ topologies, PCoA, and Structure assignment tests, agreed in the description of the genetic structure of wild Lima bean into three gene pools. Inside the Mesoamerican gene pools (MI and MII) we did not find evidence for further subgroups in a geographical sense, as earlier suggested by Andueza-Noh et al. (2015) who found on the basis of SSR markers a sub-structuring within the gene pool MI in central-western Mexico.

The finding in this study of a possible existence of a separate cluster (AII) for wild Lima bean accessions from the departments of Cundinamarca and Boyacá in central Colombia is not new. Caicedo et al. (1999) had already observed the existence of the Mesoamerican and Andean gene pools in Lima bean and the existence of a separate cluster in the Colombian departments of Cundinamarca and Boyacá on the basis of AFLP markers. In earlier years, wild accessions of Lima bean from Cundinamarca and Boyacá were collected by CIAT in 1992 (Toro et al., 1993) and 1993, and these wild Lima beans were reported to be morphologically similar to the Andean forms in western Ecuador and northwestern Peru in terms of flowers and seed size, but similar to the Mesoamerican Lima beans in terms of electrophoretic patterns of phaseolin (the main reserve seed protein). Therefore, one could wonder about the evolutionary significance of these wild populations in Colombia. Previous studies have established that the Andean gene pool AI is ancestral to MII and MI (Serrano-Serrano et al., 2010), and in this context gene pool AII takes relevance to understand the evolutionary history of wild Lima bean and its spread to Mesoamerica from the Andes, therefore additional accessions from central Colombia should be studied in more detail.

When we compared our current GBS results with previous studies based on ITS and cpDNA data (Motta-Aldana et al., 2010; Serrano-Serrano et al., 2012), we observed that there are some few conflicts or discrepancies in the classifications of some wild accessions (in total 12, see Table S3) into Andean vs. Mesoamerican gene pool. For example, within the wild Mesoamerican gene pool MII, the accessions from Ecuador (Azuay, G26721), and Peru (Cajamarca, G25913) displayed, in previous studies, ITS and cpDNA haplotypes typical of Andean beans. This conflict could be explained by introgression among Andean wild and domesticated Mesoamerican beans introduced to the Andes or by a phenomenon known as incomplete lineage sorting that can produce conflicts among phylogenetic trees built on the basis of different genomic regions. The first explanation is at least possible for the accession from Ecuador (Azuay) because Mesoamerican landraces could be found at the elevation where this wild accession was collected (470 meters above sea level). For the accession in Peru (Cajamarca) this explanation is less plausible because of the high altitude in which this accession was collected (1,810 m.a.s.l.). In the wild Mesoamerican gene pool MI we also found conflicts. In this gene pool, two wild accessions from Ecuador (G26469 and G26751A) showed large seed sizes (18.5 and 25.4 g, respectively) that are within the range of Andean wild Lima beans and in a previous study (Motta-Aldana et al., 2010), based on ITS and cpDNA polymorphisms, these two accessions were classified as Andean (see Table S3). Another accession (G26606) showed a seed size (14.9 g) within the range of Mesoamerican wild beans and had been classified in previous studies as Mesoamerican based on ITS data and as Andean based on cpDNA data (Motta-Aldana et al., 2010). Therefore, these three accessions may represent cases of ancient introgression between Mesoamerican and Andean beans in Ecuador.

For the Andean wild gene pool we found an unexpected result. In previous studies (Motta-Aldana et al., 2010; Serrano-Serrano et al., 2012), all wild accessions that were classified within the Andean gene pool were distributed in Ecuador-northern Peru, a well-defined geographic area. In the present study, we found within this gene pool accessions from Ecuador and northern Peru with a seed size range that goes from 13 to 22.2 g/100 seeds typical of the Andean wild beans, and also found wild accessions from the Mesoamerican area (from Mexico, Guatemala, and Honduras). These accessions from Mesoamerica show seed sizes from 8 to 11.6 g/100 seeds, a range within the one observed in Mesoamerican wild beans. In fact, in a previous study (Serrano-Serrano et al., 2012) based on ITS polymorphisms, these accessions from Mesoamerica were classified within the gene pool MII, not within the Andean gene pool (see Table S3). Seed size and ITS data suggest a Mesoamerican origin for these accessions, but GBS data placed them closer to the Andean wild populations of Ecuador-northern Peru, a result that is difficult to explain.

Apart from these few cases of conflict, where some of them may be explained by introgression, we found large agreement between GBS and ITS data. This result is remarkable given the fact that although the ITS represents a single locus in the nuclear genome, this locus resulted to be very informative about the genetic structure of Lima bean.

In terms of genetic diversity, GBS data show that gene pool MI (H_E_ = 0.115) and gene pool MII (H_E_ = 0.138) are very similar; H_E_ for MII is only slightly higher than for MI. This pattern contrasts with previous studies based on ITS and cpDNA haplotypes where the gene pool MI was found to be more diverse than gene pool MII, and indeed this pattern was the basis for a test applied by Serrano-Serrano et al. (2010) that inferred processes of population expansions within MII that would have reduced its genetic diversity. Our results are in agreement with a previous study based on nuclear DNA SSR polymorphisms (Andueza-Noh et al., 2015) where the authors found more diversity in the wild MII gene pool (H_E_ = 0.65) than in the wild gene pool MI (H_E_ = 0.53). This observation is also in agreement with the statistics calculated in the ABC approach that showed that wild gene pool MI (H_E_ = 0.1396) is less diverse than wild gene pool MII (H_E_ = 0.2059). The higher diversity of wild gene pool MII may be related to its larger effective size (see Table 4, see parameter *N4*) and larger geographic range, from southern Mexico to northern Argentina, compared to the geographic range of gene pool MI mainly in central-western Mexico and to the north-end of the distribution range of the species.

### Demography of lima bean domestication and founder effects

In this study we first applied an indirect approach (NJ, PCoA and Structure assignment test) to produce domestication scenarios that were later tested by means of an ABC approach. The indirect approach (see Figures 6–8) showed that domesticated accessions grouped together with wild accessions into three different clusters: MI, MII, and AI. At first sight, this clustering pattern could indicate three separate domestication events: one for MI, one for MII, and another one for AI.

By comparing Figures 6–8, it can be seen that the results of NJ, PcoA, and Structure are in general congruent in the clustering pattern of accessions (see Table S1, last two columns). The only major exception is that while in the NJ we observed the six wild Colombian accessions grouped in a well-supported cluster (cluster AII), in the Structure analysis these accessions were assigned to gene pool MII. This result makes evident the need, in future studies, for a more exhaustive analysis of the wild accessions from central Colombia to better establish their genetic relationships to the other gene pools.

An interesting result is that most of the Mesoamerican landraces analyzed (68 in total) are grouped within cluster MI along with wild accessions from this gene pool (Figure 6). In this cluster we found three large-seeded landraces from the Andes, which in previous studies (Serrano-Serrano et al., 2012) based on ITS polymorphisms were classified as Andean, therefore we believe these are actually Andean accessions but may have introgressed with introduced Mesoamerican landraces in the Andes. Another interesting result is the fact that within the MI cluster, the landraces form a separate subcluster, which may indicate a single origin for these accessions (one domestication event). In contrast, we see that only a handful of Mesoamerican landraces (14 in total) are grouped within the MII cluster along with wild accessions of this gene pool. In this MII cluster we also found three large-seeded landraces from the Andes, which in previous studies (Serrano-Serrano et al., 2012) based on ITS polymorphisms were also classified as Andean, therefore we also believe these are actually Andean accessions but may have introgressed with Mesoamerican landraces introduced in the Andes (see Table S3). In this MII cluster, the landraces do not form a single subcluster but are interspersed among the wild accessions (see Figure 6). This clustering pattern within MII may be compatible with a scenario of introgression among MII wild accessions and introduced domesticated accessions, however a second domestication event accompanied with profuse gene flow cannot be ruled out. The indirect approach therefore suggested two domestication scenarios (see Figure 1) that were evaluated with an ABC approach. The first scenario involves a domestication event within gene pool MI and another domestication event within gene pool MII. The domestication event within MI would have occurred in the distribution area of wild MI accessions, which are mainly distributed in central-western Mexico, making this the putative domestication area for the MI Mesoamerican landraces. The domestication area for MII landraces would have occurred in the area Guatemala-Costa Rica where the wild MII accessions are more abundantly distributed in Mesoamerica. The second domestication scenario involves only a single domestication within MI followed by admixture between MI domesticates and MII wild populations.

According to the posterior probabilities calculated with the ABC approach, both scenarios seem to be supported by the genetic data, depending on the approach that one applies: the direct approach gives more support to scenario 1 while the logistic approach only supports scenario 2. This conflict among the direct and logistic approach may reflect conflictual molecular signals and not implementation problems because we have shown in the results a good prior-model combination and also, type I and type II errors were low (see Table 3), suggesting enough statistical power to differentiate among the competing scenarios.

A single domestication event from gene pool MI, which is mainly distributed in central-western Mexico, has been supported by all previous studies based on cpDNA, ITS and SSR polymorphisms (Motta-Aldana et al., 2010; Serrano-Serrano et al., 2012; Andueza-Noh et al., 2013, 2015), in contrast, all the data collected in previous studies have not confirmed a second domestication event in Lima bean from gene pool MII. On the basis of the high support obtained by scenario 2 with the logistic approach (posterior probability of 100%), we can say that our genetic data give evidence for a contribution of wild gene pool MII in shaping the current genome diversity of landraces through admixture events. Some other lines of evidence observed in this study also support an admixed origin of MII domesticates. First, a lack of reduction in overall genetic diversity in MII landraces suggests a contribution of wild relatives to increase diversity levels. Second, the fact than more than half of the loci tested in MII landraces contain higher genetic diversity than wild populations is compatible with the hypothesis of an admixed origin of MII landraces. Third, the fact that MII domesticates do not form a monophyletic clade and that only represent a small percentage of the domesticated accessions suggests an origin of these landraces through admixture events. Finally, in some places where MII domesticates were found, for example in the Peninsula of Yucatan, introgression among wild and domesticates has been well-documented (Martínez-Castillo et al., 2007; Dzul-Tejero et al., 2014). With this in mind, our discussion on parameters of the domestication process of Lima bean estimated by the ABC approach will continue mainly on the basis of scenario 2.

Two parameters of interest for the domestication process is the population size at the beginning of the process, or in other words the population size of the founders, and the duration of the population bottleneck. These parameters are of interest because they affect the current genetic diversity of the crop and will be discussed below. For the domestication event in gene pool MI, the size of the founder population (*N2b* in the models) varies from 90 to 1,980 individuals (with mean 498), namely a size below 2,000 individuals (see Table 4). This contrasts with the estimation of the current size of the wild ancestor MI (*N1* in the models) that varies from 97,000 to 300,000 individuals (with mean 204,000). If we compare these estimates (*N1* and *N2b*), these results would suggest a drastic reduction in population size (a reduction of about 99%). These results are in contrast with those observed by Mamidi et al. (2011) in common bean, where sequences of 13 loci were analyzed by coalescent simulations and the authors observed that the bottleneck effective size within the Mesoamerican gene pool was around 48% the size of the wild ancestor. Both parameters (*N1* and *N2b*) have bias and error measures relatively small and genetic data are informative to estimate these parameters (in Table S6 it can be seen that estimated means for parameters *N1* and *N2b* are close to the true values, and the values without taking into account the genetic data are more biased). The duration of the bottleneck for the MI domestication (parameter *db1* in the models) varied from 2,100 to 3,000 years (average of 2,870 years). However, for this parameter genetic data seem not to be very informative given the fact that the estimation of the parameter, bias and errors with and without genetic data are very similar.

Taking together the parameters *N2b* and *db1* we can calculate the bottleneck intensity, *k* = *N2b*/*db1*, in around 0.17, which indicates a strong founder effect for the MI gene pool, because of a small founder population and a long duration of the bottleneck. This would explain the 30% reduction in genetic diversity observed within this gene pool (Table 2). Our results are in contrast with those obtained for other crops such as soybean and maize, where the *k* ratio was 2 or 4–5, respectively, namely the bottleneck population size was higher than the bottleneck duration, therefore these crop species showed a bottleneck not so severe as the one observed here in Lima bean (Tenaillon et al., 2004; Guo et al., 2010). The results of the present study are in agreement with reports in rice where a domestication intensity of *k* = 0.2–0.5 was observed, with a founding population between 400–500 individuals and a long bottleneck duration (at most 3,000 years, similar to the estimation in Lima bean) (Zhu et al., 2007). It is difficult to establish why Lima bean shows a severe domestication bottleneck in gene pool MI. A selfing mating system could be an explanation because of the reduced chances of crossing with wild relatives, however not all selfing legume species show severe bottlenecks, soybean for example shows a moderate bottleneck (Guo et al., 2010). Therefore, mating system may not be key in determining strength of domestication bottleneck. Another explanation for a severe domestication bottleneck in Lima bean could be that domestication occurred only once in a very reduced area, mainly due to the presence of high cyanogenic glucoside in wild populations that would limit the number of times these beans were taken into domestication, as suggested elsewhere (Debouck, 1996), however we do not have data to test this hypothesis. An additional explanation is that in their migration from the original domestication site to other regions within Mesoamerica, the early domesticates suffered additional bottlenecks.

Another parameter of interest is domestication time. According to scenario 2, domestication within gene pool MI, presumably in central-western Mexico, would have started 7,700 years ago (parameter *t2*) and after an unknown time, the expansion of the first domesticates to other regions would have started. Presumably, domesticated Lima beans would have migrated toward the south and east of Mexico, in the distribution area of wild gene pool MII with which it experienced admixture events at a time estimated by parameter *t1* to be 6,500 years ago. The oldest archeological remains that show the presence of domesticated Lima beans in the distribution area of gene pool MII are those from the site known as Dzibichaltún in the Peninsula of Yucatan in Mexico, with an age of about 1,300 years before present (Kaplan, 1965). This archeological site is within the distribution range of wild populations in the Peninsula of Yucatan, therefore the introgression events among wild and domesticated beans in this site of Mexico could be ancient.

Global founder effects estimated in this study were about 18%, this means that landraces retain in average around 82% of the variation found in wild populations. This reduction is in agreement with a previous study of SSR markers on Lima bean landraces where the authors found a global reduction of genetic diversity in landraces of about 17% (Andueza-Noh et al., 2015). This result also agrees with the study of Schmutz et al. (2014) who carried out the sequencing of the genome of *P. vulgaris* and resequencing of Mesoamerican and Andean wild and landrace accessions and observed a reduction of about 17% in genetic diversity (measured as nucleotide diversity) in Mesoamerican landraces.

When reduction in genetic diversity in Lima bean was calculated within gene pools, contrasting patterns were found. Within gene pool MI, reduction in genetic diversity was about 30% and no reduction was observed within gene pool MII, the latter result being compatible with the hypothesis of an admixed origin of MII landraces. These values are also in large agreement with previous reports in Lima bean in the sense that there was evidence of a large founder effect within gene pool MI (about 44%), while within gene pool MII it was almost negligible (only 1%) (Andueza-Noh et al., 2015).

In this study we could measure founder effects at a locus-by-locus basis and found different patterns as shown in the results section (see Table 2). In gene pool MI we can see a global reduction of genetic diversity in landraces of about 30%, but when this reduction is estimated locus-by-locus a larger loss in genetic diversity (in average 78%) was seen in 85% of the loci analyzed. Interestingly, 15% of loci within gene pool MI showed increased diversity in landraces in comparison to their wild counterparts. These results suggest that most loci are losing genetic diversity maybe due to the demographic effects of domestication and also by selection forces (see below). The loci where an increase in diversity was observed in landraces may represent regions in the genome that contain genes that were useful for landrace diversification during the adaptation process or genes that underwent introgression with other landraces or wild populations (Burger et al., 2008).

When global reduction in genetic diversity was measured within gene pool MII, no loss in genetic diversity was observed. However, when this measure was done locus-by-locus it was observed that about 45% of loci showed a reduction in diversity (in average a reduction of 53%) and that 55% of loci showed an increase in diversity among landraces (an increase of around 33%). The same was observed within gene pool AI where no reduction in genetic diversity among landraces was observed at a global scale but when measured locus-by-locus, an average reduction of 47% was observed in 79% of loci, as expected for a domestication event. Clearly, the MII landraces show a locus-by-locus pattern that is different from the one observed in MI and AI landraces because in MII landraces most loci showed an increase in genetic diversity. This result might be compatible with and admixed origin for these landraces.

It is well-known that founder effects result in increasing levels of linkage disequilibrium (*r*^2^) in the genome. In this study, wild accessions showed pairwise *r*^2^ values, averaged per chromosome, that went from 0.12 to 0.14, while landraces showed pairwise *r*^2^ values, averaged per chromosome, between 0.15 and 0.19, a global increase in *r*^2^ of about 20–30%. Similar results in pairwise LD levels were observed in common bean in a previous study (Rossi et al., 2009) where average *r*^2^ was 0.08 for wild accessions and 0.18 for domesticated accessions, an increase of 55%. The increase in LD levels may be a result of the reduction in genetic diversity during the domestication process as a consequence of the domestication bottleneck that in the gene pool MI was strong, and also as a consequence of selective forces acting during adaptation. Below we are discussing possible genomic regions affected by selection.

### Outlier loci and genomic regions related to domestication

We applied two complementary approaches to detect loci related to domestication as F_ST_ outliers. With these approaches, a total of 26 F_ST_ outlier SNPs were detected (see Tables S7, S8). Interestingly, all F_ST_ outlier SNPs detected in chromosome three co-localized within or nearby the LD region detected by varLD in this chromosome. Of these 26 SNPs, five involved missense changes in five genes. One of these changes is in the gene Phvul.001G146000, whose *Arabidopsis* ortholog is the *Growing Slowly* (GRS1) gene, a gene that has functions in RNA editing and plant development (mutants show slow growth and sterility) (Xie et al., 2016). A second missense change was found in the gene Phvul.003G176700, whose *Arabidopsis* ortholog is the *Histone deacetylase 15* (HDA15) gene, a negative regulator of expression involved, with other genes, in repression of chlorophyll biosynthesis, photosynthesis, photomorphogenesis, and seed germination in the dark (Liu et al., 2013; Gu et al., 2017). A third missense change was found in the gene Phvul.002G165600, a gene ortholog to the *Albina 1* (ALB1 o CHLD) gene in *Arabidopsis* also involved in chlorophyll biosynthesis (Papenbrock et al., 1997). Another interesting missense SNP was located in the gene Phvul.004G099700, involved in defense response to fungus according to its GO.

Given that selection is expected to increase LD levels beyond the genome-wide LD increase caused by the domestication bottleneck, we expected to be able to detect those regions by means of inter-population (wild vs. domesticated) LD comparisons by the varLD approach. By applying this approach, we identified significant regions in chromosomes two, three, seven, and nine (see Figure 10). The small number of regions detected when using the LD approach might be due to the fact that, because of the confounding effects of demographic events over selection signals, background levels of LD had to be taken into account before detecting regions undergoing selection, thus rendering the varLD tests very conservative. Complementary strategies not taken in this study for the detection of regions containing domestication genes are genome-wide association (GWAS) approaches and QTL linkage mapping. In this regards, it is interesting to see that in a GWAS study carried out in common bean to detect genes related to change in seed size during domestication (Schmutz et al., 2014), many of the candidate genes detected in that study co-localize with the LD regions detected in the present study in chromosomes three (25.0–40.0 MB) and seven (9–10.5 MB). The authors found that in common bean the region in chromosome seven that contained seed size candidate genes also showed extensive LD (Schmutz et al., 2014), as we also observed in the present study. Within the chromosome regions in high LD observed in this study, we could locate 150 of the domestication candidate loci identified in common bean (Schmutz et al., 2014) (see Table S9). Although these 150 candidate loci constitute a good starting point, they need to be cross-validated with complementary approaches such as QTL mapping by linkage analyses, GWAS, candidate association studies, selection tests of DNA sequences and differential gene expression, among others.

Among the 150 genes, we found five candidate loci involved in seed germination, organ size, pod dehiscence, and flowering time, all functions related to the domestication syndrome. Within the LD region in chromosome 2 we found two candidate genes: Phvul.002G033500 (start: 3,391,469, end: 3,393,850) and Phvul.002G041800 (start: 3,983,255, end: 3,986,921). The best *A. thaliana* hit for the gene Phvul.002G033500 is AT5G66460 (Endo-B-Mannanase gene 7, MAN7), a gene encoding an Endo-B-Mannanase, a hydrolitic enzyme that degrades the mannan polymer, the main constituent of the cell wall in the endosperm of seeds and therefore plays a crucial role in seed germination (Iglesias-Fernández et al., 2013). Increased seed germination was a key trait selected during crop domestication that allowed the adaptation of plants to the cultivated fields, therefore this is a good candidate gene. The best *A. thaliana* hit for gene Phvul.002G041800 is AT3G13960 (growth regulating factor 5, GRF5), a gene that encodes a transcription factor that promotes cell proliferation during leaf development, promotes leaf longevity, stimulates chloroplast division with a correlated increase in chlorophyll content and photosynthetic rate and increase tolerance to grow in nitrogen-depleted soil (Horiguchi et al., 2005; Vercruyssen et al., 2015). It has been shown that over-expression of *A. thaliana* GRF5 along with AN3 (the product of the gene Angustifolia 3, another transcription factor) increases leaf size (Horiguchi et al., 2005). It is well-known that one of the changes in plant domestication has been the increase in size of some organs such as flowers, fruits, and leaves. Therefore, the relevance of genes that promote growth of different organs during domestication seems plausible.

Within the LD region in chromosome 7 we found the gene Phvul.007G096500 (start: 10,156,828; end: 10,169,477), whose best *A. thaliana* hit was the gene AT5G04240 (the early flowering gene 6, *elf* 6), which is involved in the regulation of flowering. Flowering time is an important trait that allows adaptation and spread of early domesticates into regions with diverse photoperiod regimes. Lima bean is an excellent example for this kind of adaptation given the wide latitudinal range that this species explores in the wild and under cultivation. In *Arabidopsis* there are two pathways that control flowering according to environmental stimuli: the photoperiod pathway and the vernalization pathway, that respond to day length and temperature, respectively (Mouradov et al., 2002). The *elf* 6 gene encodes a nuclear protein with jumonji and zinc finger domains that plays a role as an upstream repressor in the photoperiod pathway (Noh et al., 2004). *Arabidopsis elf* 6 mutants display early flowering under long and short days, therefore we consider this gene as a good candidate gene for further study.

In chromosome 9 we observed that the gene Phvul.009G203400 (start: 30,080,598, end: 30,089,684) falls very close to the region in high LD in this chromosome. The best *A. thaliana* hit for this gene is AT5G60910 (FRUITFULL or FUL), a MADS-Box gen that expresses in the cell layers of the valve tissues of the silique in *Arabidopsis* (Gu et al., 1998) and control the transcription of other MADS-Box genes such as SHP1/2 (SHATTERING PROOF 1 and 2) required for fruit development and dehiscence or pod shattering (Liljegren et al., 2000), a key trait for crop domestication.

Also in chromosome 9, the gene Phvul009G117500 (start: 17,541,857, end: 17,548,419) falls very close to the high LD region in this chromosome. The best *A. thaliana* hit for this gene is AT5G17690 (TERMINAL FLOWER 2, TFL2) a gene that controls flowering time and photoperiod sensitivity and regulates the expression of other flowering time genes and other floral organ identity genes (Larsson et al., 1998; Kotake et al., 2003). As indicated above, control of flowering time is key for the adaptation of early domesticates to other regions with different photoperiod regimes and therefore this gene is also a good candidate.

## Conclusions

In summary we can say that the GBS approach resulted very useful to discover SNP markers for evolutionary studies in wild and domesticated Lima beans. The SNP markers and clustering and Bayesian approaches applied let us confirm the existence of three gene pools in wild Lima beans, the Mesoamerican one (MI), the Mesoamerican two (MII), and the Andean one (AI), with mainly non-overlapping geographic ranges, and also suggest the existence of another Andean gene pool (AII) in central Colombia, although additional information is needed. The ABC approach was very useful to test competing domestication scenarios for Lima bean Mesoamerican landraces. The scenario that was better supported with the logistic regression approach was a single domestication event within gene pool MI for all Mesoamerican landraces, maybe in central-western Mexico, and subsequent admixture among landraces and wild populations within the distribution range of gene pool MII that gave rise to MII landraces. Locus-by-locus analyses of genetic diversity showed that domestication founder effects were strong within gene pools MI and AI, as expected for domestication events, but less drastic for gene pool MII, which is compatible with an admixed origin of MII landraces. After accounting for background increase in LD levels due to the domestication bottleneck, we were able to detect genomic regions with significant differences in LD among wild and domesticated accessions that may represent regions affected by selection processes and that may harbor domestication genes. A search for domestication candidate genes within these LD regions, on the basis of candidate genes reported for common bean, resulted in a list of 150 genes, among them genes related to seed germination, organ size, pod dehiscence, and flowering time. Follow-up studies should include analysis of additional samples from central Colombia in order to gather more evidence about the possible existence of a separate wild gene pool and complementary approaches to map genes related to domestication.

## Author contributions

MC conceived the idea for the research project, carried out laboratory techniques for extraction of DNA to acquire GBS data, analyzed GBS data and produced the first draft of the manuscript. JM collected and provided plant germplasm of Lima bean from Mexico, carried out laboratory techniques for extraction of DNA to acquire GBS data and revised critically the manuscript.

### Conflict of interest statement

The authors declare that the research was conducted in the absence of any commercial or financial relationships that could be construed as a potential conflict of interest.
