# Supplementary material for: Testing Domestication Scenarios of Lima Bean (Phaseolus lunatus L.) in Mesoamerica: Insights from Genome-Wide Genetic Markers
Source: Front Plant Sci. 2017 Sep 12;8:1551. doi: 10.3389/fpls.2017.01551 (PMC5601060; doi:10.3389/fpls.2017.01551)
Supplement: Supplementary file 1 [file Table1.PDF]

Supplementary Table S1. Passport data of the 160 wild and 110 domesticated accessions of Lima bean included in this study and their classification according to PcoA and Structure results. Accessions with an asterisk in their ID are those selected for the ABC approach.

ID	Institution	Biological status	Country	Department	Municipality	Elevation (msnm)	Latitude	Longitude	Weight of 100 seeds (g)	PcoA results	Structure results
G25290A	CIAT	Domesticated	Guatemala	Sacatepequez	Antigua Guatemala	1530	14.55	-90.7167	22.1	Dom Admixed	Dom Admixed
G25766	CIAT	Domesticated	Mexico	Campeche	Hecelchakan	20	20.1333	-90.2167	39	Dom Admixed	Dom Admixed
G25910	CIAT	Domesticated	Colombia	Nariño	Guachavez	1900	1.25	-77.6667	60	Dom Admixed	Dom Admixed
G25943	CIAT	Domesticated	Peru	Cajamarca	Santa Cruz	1580	-6.5833	-78.9167	34.7	Dom Admixed	Dom Admixed
G25987	CIAT	Domesticated	Bolivia	Chuquisaca	Tomina	1930	-19.1667	-64.5167	102.1	Dom Admixed	Dom Admixed
G26451	CIAT	Domesticated	Panama	Chiriqui	David		8.3333	-82.3333	42	Dom Admixed	Dom Admixed
G26656	CIAT	Domesticated	Colombia	Valle Del Cauca	Candelaria	1000	3.4167	-76.3167	70.5	Dom Admixed	Dom Admixed
G27448	CIAT	Domesticated	Colombia	Bolivar	El Carmen	160	9.7167	-75.1167	16.2	Dom Admixed	Dom Admixed
G25108	CIAT	Domesticated	Brazil	Minas Gerais	Vicosa	719	-20.75	-42.8833	83	Dom AI	Dom K3
G25277	CIAT	Domesticated	El Salvador	Cuscatlan	Cuscatlan	800	13.7	-88.9667	40	Dom AI	Dom K3
G25420	CIAT	Domesticated	Peru	Piura	Talara		-4.5667	-81.2833	117	Dom AI	Dom K3
G25540	CIAT	Domesticated	Argentina	Salta	Coronel Moldes	1143	-25.2667	-65.5	70	Dom AI	Dom K3
G25771	CIAT	Domesticated	Mexico	Campeche	Hecelchakan	60	20.2	-89.9	47	Dom AI	Dom K3
G25908	CIAT	Domesticated	Colombia	Putumayo	Sibundoy	2040	1.2	-76.8333	95	Dom AI	Dom K3
G26290	CIAT	Domesticated	Argentina	Formosa	Formosa	200	-26.1833	-58.1833	40	Dom AI	Dom K3
G26438	CIAT	Domesticated	Costa Rica	Cartago	Turrialba	750	9.9	-83.6833	53	Dom AI	Dom K3
G26505	CIAT	Domesticated	Colombia	Cundinamarca	La Mesa	1650	4.6833	-74.45	58.4	Dom AI	Dom K3
G26578	CIAT	Domesticated	Ecuador	Pichincha	Puellaro	1990	0.1333	-78.4167	48.2	Dom AI	Dom K3
G26617	CIAT	Domesticated	Colombia	Boyaca	Somondoco	1900	4.9667	-73.45	78.2	Dom AI	Dom K3
G25216*	CIAT	Domesticated	Guatemala	Alta Verapaz	San Cristobal Verapaz		15.3833	-90.4	40.8	Dom MI	Dom K2
G25224A*	CIAT	Domesticated	Costa Rica	Guanacaste	Ca_as	100	10.7667	-85.4833	47	Dom MI	Dom K2
G25267*	CIAT	Domesticated	El Salvador	San Salvador	San Salvador		13.7	-89.2167	42	Dom MI	Dom K2
G25278	CIAT	Domesticated	El Salvador	Cuscatlan	Cuscatlan	800	13.7	-88.9667	48	Dom MI	Dom K2
G25293*	CIAT	Domesticated	Mexico	Guerrero	Iguala		17.85	-100.3667	49.9	Dom MI	Dom K2
G25364*	CIAT	Domesticated	El Salvador	La Libertad	Nueva San Salvador	965	13.6667	-89.3	41.8	Dom MI	Dom K2
G25366	CIAT	Domesticated	El Salvador	Santa Ana	Texistepeque	400	14.1167	-89.4833	46	Dom MI	Dom K2
G25397*	CIAT	Domesticated	Costa Rica	Alajuela	Alajuela	940	10	-84.2	79	Dom MI	Dom K2
G25410*	CIAT	Domesticated	Ecuador	Los Rios	Quevedo	106	-0.9833	-79.45	32	Dom MI	Dom K2
G25541*	CIAT	Domesticated	Argentina	Tucuman	San Miguel De Tucuman		-26.8167	-65.2167	37.4	Dom MI	Dom K2

ID	Institution	Biological status	Country	Department	Municipality	Elevation (msnm)	Latitude	Longitude	Weight of 100 seeds (g)	PcoA results	Structure results
G25542	CIAT	Domesticated	Argentina	Corrientes	Goya		-28.9667	-59.0833	47	Dom MI	Dom K2
G25543	CIAT	Domesticated	Argentina	Santiago Del Estero	Santiago Del Estero	199	-27.8	-64.25	40	Dom MI	Dom K2
G25614*	CIAT	Domesticated	Mexico	Oaxaca	San Fco. Telixtlahuaca	1520	17.3	-96.9	60	Dom MI	Dom K2
G25700A	CIAT	Domesticated	Panama	Cocle	Toabre	200	8.6667	-80.2833	68	Dom MI	Dom K2
G25705*	CIAT	Domesticated	Mexico	Campeche	Hopelchen	100	19.4167	-89.7	34	Dom MI	Dom K2
G25735*	CIAT	Domesticated	Mexico	Campeche	Hecelchakan	40	20.2333	-89.9333	40	Dom MI	Dom K2
G25770	CIAT	Domesticated	Mexico	Campeche	Hecelchakan	60	20.2	-89.9	32	Dom MI	Dom K2
G25854*	CIAT	Domesticated	Colombia	Nari_o	Ancuya	1160	1.2333	-77.5167	36	Dom MI	Dom K2
G25909	CIAT	Domesticated	Peru	Cajamarca	San Miguel	2150	-7.0167	-78.85	79	Dom MI	Dom K2
G25971*	CIAT	Domesticated	Mexico	Morelos	Tepoztlan	1850	18.9833	-99.1	40	Dom MI	Dom K2
G25974*	CIAT	Domesticated	Guatemala	Jalapa	San Luis Jilotepeque	750	14.6333	-89.7333	57	Dom MI	Dom K2
G25981*	CIAT	Domesticated	Bolivia	Chuquisaca	Oropeza	1970	-18.95	-65.15	121.5	Dom MI	Dom K2
G26291*	CIAT	Domesticated	Mexico	Guerrero	Tlapehuala		18.1667	-101.4667	50	Dom MI	Dom K2
G26300*	CIAT	Domesticated	Guatemala	Chimaltenango	San Martin Jilotepeque	1700	14.7833	-90.7833	66	Dom MI	Dom K2
G26444*	CIAT	Domesticated	Honduras	Intibuca	La Esperanza		14.2833	-88.25	58	Dom MI	Dom K2
G26480*	CIAT	Domesticated	Ecuador	Imbabura	Ibarra	2050	0.4	-78.1333	64.4	Dom MI	Dom K2
G26490	CIAT	Domesticated	Colombia	Huila	Teruel	850	2.7333	-75.55	32.8	Dom MI	Dom K2
G26512*	CIAT	Domesticated	Mexico	Guerrero	Ajuchitlan Del Progreso		18.1667	-100.4833	53.6	Dom MI	Dom K2
G26513*	CIAT	Domesticated	Mexico	Guerrero	San Miguel Telolopan		18.1167	-100.2667	46	Dom MI	Dom K2
G26529*	CIAT	Domesticated	Colombia	Guajira	Fonseca		10.9	-72.85	49.7	Dom MI	Dom K2
G26534*	CIAT	Domesticated	Mexico	Chiapas	Venustiano Carranza	890	16.3333	-92.4333	40	Dom MI	Dom K2
G26542*	CIAT	Domesticated	Mexico	Puebla	Tehuacan	1310	18.5	-97.2167	52.4	Dom MI	Dom K2
G26647	CIAT	Domesticated	Colombia	Antioquia	Ebejico	1390	6.3667	-75.6667	38	Dom MI	Dom K2
G26706	CIAT	Domesticated	Colombia	Caldas	Aguadas	1485	5.6167	-75.5333	37.3	Dom MI	Dom K2
G26714	CIAT	Domesticated	Ecuador	Manabi	Chone	67	-0.6667	-80.0833	39.3	Dom MI	Dom K2
G27350A	CIAT	Domesticated	Ecuador	Azuay	Paute	2180	-2.8667	-78.8333	23	Dom MI	Dom K2
G27358*	CIAT	Domesticated	Peru	San Martin	San Martin	425	-6.5	-76.3667	33	Dom MI	Dom K2
G27381	CIAT	Domesticated	Honduras	Intibuca	La Esperanza		14.3	-88.1833	107.4	Dom MI	Dom K2
G27388*	CIAT	Domesticated	Mexico	Guerrero	Zitlala		17.6333	-99.0833	55.7	Dom MI	Dom K2
G27399*	CIAT	Domesticated	Mexico	Veracruz	Papantla		20.4833	-97.45	42	Dom MI	Dom K2
G27422	CIAT	Domesticated	Colombia	Magdalena	Plato	100	9.8333	-74.3333	32.6	Dom MI	Dom K2
G27429*	CIAT	Domesticated	Colombia	Cordoba	Sahagun	60	8.95	-75.45	38.4	Dom MI	Dom K2

ID	Institution	Biological status	Country	Department	Municipality	Elevation (msnm)	Latitude	Longitude	Weight of 100 seeds (g)	PcoA results	Structure results
G27445*	CIAT	Domesticated	Colombia	Atlantico	Juan De Acosta	50	10.8333	-75.0333	21.1	Dom MI	Dom K2
G27455*	CIAT	Domesticated	Colombia	Sucre	Chalan	100	9.55	-75.3167	30.4	Dom MI	Dom K2
JMC 1024	CICY	Domesticated	Mexico	Campeche		130	20.003569	-89.747772		Dom MI	Dom K2
JMC 1035	CICY	Domesticated	Mexico	Campeche						Dom MI	Dom K2
JMC 1048	CICY	Domesticated	Mexico	Campeche		70	19.8	-89.329167		Dom MI	Dom K2
JMC 1061	CICY	Domesticated	Mexico	Quintana Roo		30	19.858611	-88.175		Dom MI	Dom K2
JMC 1104	CICY	Domesticated	Mexico	Petén		198	16.994	-89.91775		Dom MI	Dom K2
JMC 1105	CICY	Domesticated	Mexico	Petén		198	16.994	-89.91775		Dom MI	Dom K2
JMC 1106	CICY	Domesticated	Mexico	Petén		198	16.994	-89.91775		Dom MI	Dom K2
JMC 111	CICY	Domesticated	Mexico	Quintana Roo		10	20.358889	-88.036389		Dom MI	Dom K2
JMC 112	CICY	Domesticated	Mexico	Yucatán		27	20.599031	-88.163444		Dom MI	Dom K2
JMC 1179	CICY	Domesticated	Mexico	Quintana Roo		10	21.893611	-105.161389		Dom MI	Dom K2
JMC 1186	CICY	Domesticated	Mexico	Quintana Roo		78	23.484694	-106.476333		Dom MI	Dom K2
JMC 1197	CICY	Domesticated	Mexico	Quintana Roo		78	19.883333	-88.133333		Dom MI	Dom K2
JMC 1199	CICY	Domesticated	Mexico	Yucatán		34	20.369444	-88.086389		Dom MI	Dom K2
JMC 1200	CICY	Domesticated	Mexico	Quintana Roo						Dom MI	Dom K2
JMC 1262	CICY	Domesticated	Mexico	Quintana Roo		15	18.503611	-88.305278		Dom MI	Dom K2
JMC 1265	CICY	Domesticated	Mexico	Quintana Roo		25	19.843056	-88.134444		Dom MI	Dom K2
JMC 1277	CICY	Domesticated	Mexico	Yucatán						Dom MI	Dom K2
JMC 1284	CICY	Domesticated	Mexico	Campeche		80	19.583333	-89.583333		Dom MI	Dom K2
JMC 1288	CICY	Domesticated	Mexico	Yucatán		150	19.978611	-89.488333		Dom MI	Dom K2
JMC 13	CICY	Domesticated	Mexico	Quintana Roo		25	19.494444	-87.994444		Dom MI	Dom K2
JMC 1312	CICY	Domesticated	Mexico	Yucatán		34	20.4375	-88.388889		Dom MI	Dom K2
JMC 1325	CICY	Domesticated	Mexico	Yucatán		100	20.203056	-89.287778		Dom MI	Dom K2
JMC 1335	CICY	Domesticated	Mexico	Quintana Roo		42	19.042444	-88.419264		Dom MI	Dom K2
JMC 1377	CICY	Domesticated	Mexico	Chiapas		2161	16.759478	-92.721631		Dom MI	Dom K2
JMC 1378-A	CICY	Domesticated	Mexico	Chiapas		461	16.623717	-93.100489		Dom MI	Dom K2
JMC 1381-A	CICY	Domesticated	Mexico	Chiapas		608	15.957031	-92.467078		Dom MI	Dom K2
JMC 1382-B	CICY	Domesticated	Mexico	Chiapas		603	15.766	-92.267683		Dom MI	Dom K2
JMC 1386	CICY	Domesticated	Mexico	Chiapas		820	16.758117	-93.373919		Dom MI	Dom K2
JMC 9	CICY	Domesticated	Mexico	Quintana Roo		25	19.494444	-89.994444		Dom MI	Dom K2
JMC 995	CICY	Domesticated	Mexico	Yucatán		40	20.891111	-88.136389		Dom MI	Dom K2

ID	Institution	Biological status	Country	Department	Municipality	Elevation (msnm)	Latitude	Longitude	Weight of 100 seeds (g)	PcoA results	Structure results
G25195*	CIAT	Domesticated	Guatemala	Jalapa	San Pedro Pinula	900	14.7	-89.9	51	Dom MII	Dom K1
G25303*	CIAT	Domesticated	Mexico	Chiapas	Comitan De Dominguez		16.25	-92.1333	32	Dom MII	Dom K1
G25385B*	CIAT	Domesticated	Costa Rica	San Jose	San Jose	1171	9.9833	-84.0667	38	Dom MII	Dom K1
G25388*	CIAT	Domesticated	Costa Rica	San Jose	San Jose	1171	9.9833	-84.0667	56	Dom MII	Dom K1
G25391B*	CIAT	Domesticated	Costa Rica	San Jose	San Jose	1171	9.9833	-84.0667	35	Dom MII	Dom K1
G25559*	CIAT	Domesticated	Mexico	Chiapas	Bochil	1200	17	-92.8833	35	Dom MII	Dom K1
G25581*	CIAT	Domesticated	Bolivia	Santa Cruz	Cordillera	750	-22.2833	-63.25	50	Dom MII	Dom K1
G25596A*	CIAT	Domesticated	Costa Rica	San Jose	Coronado	1200	10.0667	-84.15	52	Dom MII	Dom K1
G25597*	CIAT	Domesticated	Guatemala	Santa Rosa	Cuilapa		14.2667	-90.3	42.3	Dom MII	Dom K1
G25750*	CIAT	Domesticated	Mexico	Campeche	Calkini	20	20.4	-90.2333	34.2	Dom MII	Dom K1
G25787*	CIAT	Domesticated	Mexico	Campeche	Hopelchen	120	19.5833	-89.6	40	Dom MII	Dom K1
G26002*	CIAT	Domesticated	Brazil	Ceara	Campos Sales	557	-7.2	-40.25	59	Dom MII	Dom K1
G26306*	CIAT	Domesticated	Guatemala	Chiquimula	Quezaltepeque	700	14.55	-89.4833	48	Dom MII	Dom K1
G26659*	CIAT	Domesticated	Ecuador	Imbabura	Ibarra	2120	0.3667	-78.1167	111.5	Dom MII	Dom K1
G26672*	CIAT	Domesticated	Ecuador	Carchi	Mira	2170	0.5333	-78.05	75.4	Dom MII	Dom K1
G27289*	CIAT	Domesticated	Cuba	Ciego De Avila	Moron	10	22.1	-78.6333	44.6	Dom MII	Dom K1
G27337*	CIAT	Domesticated	Bolivia	Chuquisaca	Tomina	2980	-19.25	-64.6333	90.3	Dom MII	Dom K1
G25916	CIAT	Wild	Peru	Cajamarca	Chota	1720	-6.4667	-78.8833	19.3	Wild admixed	Wild admixed
G26618	CIAT	Wild	Colombia	Boyaca	Tenza	1440	5.0667	-73.4167	19.4	Wild admixed	Wild admixed
G26700	CIAT	Wild	Colombia	Boyaca	Sutatenza	1220	5.0333	-73.4167	12	Wild admixed	Wild admixed
G27338	CIAT	Wild	Guatemala	Sacatepequez	Sn Juan El Obispo	1560	14.55	-90.7333	11	Wild admixed	Wild admixed
JMC 1108	CICY	Wild	Mexico	Jalisco		700	20.860833	-103.770556		Wild admixed	Wild admixed
G25844	CIAT	Wild	Guatemala	Sacatepequez	Santa Maria De Jesus	1740	14.4833	-90.7167	8.8	Wild AI	Wild K3
G26459	CIAT	Wild	Ecuador	Loja	Catamayo	1580	-3.9333	-79.4	19.4	Wild AI	Wild K3
G26460	CIAT	Wild	Ecuador	Loja	Loja	1800	-3.9	-79.2833	15	Wild AI	Wild K3
G26468	CIAT	Wild	Ecuador	Bolivar	Sn Miguel	870	-1.7833	-79.1333	13	Wild AI	Wild K3
G26545	CIAT	Wild	Ecuador	Loja	Calvas	1800	-4.3833	-79.5833	17.7	Wild AI	Wild K3
G26608	CIAT	Wild	Ecuador	El Oro	Zaruma	800	-3.3833	-79.5333	16.2	Wild AI	Wild K3
G26609	CIAT	Wild	Ecuador	Azuay	Sta Isabel	1570	-3.2167	-79.2	22.2	Wild AI	Wild K3
G26630	CIAT	Wild	Honduras	Olancho	San Francisco De La Paz	830	14.9333	-86.2167	8.7	Wild AI	Wild K3
G26653	CIAT	Wild	Guatemala	Huehuetenango	Sta. Ana Huista	590	15.6833	-91.9	11.6	Wild AI	Wild K3
G26655	CIAT	Wild	Guatemala	Sacatepequez	Alotenango	1000	14.4167	-90.8167	8.3	Wild AI	Wild K3
G26684	CIAT	Wild	Guatemala	Solola	Panajachel	1680	14.7667	-91.1667	11.3	Wild AI	Wild K3
G26704	CIAT	Wild	Colombia	Caldas	Aguadas	1485	5.6167	-75.5333	17	Wild AI	Wild K3

ID	Institution	Biological status	Country	Department	Municipality	Elevation (msnm)	Latitude	Longitude	Weight of 100 seeds (g)	PcoA results	Structure results
G26732	CIAT	Wild	Guatemala	Jalapa	Sn Luis Jilotepeque	1280	14.6667	-89.7833	10.6	Wild AI	Wild K3
G26753	CIAT	Wild	Mexico	Chiapas	Venustiano Carranza	775	16.3333	-92.45	8	Wild AI	Wild K3
G26615	CIAT	Wild	Colombia	Boyaca	Guateque	1320	4.9833	-73.4833	17.4	Wild AII	Wild K1
G26687	CIAT	Wild	Colombia	Cundinamarca	Macheta	1680	5.0833	-73.55	15.3	Wild AII	Wild K1
G26688	CIAT	Wild	Colombia	Cundinamarca	Tibirita	1520	5.0333	-73.5167	16.7	Wild AII	Wild K1
G26692	CIAT	Wild	Colombia	Boyaca	Sutatenza	1760	5.0333	-73.45	13.9	Wild AII	Wild K1
G26699	CIAT	Wild	Colombia	Boyaca	Garagoa	1430	5.1167	-73.3833	12.2	Wild AII	Wild K1
G26737	CIAT	Wild	Colombia	Cundinamarca	Tibirita	1480	5.0333	-73.5333	15	Wild AII	Wild K1
Acosta-13*	CICY	Wild	Mexico	Colima		560	21.445	-99.6355		Wild MI	Wild K2
Acosta-15*	CICY	Wild	Mexico	Morelos						Wild MI	Wild K2
Acosta-6*	CICY	Wild	Mexico	Guerrero		732	18.3447	-99.5427		Wild MI	Wild K2
Acosta-7*	CICY	Wild	Mexico	Guerrero		1372	17.5667	-99.4		Wild MI	Wild K2
G25228*	CIAT	Wild	Mexico	Nayarit	Compostela	600	20.9	-105.3833	6	Wild MI	Wild K2
G25230*	CIAT	Wild	Mexico	Colima	Manzanillo	3	19.05	-104.2333	7	Wild MI	Wild K2
G25231	CIAT	Wild	Mexico	Colima	Manzanillo	3	19.0333	-104.2167	6	Wild MI	Wild K2
G25233	CIAT	Wild	Belize	Cayo District	Listowel	40	17.2	-89.1333	9.8	Wild MI	Wild K2
G25284	CIAT	Wild	Guatemala	Jalapa	Jalapa	1362	14.6167	-89.9833	7	Wild MI	Wild K2
G25290	CIAT	Wild	Guatemala	Sacatepequez	Antigua Guatemala	1530	14.55	-90.7167	13	Wild MI	Wild K2
G25294C	CIAT	Weedy	Cuba	Matanzas	Varadero	3	23.1667	-81.2667	11.5	Wild MI	Wild K2
G25584A*	CIAT	Wild	Costa Rica	Guanacaste	Ca_as	100	10.7667	-85.4833	5	Wild MI	Wild K2
G25816	CIAT	Wild	Mexico	Yucatan	Izamal	30	20.9333	-89.0167	11	Wild MI	Wild K2
G25843	CIAT	Wild	Mexico	Morelos	Zacatepec	900	18.65	-99.2	12	Wild MI	Wild K2
G25911*	CIAT	Wild	Guatemala	Retalhuleu	Retalhuleu	240	14.5167	-91.6667	7.5	Wild MI	Wild K2
G25915	CIAT	Wild	Peru	Cajamarca	San Pablo	2020	-7.1833	-78.8333	16.6	Wild MI	Wild K3
G26358*	CIAT	Wild	Mexico	Morelos	Tlayacapan	1350	18.9	-98.9833	13	Wild MI	Wild K2
G26359*	CIAT	Wild	Mexico	Puebla	Tepeojuma		18.65	-98.4833	12	Wild MI	Wild K2
G26360*	CIAT	Wild	Mexico	Puebla	Atlixco	1600	18.7333	-98.45	12.3	Wild MI	Wild K2
G26469	CIAT	Wild	Ecuador	Imbabura	Otavalo	1390	0.2667	-78.55	18.5	Wild MI	Wild K2
G26515	CIAT	Wild	Colombia	Atlantico	Barranquilla	45	11.0333	-74.8833	11.2	Wild MI	Wild K2
G26517*	CIAT	Wild	Mexico	Guerrero	Ometepec	1140	16.6667	-98.45	8.2	Wild MI	Wild K2
G26518*	CIAT	Wild	Mexico	Guerrero	Ayutla De Los Libres	240	16.7833	-99.1833	7.4	Wild MI	Wild K2
G26533*	CIAT	Wild	Mexico	Chiapas	Tapachula	70	14.8333	-92.2667	9	Wild MI	Wild K2
G26606	CIAT	Wild	Ecuador	Chimborazo	Alausi	1550	-2.25	-78.9167	14.9	Wild MI	Wild K2
G26680	CIAT	Wild	Guatemala	Escuintla	Escuintla	520	14.35	-90.8	7.7	Wild MI	Wild K2
G26681	CIAT	Wild	Guatemala	Suchitepeque	Patulul	230	14.4	-91.1833	6.7	Wild MI	Wild K2
G26741	CIAT	Wild	Mexico	Oaxaca	Juquila	20	15.9667	-97.1167	6.8	Wild MI	Wild K2
G26742	CIAT	Wild	Mexico	Oaxaca	Juquila	40	15.9667	-97.4667	5.9	Wild MI	Wild K2

ID	Institution	Biological status	Country	Department	Municipality	Elevation (msnm)	Latitude	Longitude	Weight of 100 seeds (g)	PcoA results	Structure results
G26751A	CIAT	Wild	Ecuador	Pichincha	Atahualpa	1740	0.1333	-78.4167	25.4	Wild MI	Wild K2
JMC 1068	CICY	Wild	Mexico	Oaxaca						Wild MI	Wild K2
JMC 1069*	CICY	Wild	Mexico	Oaxaca		137	15.7672	-96.26		Wild MI	Wild K2
JMC 1074*	CICY	Wild	Mexico	Oaxaca		53	15.957444	-97.364389		Wild MI	Wild K2
JMC 1109	CICY	Wild	Mexico	Guanajuato		1810	21.0361	-104.343		Wild MI	Wild K2
JMC 1113*	CICY	Wild	Mexico	Nayarit		1491	22.4886	-105.377		Wild MI	Wild K2
JMC 1122*	CICY	Wild	Mexico	Sinaloa		60	24.871944	-107.471389		Wild MI	Wild K2
JMC 1123*	CICY	Wild	Mexico	Sinaloa		212	25.3772	-107.557		Wild MI	Wild K2
JMC 1124	CICY	Wild	Mexico	Sinaloa		79	23.1614	-106.091		Wild MI	Wild K2
JMC 1125	CICY	Wild	Mexico	Sinaloa		38	23.0464	-105.95		Wild MI	Wild K2
JMC 1127*	CICY	Wild	Mexico	Jalisco		140	20.8255	-105.089611		Wild MI	Wild K2
JMC 1129	CICY	Wild	Mexico	Jalisco		10	20.6467	-105.237		Wild MI	Wild K2
JMC 1133*	CICY	Wild	Mexico	Jalisco		50	19.752639	-105.2355		Wild MI	Wild K2
JMC 1135	CICY	Wild	Mexico	Jalisco		15	19.4242	-105.013		Wild MI	Wild K2
JMC 1142*	CICY	Wild	Mexico	Colima		10	19.1161	-104.102		Wild MI	Wild K2
JMC 1146	CICY	Wild	Mexico	Michoacàn		70	18.2533	-103.246		Wild MI	Wild K2
JMC 1149*	CICY	Wild	Mexico	Michoacàn						Wild MI	Wild K2
JMC 1150*	CICY	Wild	Mexico	Guerrero						Wild MI	Wild K2
JMC 1152*	CICY	Wild	Mexico	Guerrero		36	17.3703	-101.084		Wild MI	Wild K2
JMC 1154*	CICY	Wild	Mexico	Guerrero		10	17.1508	-100.492		Wild MI	Wild K2
JMC 1173*	CICY	Wild	Mexico	Tamaulipas		340	22.830639	-99.321444		Wild MI	Wild K2
JMC 1174*	CICY	Wild	Mexico	Tamaulipas		214	22.5514	-99.0783		Wild MI	Wild K2
ROL 224*	CICY	Wild	Mexico	Jalisco		1472	20.8347	-103.967		Wild MI	Wild K2
ROL 464*	CICY	Wild	Mexico	Jalisco		1317	20.6639	-103.723		Wild MI	Wild K2
Acosta-2	CICY	Wild	Mexico	Chiapas						Wild MII	Wild K1
Acosta-5	CICY	Wild	Mexico	Chiapas						Wild MII	Wild K1
G25222*	CIAT	Wild	Guatemala	Zacapa	Gualan	50	15.1	-89.3667	5	Wild MII	Wild K1
G25227	CIAT	Wild	Costa Rica	Heredia	Heredia	1100	10.0167	-84.1333	4	Wild MII	Wild K1
G25229*	CIAT	Wild	Mexico	Nayarit	Sayulita	500	20.8333	-105.4	4	Wild MII	Wild K1
G25234*	CIAT	Wild	Mexico	Yucatan	Oxcutzcab	10	20.3167	-89.4167	11.8	Wild MII	Wild K1
G25272D	CIAT	Wild	Guatemala	Santa Rosa	Cuilapa	893	14.2667	-90.3	6.1	Wild MII	Wild K1
G25273A*	CIAT	Wild	El Salvador	Ahuachapan	Ahuachapan	754	13.95	-89.8167	6.5	Wild MII	Wild K1
G25586	CIAT	Wild	Costa Rica	Guanacaste	Liberia	150	10.65	-85.4667	5	Wild MII	Wild K1
G25704	CIAT	Wild	Mexico	Jalisco	Zapopan	1390	20.8	-103.4	15.3	Wild MII	Wild K1

ID	Institution	Biological status	Country	Department	Municipality	Elevation (msnm)	Latitude	Longitude	Weight of 100 seeds (g)	PcoA results	Structure results
G25737*	CIAT	Wild	Mexico	Campeche	Hecelchakan	60	20.2167	-89.9167	12	Wild MII	Wild K1
G25762*	CIAT	Wild	Mexico	Campeche	Hopelchen	80	19.7667	-89.8667	12	Wild MII	Wild K1
G25785	CIAT	Wild	Mexico	Campeche	Hopelchen	140	20.05	-89.7333	12.2	Wild MII	Wild K1
G25819	CIAT	Wild	Colombia	Magdalena	Cienaga	580	10.8833	-74.05	10.9	Wild MII	Wild K1
G25850*	CIAT	Wild	Guatemala	Escuintla	Escuintla	820	14.4	-90.85	6	Wild MII	Wild K1
G25913	CIAT	Wild	Peru	Cajamarca	Santa Cruz	1810	-6.6167	-78.8667	12	Wild MII	Wild K1
G25963	CIAT	Wild	Costa Rica	San Jose	Tarbaca	1450	9.8	-84.1167	7	Wild MII	Wild K1
G25964	CIAT	Wild	Costa Rica	San Jose	Alajuelita	1520	9.8833	-84.1167	8	Wild MII	Wild K1
G25966*	CIAT	Wild	Costa Rica	Cartago	La Union	1320	9.9167	-84	7	Wild MII	Wild K1
G25970*	CIAT	Wild	Mexico	Morelos	Yautepec	1250	18.9	-99.0333	10	Wild MII	Wild K1
G25977	CIAT	Wild	Guatemala	Escuintla	Siquinala	490	14.3167	-90.95	7.8	Wild MII	Wild K1
G26309	CIAT	Wild	Colombia	Magdalena	Cienaga	50	10.7167	-74.1167	10	Wild MII	Wild K1
G26355*	CIAT	Wild	Mexico	Jalisco	Tepatitlan		21.05	-102.6667	13	Wild MII	Wild K1
G26519*	CIAT	Wild	Mexico	Guerrero	Ayutla De Los Libres	330	16.8667	-99.25	7.4	Wild MII	Wild K1
G26527	CIAT	Wild	Colombia	Cordoba	Sahagun	70	8.7167	-75.4667	10.2	Wild MII	Wild K1
G26531*	CIAT	Wild	Mexico	Chiapas	Ocosingo	890	16.9	-92.05	13.5	Wild MII	Wild K1
G26541	CIAT	Wild	Mexico	Puebla	Tehuacan	820	18.5	-97.2167	9.4	Wild MII	Wild K1
G26547	CIAT	Wild	Peru	Junin	Chanchamayo	980	-11.1333	-75.3667	12.5	Wild MII	Wild K1
G26628*	CIAT	Wild	Honduras	El Paraiso	El Paraiso	840	13.8333	-86.5333	7.5	Wild MII	Wild K1
G26629	CIAT	Wild	Honduras	Olancho	Catacamas	550	14.8167	-85.8333	7.8	Wild MII	Wild K1
G26631*	CIAT	Wild	Honduras	Yoro	Potrerillos	210	15.4	-86.45	8.4	Wild MII	Wild K1
G26632	CIAT	Wild	Honduras	Santa Barbara	Santa Barbara	260	15.3	-88.4833	7.4	Wild MII	Wild K1
G26633	CIAT	Wild	Honduras	Copan	Santa Rosa Copan	860	14.7667	-88.6833	8.4	Wild MII	Wild K1
G26634*	CIAT	Wild	Honduras	Francisco Morazan	Sabanagrande	980	13.8167	-87.2667	8.6	Wild MII	Wild K1
G26635	CIAT	Wild	Honduras	Francisco Morazan	Sabanagrande	840	13.7667	-87.1667	7.4	Wild MII	Wild K1
G26652*	CIAT	Wild	Guatemala	Guatemala	Mixco	1550	14.6167	-90.5667	11.6	Wild MII	Wild K1
G26679*	CIAT	Wild	Guatemala	Chimaltenango	El Tejar	1700	14.6333	-90.7667	12.3	Wild MII	Wild K1
G26683*	CIAT	Wild	Guatemala	Quezaltenango	Zunil	1750	14.7667	-91.5	11.1	Wild MII	Wild K1
G26685	CIAT	Wild	Argentina	Salta	San Martin	650	-22.95	-63.8667	13.5	Wild MII	Wild K1
G26686	CIAT	Wild	Guatemala	El Progreso	San Agustin Acasaguastlan	750	15.0333	-89.9333	13.2	Wild MII	Wild K1
G26721	CIAT	Wild	Ecuador	Azuay	Sta Isabel	470	-3.3333	-79.5667	14	Wild MII	Wild K1
G26731*	CIAT	Wild	Guatemala	El Progreso	San Agustin Acasaguastlan	270	14.9333	-89.95	6.8	Wild MII	Wild K1
G26733*	CIAT	Wild	Guatemala	Santa Rosa	Sn Rafael Las Flores	1380	14.45	-90.1333	8.2	Wild MII	Wild K1
G26734	CIAT	Wild	Guatemala	Escuintla	Sn Vicente Pacaya	1430	14.4	-90.6333	9	Wild MII	Wild K1
G26740	CIAT	Wild	Mexico	Oaxaca	Juchitan De Zaragoza	30	16.45	-95.0167	4.8	Wild MII	Wild K1
G26754*	CIAT	Wild	Mexico	Chiapas	Teopisca	1155	16.4833	-92.5167	7	Wild MII	Wild K1
G27292*	CIAT	Wild	Costa Rica	Guanacaste	Las Juntas	1180	10.3167	-84.85	6.7	Wild MII	Wild K1

ID	Institution	Biological status	Country	Department	Municipality	Elevation (msnm)	Latitude	Longitude	Weight of 100 seeds (g)	PcoA results	Structure results
G27298	CIAT	Wild	Colombia	Magdalena	Mamatoco	280	11.2667	-74.0833	4.5	Wild MII	Wild K1
G27345*	CIAT	Wild	Guatemala	San Marcos	Sn Rafael Pie Cuesta	900	14.9167	-91.9333	10	Wild MII	Wild K1
G27387*	CIAT	Wild	Costa Rica	Alajuela	Alajuela	930	10.0167	-84.2	9.2	Wild MII	Wild K1
G27441	CIAT	Wild	Colombia	Atlantico	Baranoa	120	10.7833	-74.9167	13.8	Wild MII	Wild K1
G27442	CIAT	Wild	Colombia	Atlantico	Baranoa	100	10.8	-74.9167	8	Wild MII	Wild K1
G27578	CIAT	Wild	Guatemala	Chimaltenango	Sn Jose Poaquil	1530	14.8833	-90.95	10.7	Wild MII	Wild K1
G27579*	CIAT	Wild	Guatemala	Guatemala	Fraijanes	1700	14.5	-90.4167	7.5	Wild MII	Wild K1
G27608*	CIAT	Wild	El Salvador	Santa Ana	Santa Ana	1775	13.8333	-89.6167	8.9	Wild MII	Wild K1
G27611	CIAT	Wild	Costa Rica	Puntarenas	Coto Brus	603	8.8333	-83.0833	10.3	Wild MII	Wild K1
G27613*	CIAT	Wild	Costa Rica	Heredia	Heredia	1149	9.9833	-84.1	6.9	Wild MII	Wild K1
G27619	CIAT	Wild	Mexico	Oaxaca	Matias Romero	700	16.8667	-95.0333	7.1	Wild MII	Wild K1
JMC 1012	CICY	Wild	Mexico	Yucatán		80	20.203056	-89.28778		Wild MII	Wild K1
JMC 1021	CICY	Wild	Mexico	Quintana Roo		11	21.102778	-87.337778		Wild MII	Wild K1
JMC 1063*	CICY	Wild	Mexico	Veracruz						Wild MII	Wild K1
JMC 1065	CICY	Wild	Mexico	Oaxaca		160	16.70175	-94.960778		Wild MII	Wild K1
JMC 1080	CICY	Wild	Mexico	Oaxaca						Wild MII	Wild K1
JMC 1081	CICY	Wild	Mexico	Oaxaca		90	17.938139	-96.171861		Wild MII	Wild K1
JMC 1082	CICY	Wild	Mexico	Oaxaca		133	17.7864	-96.3292		Wild MII	Wild K1
JMC 1089*	CICY	Wild	Guatemala	Petén		416	16.5231	-89.4197		Wild MII	Wild K1
JMC 1097	CICY	Wild	Guatemala	Petén		210	17.2258	-89.9539		Wild MII	Wild K1
JMC 1099	CICY	Wild	Guatemala	Petén		211	17.3761	-89.6333		Wild MII	Wild K1
JMC 1101	CICY	Wild	Guatemala	Petén		160	17.0164	-89.2944		Wild MII	Wild K1
JMC 1162	CICY	Wild	Mexico	Veracruz		300	18.434028	-95.157306		Wild MII	Wild K1
JMC 1168	CICY	Wild	Mexico	Veracruz		6	19.5997	-96.3983		Wild MII	Wild K1
JMC 1175	CICY	Wild	Mexico	Veracruz		102	20.4328	-97.3747		Wild MII	Wild K1
JMC 1388	CICY	Wild	Mexico	Chiapas			17.476111	-91.965167		Wild MII	Wild K1
JMC 1389	CICY	Wild	Mexico	Chiapas			16.608417	-93.428528		Wild MII	Wild K1
JMC 1390	CICY	Wild	Mexico	Chiapas			16.156722	-93.239389		Wild MII	Wild K1
JMC 1391*	CICY	Wild	Mexico	Chiapas			16.151972	-93.111806		Wild MII	Wild K1
JMC 1393	CICY	Wild	Mexico	Chiapas			16.657944	-93.014861		Wild MII	Wild admixed
JMC 1394	CICY	Wild	Mexico	Chiapas			16.277222	-92.440389		Wild MII	Wild K1
JMC 580	CICY	Wild	Mexico	Yucatàn		50	20.0623	-89.4159		Wild MII	Wild K1
JMC 609	CICY	Wild	Mexico	Yucatàn		21	20.209	-89.369		Wild MII	Wild K1
JMC 715	CICY	Wild	Mexico	Campeche						Wild MII	Wild K1

ID	Institution	Biological status	Country	Department	Municipality	Elevation (msnm)	Latitude	Longitude	Weight of 100 seeds (g)	PcoA results	Structure results
JMC 922	CICY	Wild	Mexico	Campeche		130	19.947647	-89.73415		Wild MII	Wild K1
