# Supplementary material for: Testing Domestication Scenarios of Lima Bean (Phaseolus lunatus L.) in Mesoamerica: Insights from Genome-Wide Genetic Markers
Source: Front Plant Sci. 2017 Sep 12;8:1551. doi: 10.3389/fpls.2017.01551 (PMC5601060; doi:10.3389/fpls.2017.01551)
Supplement: Supplementary file 2 [file Table2.PDF]

Supplementary table S2. Raw read number, mapped reads and SNPs genotyped per accession.

ID	No. of reads	unmapped reads	mapped 1 time	mapped more than 1 time	mapped total	%mapped	SNPs Genotyped
Acosta-13	2975023	997017	1109954	868052	1978006	0.66	4658
Acosta-15	2123607	690983	745047	687577	1432624	0.67	4621
Acosta-2	3215331	1063673	1230452	921206	2151658	0.67	4631
Acosta-5	1575247	490982	677936	406329	1084265	0.69	4269
Acosta-6	4804616	2114935	1560297	1129384	2689681	0.56	4612
Acosta-7	3092054	1086574	1247926	757554	2005480	0.65	4703
G25108	670459	239636	308439	122384	430823	0.64	4200
G25195	1264711	456014	604589	204108	808697	0.64	4630
G25216	1863912	697615	841539	324758	1166297	0.63	4669
G25222	2076165	861568	766494	448103	1214597	0.59	4354
G25224A	1271777	457555	623516	190706	814222	0.64	4596
G25227	2564135	938329	1150116	190707	1340823	0.52	4625
G25228	1272465	466332	621176	184957	806133	0.63	4588
G25229	1721613	659968	806745	254900	1061645	0.62	4000
G25230	2575653	899735	1196558	479360	1675918	0.65	4515
G25231	3753518	1359661	1674366	719491	2393857	0.64	4600
G25233	1158047	439216	533698	185133	718831	0.62	4633
G25234	982384	406437	438201	137746	575947	0.59	4384
G25267	3541206	1337426	1499309	704471	2203780	0.62	4579
G25272D	1963331	743584	798295	421452	1219747	0.62	4476
G25273A	1746211	678669	743926	323616	1067542	0.61	4546
G25277	983061	390107	460467	132487	592954	0.60	4573
G25278	1781334	640565	866166	274603	1140769	0.64	4692
G25284	1316870	484306	654859	177705	832564	0.63	4630
G25290	1204902	466631	524546	213725	738271	0.61	4604
G25290A	2103390	785354	902912	415124	1318036	0.63	4234
G25293	1520036	609406	608559	302071	910630	0.60	4288
G25294C	1342990	475098	654172	213720	867892	0.65	4656
G25303	1488358	546093	725389	216876	942265	0.63	4686
G25364	2913133	1200311	1098911	613911	1712822	0.59	4721
G25366	3093283	1166303	1267004	659976	1926980	0.62	4729
G25385B	1461755	552736	686951	222068	909019	0.62	4672
G25388	2260895	854072	1000760	406063	1406823	0.62	4220
G25391B	1644429	626793	687598	330038	1017636	0.62	4566
G25397	1955811	662712	921581	371518	1293099	0.66	4717
G25410	945742	351215	452614	141913	594527	0.63	4574

ID	No. of reads	unmapped reads	mapped 1 time	mapped more than 1 time	mapped total	%mapped	SNPs Genotyped
G25420	1189960	475684	542693	171583	714276	0.60	4584
G25540	1425537	578546	640624	206367	846991	0.59	4646
G25541	2325028	1070789	917732	336507	1254239	0.54	4685
G25542	2567801	1043528	1142081	382192	1524273	0.59	4732
G25543	2445170	935119	1072487	437564	1510051	0.62	4703
G25559	1746800	628986	731264	386550	1117814	0.64	4580
G25581	3300998	1231474	1442930	626594	2069524	0.63	4704
G25584A	1679908	625163	737024	317721	1054745	0.63	4205
G25586	2749053	1020737	1140483	587833	1728316	0.63	4687
G25596A	1285144	441638	605556	237950	843506	0.66	4576
G25597	1052954	414365	485551	153038	638589	0.61	4590
G25614	2499024	1005525	993526	499973	1493499	0.60	4706
G25700A	1197821	489171	518633	190017	708650	0.59	4492
G25704	1720131	612365	789984	317782	1107766	0.64	4635
G25705	1546383	568306	750705	227372	978077	0.63	4683
G25735	1612723	586759	768179	257785	1025964	0.64	4667
G25737	1764336	717358	667311	379667	1046978	0.59	4589
G25750	1839891	639218	949876	250797	1200673	0.65	4584
G25762	1316569	490226	656764	169579	826343	0.63	4403
G25766	2072780	797055	917052	358673	1275725	0.62	4523
G25770	1477094	567180	640570	269344	909914	0.62	4625
G25771	1867186	687426	858795	320965	1179760	0.63	3946
G25785	1811637	658642	896809	256186	1152995	0.64	4680
G25787	1024116	352539	524288	147289	671577	0.66	4494
G25816	2446002	1210288	961457	274257	1235714	0.51	4699
G25819	2098017	942502	778187	377328	1155515	0.55	4668
G25843	1668595	587817	803518	277260	1080778	0.65	4694
G25844	1771119	661930	860641	248548	1109189	0.63	4659
G25850	1679331	656362	738526	284443	1022969	0.61	4628
G25854	1503069	538974	638867	325228	964095	0.64	4654
G25908	2811902	958883	1425300	427719	1853019	0.66	4655
G25909	1340358	493335	645516	201507	847023	0.63	4603
G25910	2022966	771326	880749	370891	1251640	0.62	4401
G25911	2182453	789035	962162	431256	1393418	0.64	4464
G25913	1922042	706562	952339	263141	1215480	0.63	4629
G25915	1897670	707353	857070	333247	1190317	0.63	4657
G25916	1166488	433926	513304	219258	732562	0.63	3297
G25943	2069320	804766	941230	323324	1264554	0.61	3309

ID	No. of reads	unmapped reads	mapped 1 time	mapped more than 1 time	mapped total	%mapped	SNPs Genotyped
G25963	1742341	708797	702518	331026	1033544	0.59	4562
G25964	1825414	705955	756443	363016	1119459	0.61	4686
G25966	2284594	878925	853424	552245	1405669	0.62	4694
G25970	3145431	1117904	1364265	663262	2027527	0.64	4711
G25971	2682666	956867	1267815	457984	1725799	0.64	4719
G25974	1737909	650730	755734	331445	1087179	0.63	4683
G25977	3087945	1149680	1154573	783692	1938265	0.63	4547
G25981	1808668	644093	862449	302126	1164575	0.64	4325
G25987	1183968	452927	527145	203896	731041	0.62	4394
G26002	1740600	684153	728299	328148	1056447	0.61	4638
G26290	1651498	603404	776235	271859	1048094	0.63	4599
G26291	2123207	820207	963550	339450	1303000	0.61	4719
G26300	2085144	781344	995309	308491	1303800	0.63	4706
G26306	1753543	675019	793709	284815	1078524	0.62	4624
G26309	1792481	652051	914053	226377	1140430	0.64	4675
G26355	1634083	581349	844173	208561	1052734	0.64	4688
G26358	2789600	1031372	1159183	599045	1758228	0.63	4711
G26359	2092543	830608	806153	455782	1261935	0.60	4688
G26360	2375345	898594	975616	501135	1476751	0.62	4706
G26438	1924379	728377	880713	315289	1196002	0.62	4666
G26444	2953932	1237608	1181159	535165	1716324	0.58	4721
G26451	1368615	563408	588164	217043	805207	0.59	3529
G26459	1106150	410642	554135	141373	695508	0.63	4549
G26460	1659564	647904	686243	325417	1011660	0.61	4634
G26468	1746297	634135	813855	298307	1112162	0.64	4674
G26469	1683946	639502	794013	250431	1044444	0.62	4666
G26480	1506609	566143	731773	208693	940466	0.62	4574
G26490	3128245	1220661	1222746	684838	1907584	0.61	4720
G26505	2056590	768584	981185	306821	1288006	0.63	4697
G26512	2169622	886766	813472	469384	1282856	0.59	4671
G26513	2453336	968189	971728	513419	1485147	0.61	4708
G26515	1457291	541566	674813	240912	915725	0.63	4671
G26517	1206938	463957	475520	267461	742981	0.62	4507
G26518	2613507	964440	1017130	631937	1649067	0.63	4665
G26519	2134178	764114	979734	390330	1370064	0.64	3932
G26527	1792458	694754	765616	332088	1097704	0.61	4678
G26529	1606032	662295	612747	330990	943737	0.59	4654
G26531	1812055	706158	774767	331130	1105897	0.61	4644

ID	No. of reads	unmapped reads	mapped 1 time	mapped more than 1 time	mapped total	%mapped	SNPs Genotyped
G26533	1415426	524729	650941	239756	890697	0.63	4607
G26534	1694332	664324	785374	244634	1030008	0.61	4631
G26541	1559448	583803	731445	244200	975645	0.63	4616
G26542	2231982	868975	872054	490953	1363007	0.61	4707
G26545	1526998	583442	637674	305882	943556	0.62	4604
G26547	2290845	859164	951951	479730	1431681	0.62	4688
G26578	2373730	930305	1037687	405738	1443425	0.61	4676
G26606	1596836	577559	754465	264812	1019277	0.64	4690
G26608	1800964	681779	864910	254275	1119185	0.62	4675
G26609	1536700	576458	738553	221689	960242	0.62	4654
G26615	1717915	703046	636681	378188	1014869	0.59	4637
G26617	2868742	1113943	1332001	422798	1754799	0.61	4695
G26618	1672317	704550	659890	307877	967767	0.58	4639
G26628	2031327	719861	913185	398281	1311466	0.65	4690
G26629	390671	157938	164773	67960	232733	0.60	3644
G26630	1474883	537033	724163	213687	937850	0.64	4656
G26631	1549082	650404	610868	287810	898678	0.58	4612
G26632	918851	385276	347025	186550	533575	0.58	4228
G26633	1157455	479576	477947	199932	677879	0.59	4451
G26634	1603953	625519	726816	251618	978434	0.61	4561
G26635	1998421	763739	701240	533442	1234682	0.62	4606
G26647	1691751	698143	617670	375938	993608	0.59	4118
G26652	1741706	736013	747826	257867	1005693	0.58	4707
G26653	2006731	766471	891061	349199	1240260	0.62	4652
G26655	1840538	729556	756324	354658	1110982	0.60	4626
G26656	1594668	595436	712197	287035	999232	0.63	4556
G26659	2988933	1142751	1221673	624509	1846182	0.62	4676
G26672	1688153	632374	680801	374978	1055779	0.63	4600
G26679	1863743	748385	836788	278570	1115358	0.60	4698
G26680	1196978	443126	569202	184650	753852	0.63	4636
G26681	1641337	601258	757592	282487	1040079	0.63	4676
G26683	2275156	883816	1019758	371582	1391340	0.61	4703
G26684	1855768	742399	893500	219869	1113369	0.60	4675
G26685	1297385	482806	559665	254914	814579	0.63	4592
G26686	2165384	853698	890942	420744	1311686	0.61	4625
G26687	1701176	609574	782853	308749	1091602	0.64	4677
G26688	1925752	738465	786134	401153	1187287	0.62	4666
G26692	2304974	831203	1110561	363210	1473771	0.64	4694

ID	No. of reads	unmapped reads	mapped 1 time	mapped more than 1 time	mapped total	%mapped	SNPs Genotyped
G26699	2484795	987448	1083927	413420	1497347	0.60	4678
G26700	2106427	784373	915033	407021	1322054	0.63	3788
G26704	2100040	813505	1029640	256895	1286535	0.61	4668
G26706	1701025	638839	788371	273815	1062186	0.62	4694
G26714	2103517	804161	955022	344334	1299356	0.62	4720
G26721	2050289	765006	930396	354887	1285283	0.63	4636
G26731	1856794	788736	848184	219874	1068058	0.58	4560
G26732	1487085	557901	704466	224718	929184	0.62	4643
G26733	1178670	474695	516186	187789	703975	0.60	4625
G26734	2638435	977599	1093314	567522	1660836	0.63	4688
G26737	1406521	516488	631580	258453	890033	0.63	4573
G26740	2491364	948755	1098037	444572	1542609	0.62	4658
G26741	2574296	960795	1106779	506722	1613501	0.63	4683
G26742	1673591	599058	797279	277254	1074533	0.64	4682
G26751A	1559796	593262	753486	213048	966534	0.62	4594
G26753	1558253	623294	709990	224969	934959	0.60	4651
G26754	1044719	365537	491608	187574	679182	0.65	4562
G27289	1705070	628004	818028	259038	1077066	0.63	4672
G27292	2282177	926136	922196	433845	1356041	0.59	4683
G27298	1595575	612081	661745	321749	983494	0.62	4614
G27337	1586094	589360	760225	236509	996734	0.63	4618
G27338	1488996	542522	643867	302607	946474	0.64	3968
G27345	1488799	621545	666399	200855	867254	0.58	4671
G27350A	1143291	461434	484637	197220	681857	0.60	4625
G27358	2281152	924713	993248	363191	1356439	0.59	4720
G27381	1058448	434174	455482	168792	624274	0.59	4513
G27387	2534453	938745	924829	670879	1595708	0.63	4680
G27388	2439518	951551	1081869	406098	1487967	0.61	4723
G27399	1581933	601188	636476	344269	980745	0.62	4584
G27422	2468643	931945	1029884	506814	1536698	0.62	4679
G27429	2615979	1011108	1041512	563359	1604871	0.61	4708
G27441	2272265	894982	944552	432731	1377283	0.61	4671
G27442	2064343	795196	806099	463048	1269147	0.61	4661
G27445	2880698	1188071	1196176	496451	1692627	0.59	4623
G27448	2541704	1054033	1014319	473352	1487671	0.59	4634
G27455	3008707	1159549	1226746	622412	1849158	0.61	4736
G27578	3436357	1168201	1448158	819998	2268156	0.66	4719
G27579	1760605	778042	656161	326402	982563	0.56	4623

ID	No. of reads	unmapped reads	mapped 1 time	mapped more than 1 time	mapped total	%mapped	SNPs Genotyped
G27608	2461478	979453	975619	506406	1482025	0.60	4688
G27611	2552385	977140	1086816	488429	1575245	0.62	4688
G27613	2128013	897338	885376	345299	1230675	0.58	4655
G27619	731339	320568	294071	116700	410771	0.56	4181
JMC 1012	2770232	848230	1267396	654606	1922002	0.69	4473
JMC 1021	2735824	827785	1103986	804053	1908039	0.70	4612
JMC 1024	2574257	831140	1169724	573393	1743117	0.68	4721
JMC 1035	2099285	646117	1105954	347214	1453168	0.69	4721
JMC 1048	1994249	661670	908490	424089	1332579	0.67	4705
JMC 1061	3554756	1262627	1225375	1066754	2292129	0.64	4694
JMC 1063	1973171	626486	997178	349507	1346685	0.68	4681
JMC 1065	1690168	568795	740380	380993	1121373	0.66	4641
JMC 1068	3233493	1062017	1479565	691911	2171476	0.67	4597
JMC 1069	3875928	1390849	1563962	921117	2485079	0.64	4567
JMC 1074	2949966	1084406	1195881	669679	1865560	0.63	4655
JMC 1080	2529800	866830	1170882	492088	1662970	0.66	4666
JMC 1081	1591029	593675	721325	276029	997354	0.63	4583
JMC 1082	1997863	652111	750299	595453	1345752	0.67	4454
JMC 1089	3219434	1040202	1448457	730775	2179232	0.68	4710
JMC 1097	1779594	540767	657681	581146	1238827	0.70	4521
JMC 1099	1115966	343460	548413	224093	772506	0.69	4585
JMC 1101	2007137	582188	1059769	365180	1424949	0.71	4685
JMC 1104	1785356	525925	990911	268520	1259431	0.71	4721
JMC 1105	3179722	1751884	1002832	425006	1427838	0.45	4715
JMC 1106	3220493	1237474	1407427	575592	1983019	0.62	4731
JMC 1108	2140401	592099	1163148	385154	1548302	0.72	4181
JMC 1109	2800434	809442	1189670	801322	1990992	0.71	4525
JMC 111	2423345	714328	1342311	366706	1709017	0.71	4739
JMC 1113	2678863	862602	915085	901176	1816261	0.68	4615
JMC 112	2997302	983858	1274403	739041	2013444	0.67	4723
JMC 1122	2513250	774445	1008140	730665	1738805	0.69	4685
JMC 1123	2231201	697754	951103	582344	1533447	0.69	4683
JMC 1124	3133634	1003137	1225481	905016	2130497	0.68	4605
JMC 1125	2655134	902620	885118	867396	1752514	0.66	4658
JMC 1127	2057272	871435	841113	344724	1185837	0.58	4495
JMC 1129	1481233	527202	620128	333903	954031	0.64	4437
JMC 1133	2276778	655846	973790	647142	1620932	0.71	4675
JMC 1135	2014552	603702	831964	578886	1410850	0.70	4527

ID	No. of reads	unmapped reads	mapped 1 time	mapped more than 1 time	mapped total	%mapped	SNPs Genotyped
JMC 1142	2222640	997156	785464	440020	1225484	0.55	4677
JMC 1146	2130654	667225	1046934	416495	1463429	0.69	4503
JMC 1149	2484608	874220	1059058	551330	1610388	0.65	4517
JMC 1150	2099781	807292	852556	439933	1292489	0.62	4522
JMC 1152	2457403	850685	1074423	532295	1606718	0.65	4626
JMC 1154	2638647	975573	1168095	494979	1663074	0.63	4670
JMC 1162	3714039	1539164	1384443	790432	2174875	0.59	4669
JMC 1168	1865044	661005	755893	448146	1204039	0.65	4431
JMC 1173	3983328	1365167	1853931	764230	2618161	0.66	4696
JMC 1174	2291163	944783	961363	385017	1346380	0.59	4594
JMC 1175	2682485	935842	1126009	620634	1746643	0.65	4643
JMC 1179	2461697	807353	1082719	571625	1654344	0.67	4699
JMC 1186	5161179	4434964	506189	220026	726215	0.14	4581
JMC 1197	2591029	897809	987997	705223	1693220	0.65	4714
JMC 1199	1803698	542099	816647	444952	1261599	0.70	4631
JMC 1200	1609588	547895	819212	242481	1061693	0.66	4716
JMC 1262	1929983	648921	836784	444278	1281062	0.66	4703
JMC 1265	2340365	750916	842741	746708	1589449	0.68	4667
JMC 1277	2056392	659732	775695	620965	1396660	0.68	4688
JMC 1284	3196289	1041130	1367981	787178	2155159	0.67	4712
JMC 1288	2417195	732434	1122144	562617	1684761	0.70	4719
JMC 13	2874311	1118523	1206874	548914	1755788	0.61	4709
JMC 1312	1830936	559067	715984	555885	1271869	0.69	4682
JMC 1325	3070037	996262	1400872	672903	2073775	0.68	4714
JMC 1335	1876862	702411	678619	495832	1174451	0.63	4674
JMC 1377	2962362	964978	1490334	507050	1997384	0.67	4717
JMC 1378-A	1820810	542930	876724	401156	1277880	0.70	4708
JMC 1381-A	2490018	799281	1015234	675503	1690737	0.68	4716
JMC 1382-B	2812249	914671	1223366	674212	1897578	0.67	4725
JMC 1386	2792789	1089654	1085301	617834	1703135	0.61	4711
JMC 1388	2867169	920978	1347428	598763	1946191	0.68	4587
JMC 1389	3313113	1106903	1464709	741501	2206210	0.67	4567
JMC 1390	2105731	708172	833135	564424	1397559	0.66	4610
JMC 1391	2254565	645327	1156013	453225	1609238	0.71	4573
JMC 1393	2714904	1029020	1106489	579395	1685884	0.62	4613
JMC 1394	2064230	809659	860556	394015	1254571	0.61	4679

ID	No. of reads	unmapped reads	mapped 1 time	mapped more than 1 time	mapped total	%mapped	SNPs Genotyped
JMC 580	2288945	764105	965048	559792	1524840	0.67	4552
JMC 609	2123568	630428	1033688	459452	1493140	0.70	4358
JMC 715	2765786	935661	1164843	665282	1830125	0.66	4352
JMC 9	1976121	669680	1025423	281018	1306441	0.66	4722
JMC 922	2769275	1033579	1301176	434520	1735696	0.63	4687
JMC 995	4153773	1639916	1478954	1034903	2513857	0.61	4708
ROL 224	2462894	657301	1228908	576685	1805593	0.73	4710
ROL 464	2021758	617573	842753	561432	1404185	0.69	4661
**Total**	**565.386.739**	**210.685.631**	**242.489.038**	**112.212.070**	**354.701.108**	**0.63**	
**Average**	**2.094.025**	**780.317**	**898.108**	**414.545**	**1.312.652**		**4588.54**
