# Supplementary material for: Testing Domestication Scenarios of Lima Bean (Phaseolus lunatus L.) in Mesoamerica: Insights from Genome-Wide Genetic Markers
Source: Front Plant Sci. 2017 Sep 12;8:1551. doi: 10.3389/fpls.2017.01551 (PMC5601060; doi:10.3389/fpls.2017.01551)
Supplement: Supplementary file 3 [file Table3.PDF]

Supplementary table S3. Conflicts in gene pool classification (Andean versus Mesoamerican) based on ITS polymorphisms (Serrano-Serrano et al., 2012) and GBS data (present study) for a set of 24 wild and domesticated accessions of Lima bean.

ID	Institution	Biological status	Country	Department	Municipality	Weight of 100 seeds (g)	Genepool (ITS)	Gene pool GBS
G25981	CIAT	Domesticated	Bolivia	Chuquisaca	Oropeza	121.5	AI	MI
G25410	CIAT	Domesticated	Ecuador	Los Rios	Quevedo	32	AI	MI
G26480	CIAT	Domesticated	Ecuador	Imbabura	Ibarra	64.4	AI	MI
G25909	CIAT	Domesticated	Peru	Cajamarca	San Miguel	79	AI	MI
G27337	CIAT	Domesticated	Bolivia	Chuquisaca	Tomina	90.3	AI	MII
G26659	CIAT	Domesticated	Ecuador	Imbabura	Ibarra	111.5	AI	MII
G26672	CIAT	Domesticated	Ecuador	Carchi	Mira	75.4	AI	MII
G26290	CIAT	Domesticated	Argentina	Formosa	Formosa	40	MI	AI
G26438	CIAT	Domesticated	Costa Rica	Cartago	Turrialba	53	MI	AI
G25277	CIAT	Domesticated	El Salvador	Cuscatlan	Cuscatlan	40	MI	AI
G25771	CIAT	Domesticated	Mexico	Campeche	Hecelchakan	47	MI	AI
G25108	CIAT	Domesticated	Brazil	Minas Gerais	Vicosa	83	MII	AI
G26469	CIAT	Wild	Ecuador	Imbabura	Otavalo	18.5	AI	MI
G26751A	CIAT	Wild	Ecuador	Pichincha	Atahualpa	25.4	AI	MI
G26721	CIAT	Wild	Ecuador	Azuay	Sta Isabel	14	AI	MII
G25913	CIAT	Wild	Peru	Cajamarca	Santa Cruz	12	AI	MII
G26704	CIAT	Wild	Colombia	Caldas	Aguadas	17	MII	AI
G25844	CIAT	Wild	Guatemala	Sacatepequez	Santa Maria De Jesus	8.8	MII	AI
G26653	CIAT	Wild	Guatemala	Huehuetenang o	Sta. Ana Huista	11.6	MII	AI
G26655	CIAT	Wild	Guatemala	Sacatepequez	Alotenango	8.3	MII	AI
G26684	CIAT	Wild	Guatemala	Solola	Panajachel	11.3	MII	AI
G26732	CIAT	Wild	Guatemala	Jalapa	Sn Luis Jilotepeque	10.6	MII	AI
G26630	CIAT	Wild	Honduras	Olancho	San Francisco De La Paz	8.7	MII	AI
G26753	CIAT	Wild	Mexico	Chiapas	Venustiano Carranza	8	MII	AI
