# Supplementary material for: Testing Domestication Scenarios of Lima Bean (Phaseolus lunatus L.) in Mesoamerica: Insights from Genome-Wide Genetic Markers
Source: Front Plant Sci. 2017 Sep 12;8:1551. doi: 10.3389/fpls.2017.01551 (PMC5601060; doi:10.3389/fpls.2017.01551)
Supplement: Supplementary file 4 [file Table4.PDF]

Supplementary table S4. Prior-model combination statistics for scenario 1 and 2 based on 11 summary statistics of genetic diversity, genetic divergence and admixture rate.

Summary statistics	Observed value	Scenario 1	Scenario 2
HMO_1_1	0.1396	0.1194	0.1151
HMO_1_2	0.0993	0.2653	0.2583
HMO_1_3	0.2206	0.7984	0.1423
HMO_1_4	0.2059	0.2767	0.277
FMO_1_1&2	0.1188	0.5905	0.574
FMO_1_1&3	0.4284	0.8667	0.9839 (*)
FMO_1_1&4	0.4363	0.9267	0.9102
FMO_1_2&3	0.4868	0.8761	0.9900 (*)
FMO_1_2&4	0.4896	0.9481	0.9311
FMO_1_3&4	0.0308	0.0444 (*)	0.1395
AMO_1_3&2&4	0.1738	0.1635	0.0429 (*)

Values indicate for each summary statistics the proportion of simulated data sets which have a value below the observed one. Asterisks indicate statistically significant values. HMO: genetic diversity, FMO: divergence measured as F_{ST}, AMO: admixture rate.
