# Supplementary material for: Testing Domestication Scenarios of Lima Bean (Phaseolus lunatus L.) in Mesoamerica: Insights from Genome-Wide Genetic Markers
Source: Front Plant Sci. 2017 Sep 12;8:1551. doi: 10.3389/fpls.2017.01551 (PMC5601060; doi:10.3389/fpls.2017.01551)
Supplement: Supplementary file 5 [file Table5.PDF]

Supplementary table S5. Model comparison for scenarios 1 and 2 under the direct approach and the logistic regression approach.

Direct approach		
closest	scenario 1	scenario 2
50	0.6200 [0.1945,1.0000]	0.3800 [0.0000,0.8055]
100	0.6500 [0.2319,1.0000]	0.3500 [0.0000,0.7681]
150	0.7067 [0.3076,1.0000]	0.2933 [0.0000,0.6924]
200	0.7250 [0.3336,1.0000]	0.2750 [0.0000,0.6664]
250	0.7080 [0.3095,1.0000]	0.2920 [0.0000,0.6905]
300	0.7267 [0.3360,1.0000]	0.2733 [0.0000,0.6640]
350	0.7257 [0.3346,1.0000]	0.2743 [0.0000,0.6654]
400	0.7250 [0.3336,1.0000]	0.2750 [0.0000,0.6664]
450	0.7244 [0.3328,1.0000]	0.2756 [0.0000,0.6672]
500	0.7200 [0.3264,1.0000]	0.2800 [0.0000,0.6736]
Logistic approach		
N	scenario 1	scenario 2
1000	0.0001 [0.0000,0.0041]	0.9999 [0.9959,1.0000]
2000	0.0000 [0.0000,0.0007]	1.0000 [0.9993,1.0000]
3000	0.0000 [0.0000,0.0003]	1.0000 [0.9997,1.0000]
4000	0.0000 [0.0000,0.0002]	1.0000 [0.9998,1.0000]
5000	0.0000 [0.0000,0.0001]	1.0000 [0.9999,1.0000]
6000	0.0000 [0.0000,0.0001]	1.0000 [0.9999,1.0000]
7000	0.0000 [0.0000,0.0001]	1.0000 [0.9999,1.0000]
8000	0.0000 [0.0000,0.0001]	1.0000 [0.9999,1.0000]
9000	0.0000 [0.0000,0.0001]	1.0000 [0.9999,1.0000]
10000	0.0000 [0.0000,0.0001]	1.0000 [0.9999,1.0000]
