# Supplementary material for: Testing Domestication Scenarios of Lima Bean (Phaseolus lunatus L.) in Mesoamerica: Insights from Genome-Wide Genetic Markers
Source: Front Plant Sci. 2017 Sep 12;8:1551. doi: 10.3389/fpls.2017.01551 (PMC5601060; doi:10.3389/fpls.2017.01551)
Supplement: Supplementary file 6 [file Table6.PDF]

Supplementary table S6. Bias and error measures in parameter estimation of the ABC approach based on the posterior distribution of scenarios 1 and 2. Values between parentheses correspond to accuracy measures without taking into account the genetic information. Accuracy measures are: mean relative bias, the square Root of the Relative Mean Integrated Square Error (RRMISE) and the Relative Mean Absolute Deviation (RmeanAD).

Scenario 1					
Parameter	True values	Means	Mean relative Bias	RRMISE	RmeanAD
N1	1.24E+05	2.50E+05 (9.98E+05)	1.060 (7.688)	1.235 (9.714)	1.079 (7.748)
N2	6.44E+05	9.52E+05 (1.00E+06)	10.045 (10.549)	69.010 (71.640)	10.410 (10.870)
N3	1.36E+06	9.85E+05 (9.99E+05)	0.221 (0.264)	2.913 (3.135)	0.909 (0.943)
N4	2.01E+05	4.11E+05 (1.00E+06)	1.088 (4.461)	1.290 (5.892)	1.105 (4.560)
t1	8.28E+03	5.06E+03 (4.66E+03)	-0.341 (-0.382)	0.501 (0.543)	0.430 (0.476)
db2	1.12E+03	1.35E+03 (2.00E+03)	0.213 (0.809)	0.406 (0.982)	0.261 (0.822)
N3b	4.61E+03	4.39E+03 (2.50E+03)	-0.043 (-0.456)	0.126 (0.555)	0.096 (0.467)
t2	9.72E+03	7.77E+03 (7.33E+03)	-0.199 (-0.243)	0.274 (0.315)	0.208 (0.251)
db1	2.74E+03	2.11E+03 (2.00E+03)	-0.210 (-0.253)	0.329 (0.360)	0.267 (0.302)
N2b	3.28E+02	3.32E+02 (2.50E+03)	0.496 (13.210)	2.664 (20.116)	0.911 (13.273)
t3	3.72E+05	7.05E+05 (6.48E+05)	0.980 (0.833)	1.156 (1.068)	0.996 (0.870)
Scenario 2					
Parameter	True values	Means	Mean relative bias	RRMISE	RmeanAD
N1	2.04E+05	2.77E+05 (1.00E+06)	0.473 (4.371)	0.836 (5.785)	0.593 (4.473)
N2	1.37E+06	9.90E+05 (1.00E+06)	0.427 (0.431)	4.668 (4.562)	1.126 (1.113)
N3	5.70E+05	7.55E+05 (1.00E+06)	1.708 (3.530)	4.361 (7.804)	2.077 (3.816)
N4	3.47E+05	5.05E+05 (9.99E+05)	0.579 (2.251)	0.993 (3.298)	0.691 (2.426)
t1	6.28E+03	5.02E+03 (4.66E+03)	-0.084 (-0.132)	0.538 (0.570)	0.412 (0.450)
ra	9.55E-01	9.54E-01 (4.99E-01)	-0.000 (-0.477)	0.024 (0.564)	0.015 (0.479)
t2	8.08E+03	7.61E+03 (7.32E+03)	-0.003 (-0.038)	0.375 (0.390)	0.262 (0.280)
db1	2.88E+03	2.16E+03 (2.00E+03)	-0.245 (-0.299)	0.322 (0.370)	0.263 (0.314)
N2b	5.41E+02	5.16E+02 (2.51E+03)	0.270 (7.693)	2.234 (13.764)	0.760 (7.798)
t3	5.87E+05	7.42E+05 (6.50E+05)	0.402 (0.235)	0.702 (0.619)	0.524 (0.460)
