# Supplementary material for: Testing Domestication Scenarios of Lima Bean (Phaseolus lunatus L.) in Mesoamerica: Insights from Genome-Wide Genetic Markers
Source: Front Plant Sci. 2017 Sep 12;8:1551. doi: 10.3389/fpls.2017.01551 (PMC5601060; doi:10.3389/fpls.2017.01551)
Supplement: Supplementary file 7 [file Table7.PDF]

Supplementary table S7. F_{ST} outlier loci detected with the software Bayescan.

**Chr**	**Position**	**Gene**	**Kind of change**	**Annotation**	**GO Ontology**
1	39908571	Phvul.001G146000	missense	SLOW GROWTH 1	GO:0005739 mitochondrion
3	38852806	Phvul.003G176700	missense	Histone deacetylase 15	GO:0004407 histone deacetylase activity<br>GO:0005622 intracellular<br>GO:0005634 nucleus<br>GO:0008270 zinc ion binding<br>GO:0009294 DNA mediated transformation<br>GO:0016575 histone deacetylation<br>GO:0048573 photoperiodism, flowering
6	19813008	Phvul.006G079300	Synonymous	High-level expression of sugar-inducible gene 2	GO:0003677 DNA binding<br>GO:0003700 sequence-specific DNA binding transcription factor activity<br>GO:0005515 protein binding<br>GO:0005634 nucleus<br>GO:0005739 mitochondrion<br>GO:0006355 regulation of transcription, DNA-dependent<br>GO:0008270 zinc ion binding<br>GO:0009737 response to abscisic acid stimulus<br>GO:0009744 response to sucrose stimulus<br>GO:0010030 positive regulation of seed germination<br>GO:0045892 negative regulation of transcription, DNA-dependent<br>GO:2000034 regulation of seed maturation
9	10392287	Phvul.009G056400	3'UTR	Brassinosteroid signalling positive regulator (BZR1) family protein	GO:0003677 DNA binding<br>GO:0003700 sequence-specific DNA binding transcription factor activity<br>GO:0005515 protein binding<br>GO:0005634 nucleus<br>GO:0005829 cytosol<br>GO:0006355 regulation of transcription, DNA-dependent<br>GO:0009742 brassinosteroid mediated signaling pathway
9	12646538	Phvul.009G077400	Synonymous	Glycine decarboxylase P-protein 2	GO:0005960 glycine cleavage complex
