# Supplementary material for: Testing Domestication Scenarios of Lima Bean (Phaseolus lunatus L.) in Mesoamerica: Insights from Genome-Wide Genetic Markers
Source: Front Plant Sci. 2017 Sep 12;8:1551. doi: 10.3389/fpls.2017.01551 (PMC5601060; doi:10.3389/fpls.2017.01551)
Supplement: Supplementary file 8 [file Table8.PDF]

Supplementary table S8. F_{ST} outlier loci detected with the software Arlequin.

Chr	Position	He	FST	Gene	Kind of change	Annotation	GO Ontology
1	2213577	0.32	0.56	Phvul.001G024800	intron	Protein of unknown function (DUF3223)	GO:0009507 chloroplast<br>GO:0042631 cellular response to water deprivation
2	25060689	0.33	0.58	intergenic	intergenic		
2	30725536	0.35	0.64	Phvul.002G165600	missense	ALBINA 1. In Arabidopsis it encodes the CHLD subunit of the Mg-chelatase enzyme involved in chlorophyll biosynthesis. Lines carrying recessive mutations of this locus are white and seedling lethal.	GO:0000166 nucleotide binding<br>GO:0005524 ATP binding<br>GO:0009507 chloroplast<br>GO:0009534 chloroplast thylakoid<br>GO:0009570 chloroplast stroma<br>GO:0009690 cytokinin metabolic process<br>GO:0010007 magnesium chelatase complex<br>GO:0015979 photosynthesis<br>GO:0015995 chlorophyll biosynthetic process<br>GO:0016851 magnesium chelatase activity<br>GO:0017111 nucleoside-triphosphatase activity<br>GO:0019288 isopentenyl diphosphate biosynthetic process, mevalonate-independent pathway
2	42898862	0.44	0.73	Phvul.002G262700	synonymous	-	GO:0005739 mitochondrion<br>GO:0006486 protein glycosylation<br>GO:0031305 integral to mitochondrial inner membrane
2	42898868	0.46	0.75	Phvul.002G262700	synonymous	-	-
2	45969626	0.40	0.70	Phvul.002G296300	synonymous	COP1-interactive protein 1	GO:0005515 protein binding<br>GO:0005634 nucleus<br>GO:0005773 vacuole<br>GO:0005774 vacuolar membrane<br>GO:0005856 cytoskeleton<br>GO:0009507 chloroplast<br>GO:0042306 regulation of protein import into nucleus
3	28193510	0.38	0.66	Phvul.003G111900	synonymous	calmodulin binding;transcription regulators	GO:0003677 DNA binding<br>GO:0005516 calmodulin binding<br>GO:0005634 nucleus<br>GO:0006355 regulation of transcription, DNA-dependent
3	33524887	0.38	0.70	Phvul.003G141400	synonymous	Pathogenesis related homeodomain protein A	GO:0003700 sequence-specific DNA binding transcription factor activity<br>GO:0005515 protein binding<br>GO:0005634 nucleus<br>GO:0006355 regulation of transcription, DNA-dependent<br>GO:0008270 zinc ion binding<br>GO:0009733 response to auxin stimulus<br>GO:0043565 sequence-specific DNA binding<br>GO:0045893 positive regulation of transcription, DNA-dependent
3	37327632	0.29	0.61	Phvul.003G164900	intron	SPX domain gene 2	GO:0003674 molecular_function<br>GO:0005634 nucleus<br>GO:0006817 phosphate ion transport<br>GO:0016036 cellular response to phosphate starvation<br>GO:0019375 galactolipid biosynthetic process<br>GO:0045892 negative regulation of transcription, DNA-dependent
3	39543766	0.31	0.59	Phvul.003G183500	synonymous	Galactose oxidase/kelch repeat superfamily protein	

3	40801114	0.39	0.70	Phvul.003G195400	intron	ARM repeat superfamily protein	GO:0005634 nucleus<br>GO:0006606 protein import into nucleus<br>GO:0043484 regulation of RNA splicing
3	44813326	0.39	0.66	intergenic	intergenic		
4	29183182	0.12	0.37	Phvul.004G099700	missense	disease resistance family protein / LRR family protein	GO:0005618 cell wall<br>GO:0006952 defense response<br>GO:0007165 signal transduction<br>GO:0050832 defense response to fungus
4	29183190	0.12	0.37	Phvul.004G099700	synonymous	disease resistance family protein / LRR family protein	
5	40261864	0.35	0.65	Phvul.005G180700	synonymous	H(+)-ATPase 9	GO:0000166 nucleotide binding<br>GO:0003824 catalytic activity<br>GO:0005524 ATP binding<br>GO:0005774 vacuolar membrane<br>GO:0005886 plasma membrane<br>GO:0006200 ATP catabolic process<br>GO:0006754 ATP biosynthetic process<br>GO:0006812 cation transport<br>GO:0008152 metabolic process<br>GO:0008553 hydrogen-exporting ATPase activity, phosphorylative mechanism<br>GO:0015662 ATPase activity, coupled to transmembrane movement of ions, phosphorylative mechanism<br>GO:0015992 proton transport<br>GO:0016020 membrane<br>GO:0016021 integral to membrane<br>GO:0016887 ATPase activity<br>GO:0046872 metal ion binding
6	20681924	0.31	0.56	Phvul.006G088200	intron	Plant invertase/pectin methylesterase inhibitor superfamily	GO:0004857 enzyme inhibitor activity<br>GO:0005618 cell wall<br>GO:0005634 nucleus<br>GO:0009505 plant-type cell wall<br>GO:0030599 pectinesterase activity<br>GO:0042545 cell wall modification
6	21912484	0.40	0.73	Phvul.006G102400	synonymous	ankyrin repeat family protein	GO:0000741 karyogamy<br>GO:0005575 cellular_component<br>GO:0006457 protein folding<br>GO:0009408 response to heat<br>GO:0009560 embryo sac egg cell differentiation<br>GO:0009644 response to high light intensity<br>GO:0034976 response to endoplasmic reticulum stress<br>GO:0042542 response to hydrogen peroxide<br>GO:0051510 regulation of unidimensional cell growth
7	48896191	0.41	0.73	Phvul.007G249800	missense		
8	18748661	0.31	0.56	Phvul.008G126700	intron	Pentatricopeptide repeat (PPR) superfamily protein	GO:0005739 mitochondrion<br>GO:0009507 chloroplast<br>GO:0009553 embryo sac development<br>GO:0009555 pollen development<br>GO:0009790 embryo development<br>GO:0009793 embryo development ending in seed dormancy<br>GO:0019843 rRNA binding<br>GO:0048868 pollen tube development

9	12753513	0.32	0.60	Phvul.009G078300	3'UTR	heat shock transcription factor A2	GO:0001666 response to hypoxia<br>GO:0003677 DNA binding<br>GO:0003700 sequence-specific DNA binding transcription factor activity<br>GO:0005515 protein binding<br>GO:0005634 nucleus<br>GO:0006355 regulation of transcription, DNA-dependent<br>GO:0006457 protein folding<br>GO:0009407 toxin catabolic process<br>GO:0009408 response to heat<br>GO:0009507 chloroplast<br>GO:0009644 response to high light intensity<br>GO:0010200 response to chitin<br>GO:0010286 heat acclimation<br>GO:0034605 cellular response to heat<br>GO:0034620 cellular response to unfolded protein<br>GO:0034976 response to endoplasmic reticulum stress<br>GO:0042542 response to hydrogen peroxide<br>GO:0043565 sequence-specific DNA binding<br>GO:0045893 positive regulation of transcription, DNA-dependent<br>GO:0071456 cellular response to hypoxia
11	10186120	0.30	0.57	Phvul.011G097900	synonymous	Late embryogenesis abundant (LEA) hydroxyproline-rich glycoprotein family	
