# Supplementary material for: Testing Domestication Scenarios of Lima Bean (Phaseolus lunatus L.) in Mesoamerica: Insights from Genome-Wide Genetic Markers
Source: Front Plant Sci. 2017 Sep 12;8:1551. doi: 10.3389/fpls.2017.01551 (PMC5601060; doi:10.3389/fpls.2017.01551)
Supplement: Supplementary file 9 [file Table9.PDF]

Supplementary table S9. Domestication candidate genes (150 in total) within regions in high LD among wild and domesticated Lima beans detected with the software varLD. Candidate genes reported by Schmutz et al. (2014) were taken as reference. 20 candidate genes fell within the LD region in chromosome 2, 82 candidate genes fell within the LD region in chromosome 3, 24 candidate genes fell within the LD region in chromosome 7 and 24 candidate genes fell within the LD region in chromosome 9, for a total of 150 candidate genes.

**Gene**	**Chrom.**	**Start**	**End**	**Top *A. thaliana* hit (taken from Schmutz et al. (2014))**	**Gene symbol (taken from Schmutz et al. (2014))**	**Description(taken from Schmutz et al. (2014))**
Phvul.002G024600	Chr02	2,579,797	2,594,279	AT2G23740		nucleic acid binding
Phvul.002G025300	Chr02	2,717,880	2,721,085	AT3G26300	CYP71B34	cytochrome P450, family 71, subfamily B, polypeptide 34
Phvul.002G025700	Chr02	2,760,341	2,762,191	AT2G23770		protein kinase family protein / peptidoglycan-binding LysM domain-containing protein
Phvul.002G027700	Chr02	2,930,089	2,933,617	AT2G33840		Tyrosyl-tRNA synthetase, class Ib, bacterial/mitochondrial
Phvul.002G027800	Chr02	2,935,049	2,937,861	AT4G38150		Pentatricopeptide repeat (PPR) superfamily protein
Phvul.002G028800	Chr02	3,002,480	3,007,507	AT5G65650		Protein of unknown function (DUF1195)
Phvul.002G029300	Chr02	3,046,585	3,051,312	AT3G50670	U1-70K, U1SNRNP	U1 small nuclear ribonucleoprotein-70K
Phvul.002G030900	Chr02	3,194,329	3,197,976	AT4G36610		calcium-dependent protein kinase 33
Phvul.002G031300	Chr02	3,219,506	3,221,207	AT4G39250	ATRL1, RSM2, RL1	expansin 11
Phvul.002G031900	Chr02	3,260,870	3,264,028	AT5G66530		basic helix-loop-helix (bHLH) DNA-binding superfamily protein
Phvul.002G033500	Chr02	3,391,469	3,393,850	AT5G66460	MAN7, AtMAN7	ribosomal protein L5 B, Encodes a endo-beta-mannanase involved in seed germination.
Phvul.002G034900	Chr02	3,490,443	3,493,380	AT4G20770		PYR1-like 2
Phvul.002G037700	Chr02	3,651,792	3,654,086	AT4G33270	CDC20.1	cytochrome P450, family 711, subfamily A, polypeptide 1
Phvul.002G038600	Chr02	3,726,784	3,728,413	AT5G13930	CHS, TT4, ATCHS	myosin, putative, Encodes chalcone synthase (CHS), a key enzyme involved in the biosynthesis of flavonoids. Colorless seed coat.
Phvul.002G041800	Chr02	3,983,255	3,986,921	AT3G13960	AtGRF5, GRF5,	growth-regulating factor 5, Growth regulating factor encoding transcription activator during leaf development
Phvul.002G042800	Chr02	4,040,872	4,045,403	AT2G32010	CVP2	DNAse I-like superfamily protein
Phvul.002G045700	Chr02	4,238,272	4,245,844	AT4G14920		Acyl-CoA N-acyltransferase with RING/FYVE/PHD-type zinc finger protein
Phvul.002G048100	Chr02	4,480,988	4,483,345	AT5G28237		Pyridoxal-5'-phosphate-dependent enzyme family protein
Phvul.002G052000	Chr02	4,789,398	4,793,847	AT2G14740	ATVSR3, VSR3, VSR2	2, BP80-2
Phvul.002G054600	Chr02	4,962,933	4,972,324	AT1G76630		Tetratricopeptide repeat (TPR)-like superfamily protein
Phvul.003G102100	Chr03	25,054,795	25,061,749	AT5G18590		IQ-domain 21
Phvul.003G102300	Chr03	25,145,661	25,151,930	AT4G22760		Protein kinase superfamily protein
Phvul.003G102900	Chr03	25,282,673	25,292,563	AT3G21430		Protein kinase superfamily protein
Phvul.003G104100	Chr03	25,801,140	25,806,833	AT1G09040		non-ATPase subunit 9
Phvul.003G105800	Chr03	26,135,735	26,139,900	AT4G15820		

Gene	Chrom.	Start	End	Top *A. thaliana* hit (taken from Schmutz et al. (2014))	Gene symbol (taken from Schmutz et al. (2014))	Description(taken from Schmutz et al. (2014))
Phvul.003G106300	Chr03	26,361,983	26,363,099	AT5G18970		Protein of unknown function (DUF2921)
Phvul.003G110100	Chr03	27,714,817	27,718,947	AT4G16110	ARR1, RR1	response regulator 1
Phvul.003G110200	Chr03	27,746,661	27,749,302	AT4G16120	ATSEB1, COBL7, SEB1	COBRA-like protein-7 precursor
Phvul.003G110800	Chr03	27,855,253	27,855,639	AT1G24625		
Phvul.003G111200	Chr03	27,946,301	27,947,440	AT4G24220	VEP1, AWI31	NAD(P)-binding Rossmann-fold superfamily protein
Phvul.003G111400	Chr03	28,097,667	28,106,420	AT3G16910	AAE7, ACN1	acyl-activating enzyme 7
Phvul.003G111500	Chr03	28,143,701	28,144,951	AT1G13280	AOC3	allene oxide cyclase 3
Phvul.003G111600	Chr03	28,148,947	28,161,238	AT1G13270	MAP1C, MAP1B	methionine aminopeptidase 1B
Phvul.003G111900	Chr03	28,187,173	28,194,709	AT4G16150		calmodulin binding
Phvul.003G112000	Chr03	28,195,763	28,197,702	AT4G02080	ASAR1, ATSARA1C, ATSAR2, SAR2	secretion-associated RAS super family 2
Phvul.003G112600	Chr03	28,279,694	28,283,953	AT3G16980	NRPB9A, NRPD9A, NRPE9A	RNA polymerases M/15 Kd subunit
Phvul.003G113200	Chr03	28,370,732	28,373,556	AT4G15093		catalytic LigB subunit of aromatic ring-opening dioxygenase family
Phvul.003G113400	Chr03	28,431,965	28,433,734	AT3G16990		Haem oxygenase-like, multi-helical
Phvul.003G113500	Chr03	28,447,193	28,455,391	AT3G66658	ALDH22A1	aldehyde dehydrogenase 22A1
Phvul.003G113600	Chr03	28,473,899	28,474,612	AT2G24960		
Phvul.003G113700	Chr03	28,486,253	28,488,448	AT4G31980		1
Phvul.003G113800	Chr03	28,488,565	28,490,763	AT3G17000	UBC32	ubiquitin-conjugating enzyme 32
Phvul.003G114000	Chr03	28,538,083	28,539,739	AT2G23060		Acyl-CoA N-acyltransferases (NAT) superfamily protein
Phvul.003G114100	Chr03	28,555,015	28,559,491	AT3G17040	HCF107	high chlorophyll fluorescent 107
Phvul.003G114300	Chr03	28,599,004	28,603,037	AT3G06650	ACLB-1	ATP-citrate lyase B-1
Phvul.003G114800	Chr03	28,666,538	28,668,125	AT2G23540		GDSL-like Lipase/Acylhydrolase superfamily protein
Phvul.003G114900	Chr03	28,688,429	28,689,551	AT3G02970	EXL6	EXORDIUM like 6
Phvul.003G115000	Chr03	28,711,717	28,712,175	AT5G46950		
Phvul.003G115100	Chr03	28,762,324	28,764,580	AT4G16210	ECHIA, E-COAH-2	enoyl-CoA hydratase/isomerase A
Phvul.003G115200	Chr03	28,765,364	28,766,341	AT2G33770	UBC23, PFU2	ubiquitin-conjugating enzyme 23
Phvul.003G115600	Chr03	28,870,908	28,878,972	AT4G16180		unknown protein
Phvul.003G116100	Chr03	28,929,263	28,934,582	AT1G44900	MCM2, ATMCM2	minichromosome maintenance (MCM2/3/5) family protein
Phvul.003G116300	Chr03	29,019,242	29,021,426	AT5G49520	WRKY48, ATWRKY48	WRKY DNA-binding protein 48
Phvul.003G116400	Chr03	29,057,033	29,068,184	AT4G16280	FCA	RNA binding, Involved in the promotion of the transition of the vegetative meristem to reproductive development.
Phvul.003G116500	Chr03	29,073,228	29,073,797	AT3G17130		

Gene	Chrom.	Start	End	Top *A. thaliana* hit (taken from Schmutz et al. (2014))	Gene symbol (taken from Schmutz et al. (2014))	Description(taken from Schmutz et al. (2014))
Phvul.003G116600	Chr03	29,139,521	29,146,063	AT4G21800	QQT2	P-loop containing nucleoside triphosphate hydrolases superfamily protein
Phvul.003G116800	Chr03	29,203,626	29,206,938	AT3G17120		proteasome beta subunit C1
Phvul.003G117000	Chr03	29,212,353	29,214,624	AT4G16270		NAC (No Apical Meristem) domain transcriptional regulator superfamily protein
Phvul.003G117300	Chr03	29,228,042	29,228,584	AT1G47960	C/VIF1, ATC/VIF1	Eukaryotic aspartyl protease family protein
Phvul.003G117700	Chr03	29,317,826	29,320,068	AT2G05642		proteasome beta subunit C1
Phvul.003G117800	Chr03	29,374,518	29,380,656	AT3G06550		O-acetyltransferase family protein
Phvul.003G117900	Chr03	29,433,165	29,433,830	AT5G49600		Protein of unknown function, DUF538
Phvul.003G118200	Chr03	29,462,319	29,465,534	AT1G54140	TAFII21, TAF9	TATA binding protein associated factor 21kDa subunit
Phvul.003G124100	Chr03	30,403,201	30,405,704	AT5G28050		Cytidine/deoxycytidylate deaminase family protein
Phvul.003G124900	Chr03	30,542,597	30,550,021	AT5G17250		Alkaline-phosphatase-like family protein
Phvul.003G128700	Chr03	31,526,372	31,532,404	AT1G74960	FAB1, KAS2, ATKAS2	plant glycogenin-like starch initiation protein 2
Phvul.003G132400	Chr03	32,291,613	32,293,586	AT1G17455	ELF4-L4	ELF4-like 4
Phvul.003G133000	Chr03	32,372,901	32,379,152	AT5G13640	ATPDAT, PDAT, PDAT1	phospholipid:diacylglycerol acyltransferase
Phvul.003G133100	Chr03	32,387,633	32,393,330	AT1G72830	HAP2C, ATHAP2C, NF-YA3	nuclear factor Y, subunit A3
Phvul.003G134300	Chr03	32,513,289	32,525,859	AT4G29790		unknown protein
Phvul.003G134500	Chr03	32,565,767	32,571,105	AT5G57035		U-box domain-containing protein kinase family protein
Phvul.003G134600	Chr03	32,584,851	32,586,831	AT5G19890		Peroxidase superfamily protein
Phvul.003G136800	Chr03	32,871,397	32,873,558	AT4G29735		CONTAINS InterPro DOMAIN/s: Nuclear pore complex protein, Nucleoporin Nup85-like (InterPro:IPR011502)
Phvul.003G139300	Chr03	33,183,137	33,184,117	AT2G17220		
Phvul.003G139600	Chr03	33,224,000	33,227,068	AT5G57090	EIR1, WAV6, ATPIN2, PIN2, AGR, AGR1	Auxin efflux carrier family protein
Phvul.003G140300	Chr03	33,332,580	33,333,270	AT5G57120		FUNCTIONS IN: molecular_function unknown
Phvul.003G140600	Chr03	33,372,676	33,376,480	AT4G29920		Double Clp-N motif-containing P-loop nucleoside triphosphate hydrolases superfamily protein
Phvul.003G144500	Chr03	34,150,168	34,153,951	AT5G57390	AIL5, CHO1, EMK	AINTEGUMENTA-like 5, Encodes a member of the AP2 family of transcriptional regulators. May be involved in germination and seedling growth.
Phvul.003G145300	Chr03	34,359,645	34,365,381	AT5G57410		Afadin/alpha-actinin-binding protein
Phvul.003G145400	Chr03	34,396,977	34,402,363	AT4G30210	ATR2, AR2	P450 reductase 2
Phvul.003G145700	Chr03	34,433,529	34,439,156	AT5G57440	GS1, GPP2	appr-1-p processing enzyme family protein
Phvul.003G146000	Chr03	34,468,591	34,470,116	AT4G30230		unknown protein
Phvul.003G146600	Chr03	34,569,456	34,578,137	AT5G57490	VDAC4, ATVDAC4	voltage dependent anion channel 4
Phvul.003G146800	Chr03	34,613,147	34,613,533	AT5G57510		alpha/beta-Hydrolases superfamily protein

Gene	Chrom.	Start	End	Top *A. thaliana* hit (taken from Schmutz et al. (2014))	Gene symbol (taken from Schmutz et al. (2014))	Description(taken from Schmutz et al. (2014))
Phvul.003G148900	Chr03	34,930,116	34,933,878	AT4G15560	CLA1, DEF, CLA, DXS, DXPS2	Deoxyxylulose-5-phosphate synthase
Phvul.003G152800	Chr03	35,700,014	35,706,391	AT4G30510	ATATG18B, ATG18B, G18B	homolog of yeast autophagy 18 (ATG18) B
Phvul.003G152900	Chr03	35,710,737	35,716,123	AT4G30520		Leucine-rich repeat protein kinase family protein
Phvul.003G161500	Chr03	36,978,378	36,980,106	AT5G20050		Protein kinase superfamily protein
Phvul.003G161600	Chr03	36,984,946	36,989,328	AT4G17486		PPPDE putative thiol peptidase family protein
Phvul.003G161900	Chr03	37,005,928	37,009,369	AT5G41950		
Phvul.003G162400	Chr03	37,050,576	37,052,106	AT2G26695		Ran BP2/NZF zinc finger-like superfamily protein
Phvul.003G162700	Chr03	37,086,079	37,089,446	AT5G20070	ATNUDT19, ATNUDX19, NUDX19	amino acid permease 8
Phvul.003G162800	Chr03	37,090,077	37,091,792	AT1G15410		aspartate-glutamate racemase family
Phvul.003G162900	Chr03	37,097,975	37,103,846	AT5G20080		FAD/NAD(P)-binding oxidoreductase
Phvul.003G163000	Chr03	37,106,772	37,107,920	AT3G23880		F-box and associated interaction domains-containing protein
Phvul.003G163200	Chr03	37,115,208	37,115,985	AT1G15400		
Phvul.003G166000	Chr03	37,462,602	37,464,926	AT1G02630		Nucleoside transporter family protein
Phvul.003G171800	Chr03	38,277,301	38,280,551	AT5G34940	AtGUS3, GUS3	glucuronidase 3
Phvul.003G174800	Chr03	38,555,794	38,556,393	AT5G25240		unknown protein
Phvul.003G175100	Chr03	38,579,286	38,580,112	AT2G46490		unknown protein
Phvul.003G176300	Chr03	38,793,720	38,814,150	AT2G26890	GRV2, KAM2	DNAJ heat shock N-terminal domain-containing protein
Phvul.003G179300	Chr03	39,110,625	39,114,537	AT5G23090	NF-YB13	nuclear factor Y, subunit B13
Phvul.007G091300	Chr07	9,311,965	9,317,228	AT4G09510	CINV2	cytosolic invertase 2
Phvul.007G094200	Chr07	9,696,188	9,705,030	AT1G48850	EMB1144	chorismate synthase, putative / 5-enolpyruvylshikimate-3-phosphate phospholyase, putative
Phvul.007G094300	Chr07	9,724,193	9,729,678	AT2G39220	PLP6, PLA IIB	PATATIN-like protein 6
Phvul.007G094400	Chr07	9,770,994	9,772,378	AT5G19290		alpha/beta-Hydrolases superfamily protein
Phvul.007G094700	Chr07	9,827,559	9,844,197	AT5G20660		Zn-dependent exopeptidases superfamily protein
Phvul.007G094800	Chr07	9,845,911	9,848,855	AT2G39000		Acyl-CoA N-acyltransferases (NAT) superfamily protein
Phvul.007G095000	Chr07	9,869,289	9,872,915	AT4G30080	ARF16	auxin response factor 16
Phvul.007G095100	Chr07	9,882,588	9,884,606	ATMG00300		
Phvul.007G095300	Chr07	9,922,891	9,926,868	AT3G54810	GATA8	GATA transcription factor 9, Encodes a protein containing a GATA type zinc finger domain that is expressed in the embryo axis and involved in germination. Mutants have a reduced rate of germination even when stratified.
Phvul.007G095600	Chr07	9,983,235	9,985,678	AT5G03250		Phototropic-responsive NPH3 family protein

Gene	Chrom.	Start	End	Top *A. thaliana* hit (taken from Schmutz et al. (2014))	Gene symbol (taken from Schmutz et al. (2014))	Description(taken from Schmutz et al. (2014))
Phvul.007G095700	Chr07	9,987,742	9,988,152	AT4G02210		
Phvul.007G095900	Chr07	9,996,844	9,998,891	AT4G14145		unknown protein
Phvul.007G096500	Chr07	10,156,828	10,169,477	AT5G04240	ELF6, Early Flowering 6 (ELF6)	Zinc finger (C2H2 type) family protein / transcription factor jumonji (jmj) family protein
Phvul.007G096600	Chr07	10,171,001	10,172,702	AT3G57120		Protein kinase superfamily protein
Phvul.007G096700	Chr07	10,173,474	10,175,845	AT3G54826		Zim17-type zinc finger protein
Phvul.007G096800	Chr07	10,184,754	10,196,029	AT3G10350		P-loop containing nucleoside triphosphate hydrolases superfamily protein
Phvul.007G097100	Chr07	10,248,037	10,254,281	AT3G10360	APUM2, PUM2	
Phvul.007G097200	Chr07	10,278,772	10,289,556	AT2G39130		Transmembrane amino acid transporter family protein
Phvul.007G097500	Chr07	10,403,778	10,406,331	AT4G02550		
Phvul.007G097800	Chr07	10,421,422	10,421,880	AT3G11110		
Phvul.007G097900	Chr07	10,424,422	10,424,855	AT5G46650		
Phvul.007G098000	Chr07	10,428,439	10,428,651	AT3G11110		
Phvul.007G098700	Chr07	10,512,291	10,516,538	AT3G54850	ATPUB14, PUB14	plant U-box 14
Phvul.007G098800	Chr07	10,517,794	10,543,382	AT3G10380	SEC8, ATSEC8	subunit of exocyst complex 8
Phvul.009G123600	Chr09	18,328,832	18,334,183	AT4G08290		nodulin MtN21 /EamA-like transporter family protein
Phvul.009G124700	Chr09	18,518,859	18,523,474	AT1G77610		Chitinase family protein
Phvul.009G125200	Chr09	18,576,069	18,580,983	AT1G77580		Plant protein of unknown function (DUF869)
Phvul.009G128500	Chr09	18,981,425	18,986,617	AT1G77380	AAP3, ATAAP3	expansin A17
Phvul.009G129300	Chr09	19,041,215	19,045,877	AT1G21651	SECA2	Preprotein translocase SecA family protein
Phvul.009G129400	Chr09	19,047,225	19,068,103	AT1G21650	SECA2	Preprotein translocase SecA family protein
Phvul.009G129700	Chr09	19,107,827	19,110,343	AT1G21640	NADK2, ATNADK2	unknown protein
Phvul.009G129800	Chr09	19,116,028	19,125,106	AT1G21610		wound-responsive family protein
Phvul.009G130400	Chr09	19,193,741	19,195,132	AT4G37925	NDH-M	subunit NDH-M of NAD(P)H:plastoquinone dehydrogenase complex
Phvul.009G130500	Chr09	19,201,311	19,209,056	AT5G52560	ATUSP, USP	UDP-sugar pyrophosphorylase
Phvul.009G178600	Chr09	26,178,717	26,180,114	AT5G62770		Galactose oxidase/kelch repeat superfamily protein
Phvul.009G181300	Chr09	26,705,923	26,708,734	AT5G62575		unknown protein
Phvul.009G184500	Chr09	27,179,214	27,183,146	AT1G74360		Leucine-rich repeat protein kinase family protein
Phvul.009G184600	Chr09	27,211,707	27,214,154	AT1G13950	EIF-5A, ELF5A-1, ATELF5A-1, EIF5A	eukaryotic elongation factor 5A-1
Phvul.009G184700	Chr09	27,230,379	27,238,422	AT1G18670	IBS1	basic helix-loop-helix (bHLH) DNA-binding superfamily protein
Phvul.009G184800	Chr09	27,263,086	27,280,562	AT5G07740		actin binding

Gene	Chrom.	Start	End	Top *A. thaliana* hit (taken from Schmutz et al. (2014))	Gene symbol (taken from Schmutz et al. (2014))	Description(taken from Schmutz et al. (2014))
Phvul.009G184900	Chr09	27,282,877	27,283,863	AT5G61340		unknown protein
Phvul.009G185000	Chr09	27,290,665	27,299,757	AT1G18660		zinc finger (C3HC4-type RING finger) family protein
Phvul.009G185100	Chr09	27,343,323	27,354,415	AT1G68710		ATPase E1-E2 type family protein / haloacid dehalogenase-like hydrolase family protein
Phvul.009G185300	Chr09	27,376,113	27,381,761	AT5G39510	VTI12, ATVTI12, VTI1B	Vesicle transport v-SNARE family protein
Phvul.009G185500	Chr09	27,386,737	27,392,574	AT5G61380	TOC1, APRR1, PRR1, AtTOC1	CCT motif -containing response regulator protein
Phvul.009G185600	Chr09	27,398,251	27,402,842	AT5G61400		Pentatricopeptide repeat (PPR) superfamily protein
Phvul.009G185900	Chr09	27,425,173	27,453,079	AT1G18700		DNAJ heat shock N-terminal domain-containing protein
Phvul.009G189600	Chr09	28,126,046	28,128,810	AT5G62550		unknown protein
