# Supplementary figures and images for: Testing Domestication Scenarios of Lima Bean (Phaseolus lunatus L.) in Mesoamerica: Insights from Genome-Wide Genetic Markers
Source: Front Plant Sci. 2017 Sep 12;8:1551. doi: 10.3389/fpls.2017.01551 (PMC5601060; doi:10.3389/fpls.2017.01551)

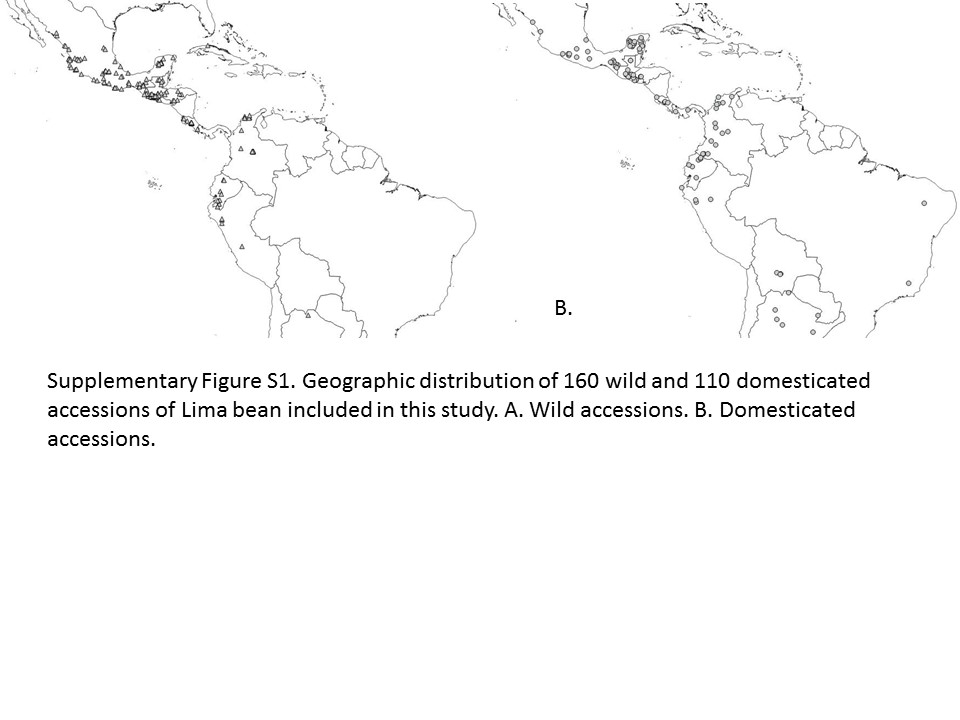

Supplement: Supplementary file 10 [file Image1.JPEG]

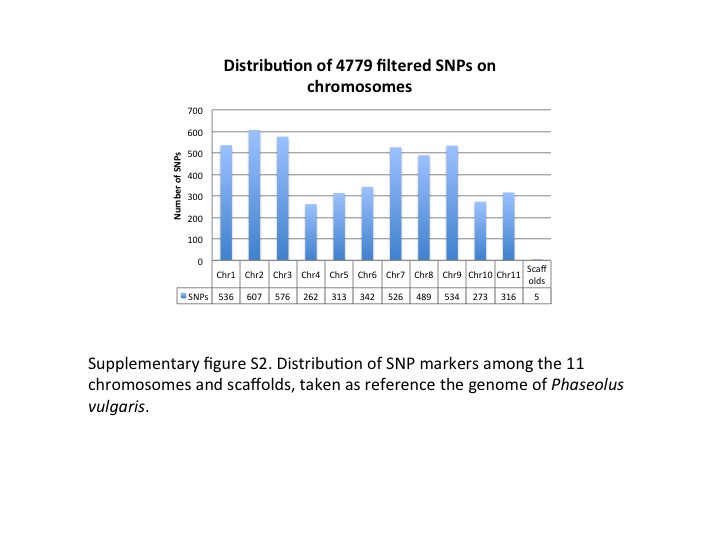

Supplement: Supplementary file 11 [file Image2.JPEG]

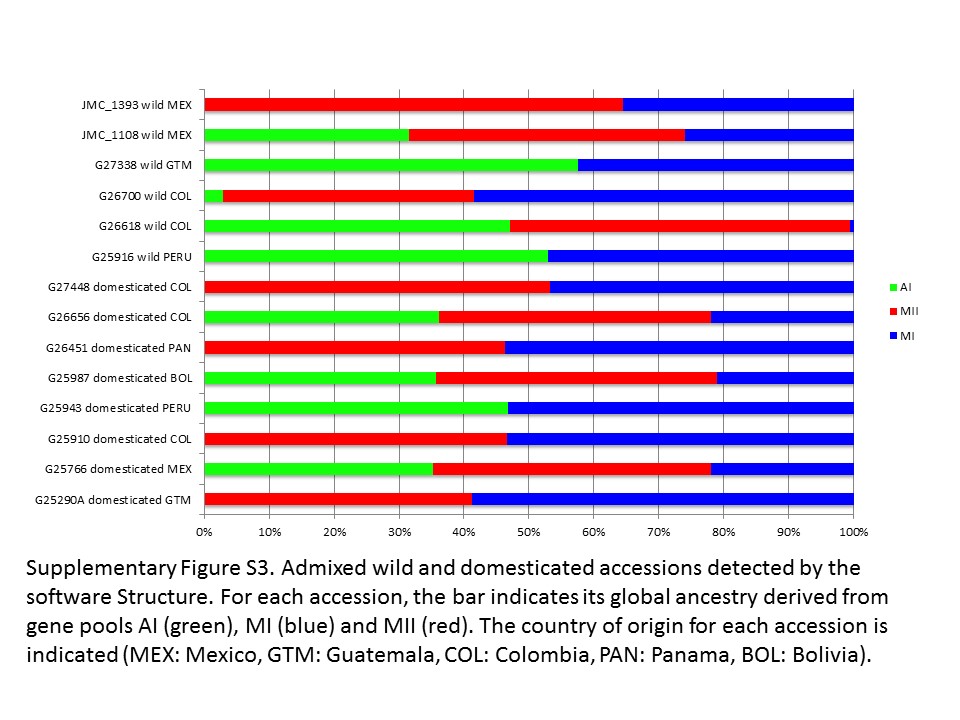

Supplement: Supplementary file 12 [file Image3.JPEG]

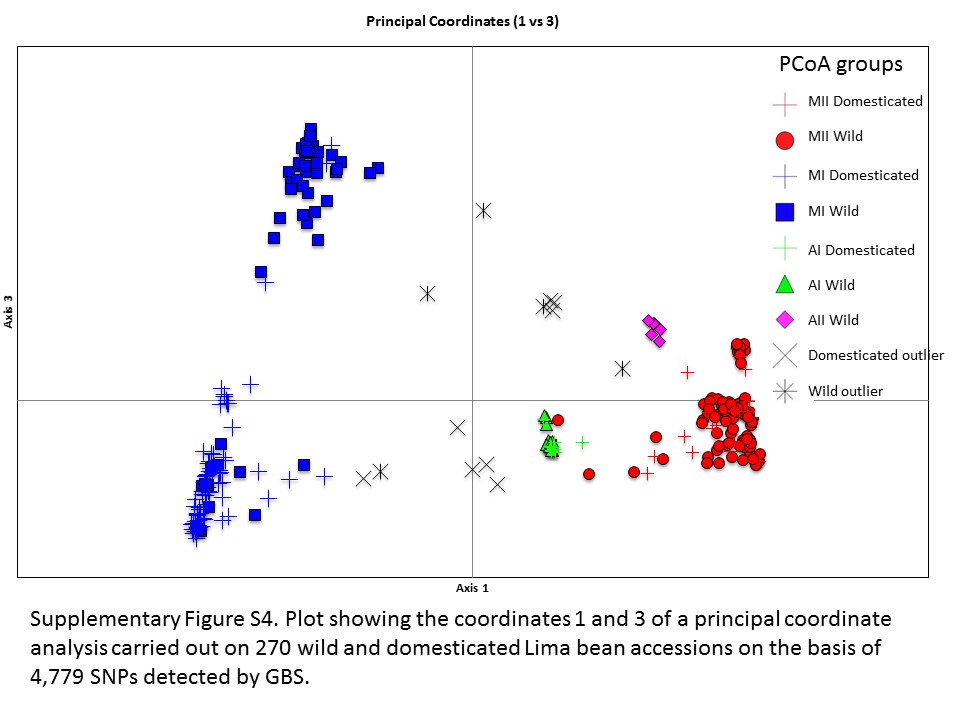

Supplement: Supplementary file 13 [file Image4.JPEG]

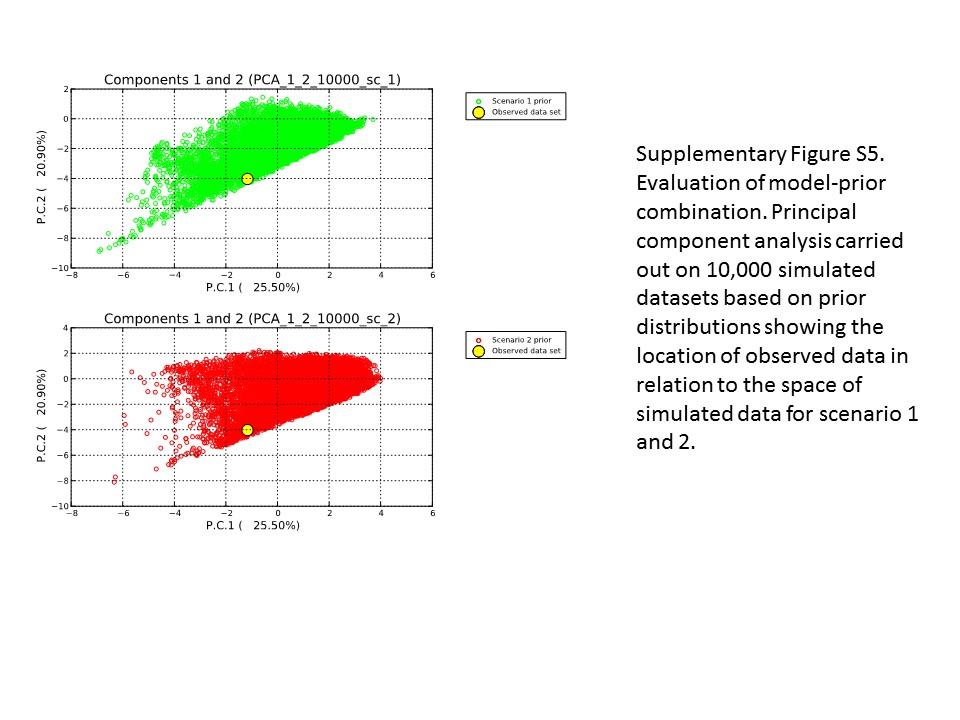

Supplement: Supplementary file 14 [file Image5.JPEG]

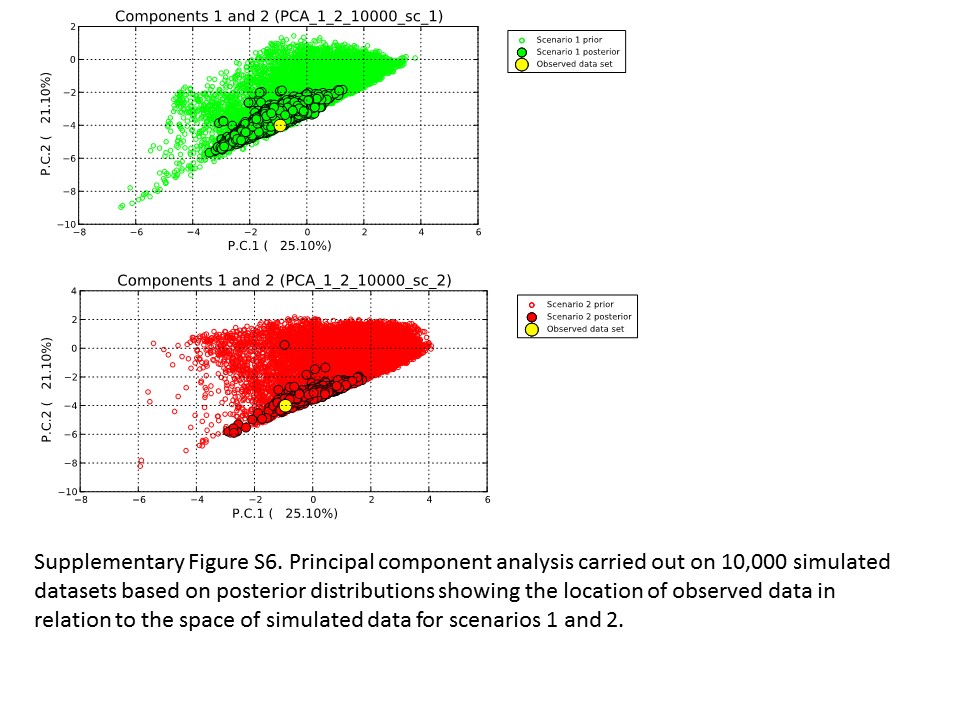

Supplement: Supplementary file 15 [file Image6.JPEG]

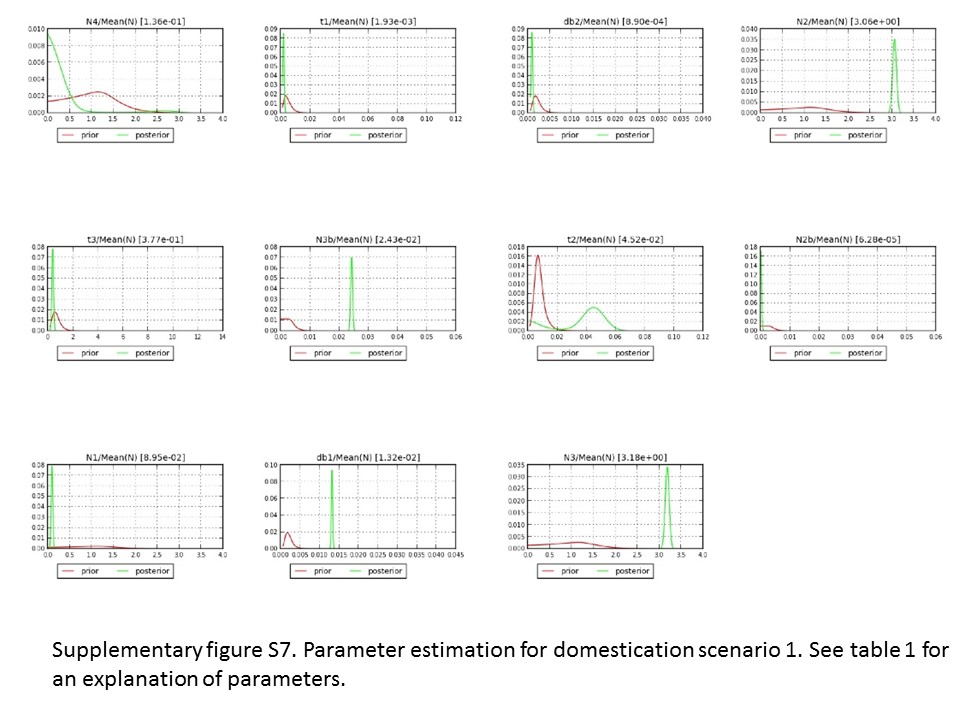

Supplement: Supplementary file 16 [file Image7.JPEG]

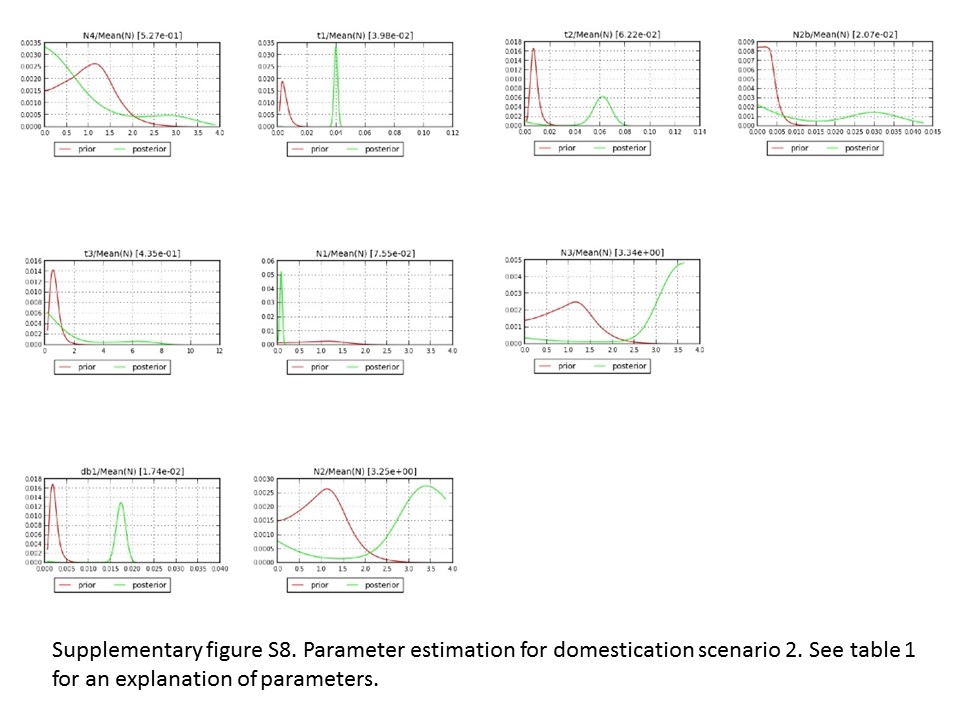

Supplement: Supplementary file 17 [file Image8.JPEG]

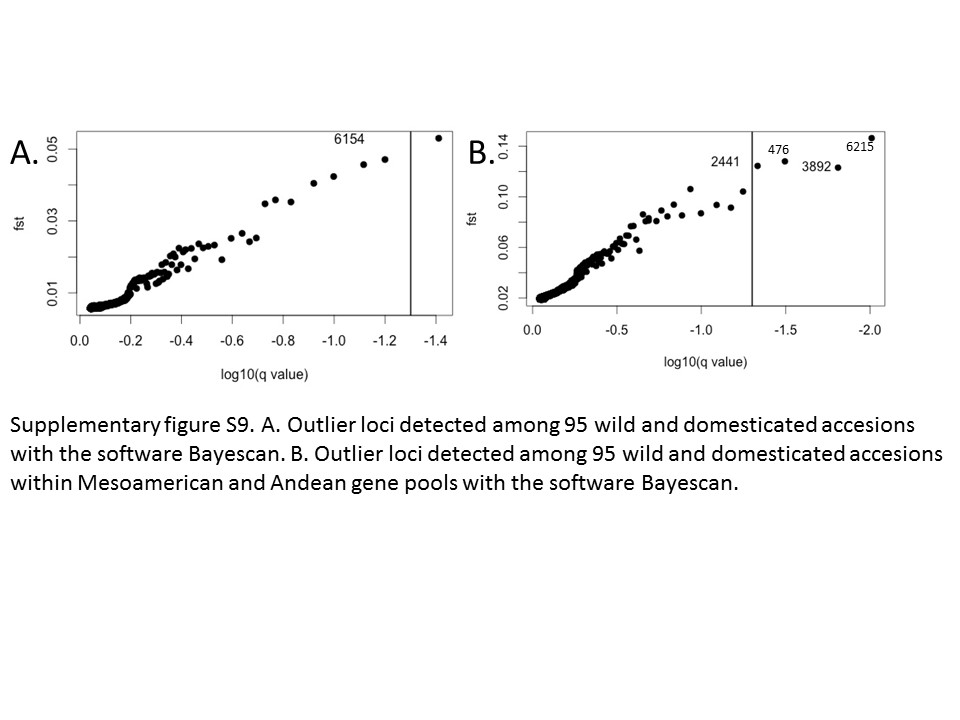

Supplement: Supplementary file 18 [file Image9.JPEG]
